# Weak dispersal and landscape size inevitably promote local biodiversity in heterogeneous metacommunities of competing species

**DOI:** 10.1371/journal.pcbi.1014028

**Published:** 2026-10-16

**Authors:** Frederik De Laender, Andrew Gonzalez, Olivia Bleeckx, Dieter Ebert, György Barabás

**Affiliations:** 1 Research Unit of Environmental and Evolutionary Biology, naXys, ILEE, University of Namur, Namur, Belgium; 2 Department of Biology, McGill University, Montreal, Canada; 3 Department of Biology, University of Namur, Namur, Belgium; 4 Department of Environmental Sciences, Zoology, University of Basel, Basel, Switzerland; 5 Tvärminne Zoological Station, Hanko, Finland; 6 Division of Biology, Linköping University, Linköping, Sweden; 7 Institute of Evolution, Centre for Ecological Research, Budapest, Hungary; Center for Advanced Systems Understanding (CASUS), GERMANY

## Abstract

We provide a general theoretical explanation for a longstanding result in spatial ecology: weak dispersal among habitat patches promotes local species richness. Using analytical approximations of spatial Lotka–Volterra competition models, we show that local species persistence in heterogeneous landscapes can be expressed as the balance between the total density across patches and the local exclusion rate. We further demonstrate that local multispecies coexistence is governed by the feasibility domain, linking spatial coexistence to a structural property of nonspatial competitive systems. Together, these results explain why weak dispersal increases local species richness and why this effect strengthens with the number of available patches, i.e., landscape size. We test these predictions using numerical simulations and find that the theory breaks down only when both dispersal and competitive interactions are very strong, in which case dispersal has a unimodal effect on coexistence. In contrast, landscape size retains a positive effect on coexistence whenever an effect is detectable. We then apply the theory to long-term data from a natural metacommunity of microcrustaceans. We detect strong competition among species and find no detectable effect of dispersal rate on local coexistence, whereas species co-occurrence increases with local landscape size, as predicted by theory. Together, our results identify how dispersal, interaction strength, and landscape size jointly regulate local species richness in competitive systems.

## Author summary

We study a long-standing question in ecology: why does a small amount of movement between habitats often increase the number of species that can live together locally? Although this pattern has been observed in many natural systems, a general explanation has been difficult to establish for communities containing many competing species. Using a mathematical model of species competition across connected habitats, we show that local coexistence depends on a simple balance between two forces. Species are more likely to persist in a habitat when they are abundant across the broader landscape and less likely to persist when strong competition would otherwise drive them locally extinct. This balance allows us to predict when dispersal among habitats promotes biodiversity and when it does not. We also show that the chance that all species coexist locally is closely linked to existing ecological theory developed for isolated communities. We test these predictions using computer simulations and long-term observations of microcrustaceans living in Finnish rock pools. We find strong agreement between theory and data, but also reveal the ecological situations in which it breaks down. Our results provide a unified framework linking dispersal, competition, landscape size, and biodiversity across spatially connected ecosystems.

## Introduction

The effect of dispersal–-the movement of organisms from one location to another–on local species richness depends on a variety of factors. Spatial heterogeneity in environmental conditions [1,2], the strength of local species interactions [3,4], and the directionality [5], capacity [6], and density-dependence of dispersal rates [7], all have been reported as significant factors. However, despite this multitude of factors shaping the effect of dispersal on local species richness, one finding consistently emerges from most experimental [8,9] and modeling studies [10–14], as well as from meta-analyses [15,16]: in heterogeneous landscapes, weak dispersal tends to promote local species richness.

The verbal explanation for weak dispersal affecting local richness positively is that weak dispersal alleviates local species interactions, which thence increases local richness. Recent theory has further shown that species abundances in open communities can often be understood as a balance between immigration and local exclusion [17], although the implications of this balance for local coexistence across spatial networks remain unclear. Simulation models have played an important role in providing numerical evidence for this mechanism [11,18]. One approach extends the classical Lotka–Volterra equations for *n* interacting species with dispersal [12,19]. These models track the population density $N_{i}^{\left(l\right)}$ of every species *i* in every patch *l*, across a spatial network of *p* patches inhabited by *n* interacting species:



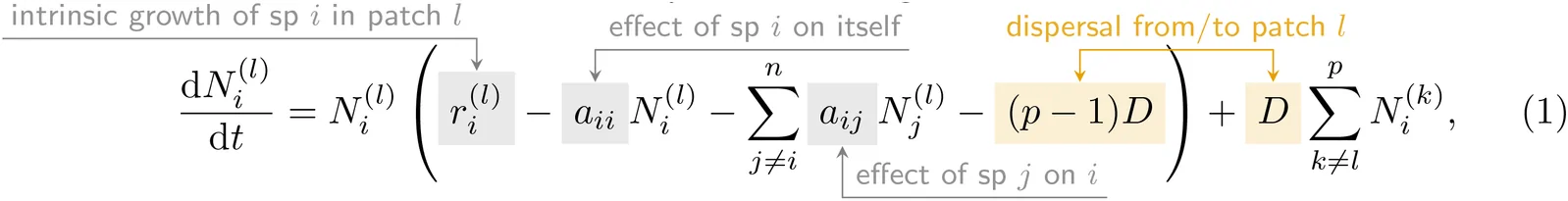



where $r_{i}^{\left(l\right)}>0$ is the intrinsic growth rate of species *i* in patch *l*, $a_{ii}=1$ is the intraspecific effect (set to unity by convention), $a_{ij}$ is the effect of species *j* on *i*, and *D* is the dispersal rate, i.e., the per-capita rate at which individua*l*s leave one patch and enter another patch. Throughout this manuscript, we refer to *p* as landscape size. Note that Eq. 1 by design embeds two simplifying assumptions: (i) dispersal is well-mixed (any pair of patches exchanges individuals at the same rate *D*), and (ii) species interactions do not depend on patch number *l*.

An important drawback of Eq. 1 is that it is analytically intractable for diverse communities and thus hard to draw general insights from. Available analytical results for this model therefore focus on communities with few species [10,20,21], highlighting how spatial heterogeneity in competitive ranking can enable coexistence [10], and how different dispersal mechanisms can modify coexistence outcomes [7]. Other results of models based on Eq. 1 ignore the discrete nature of the spatial network by relying on a reaction-diffusion formalism [22,23]. One exception is the contribution by Levin [3], showing that dispersal promotes invasion into a patch if the local conditions of that patch permit growth of the invader. How these results relate to long-term persistence has remained unclear, however. More parsimonious and therefore analytically tractable alternatives to Eq. 1 include patch occupancy community models [24–27], where one tracks the fraction of patches that species occupy in a landscape. However, this research program cannot explain why weak dispersal has a positive effect on local richness, because it focuses on persistence at the landscape level, not on local abundances [2]. In summary, to date, there is no general explanation of the positive effect of weak dispersal on local richness in large communities.

Here, we present new analyses that provide a quantitative explanation for the well-documented positive effect of weak dispersal on local species persistence in heterogeneous landscapes. Because weak dispersal allows us to truncate the power-series expansion around the no-dispersal equilibrium after the linear term, we can analytically solve Eq. 1. Doing so, we first recover the result that weak dispersal inevitably promotes persistence (measured by the fraction of patches in which a randomly selected species has positive density). This effect is stronger in larger-sided landscapes, i.e., landscapes with more patches, and weaker interactions. We then show the implications of these results for coexistence, quantified via the fraction of patches in which all *n* regionally available species coexist. We show how this multispecies extension of the patch occupancy concept, initially developed for metapopulations [25,27–29], relates to feasibility, a classic quantity in contemporary coexistence theory [30,31]. Further, we show that persistence and patch occupancy are analytically predictable when the mean growth rate across all patches is the same for all species (i.e., species are regionally equivalent), and all interspecific interactions are exactly equal (diffuse species interactions [32,33],). Model simulations show that this prediction is exact, and that our conclusions continue to qualitatively hold when relaxing our analyses’ underlying assumptions, unless interspecific interactions are strong and dispersal is so large as to have a nonlinear effect on population densities. In that case, dispersal can have a unimodal effect on patch occupancy. Landscape size, however, continues to promote patch occupancy, even when interactions are strong. Finally, we compare our findings with a metacommunity of three microcrustacean species of the genus *Daphnia*, sampled across hundreds of rockpools on the Tvärminne archipelago (Finland). This long-term dataset (twice-yearly censuses since 1982) is well-suited to our framework: pools form a spatially explicit and environmentally heterogeneous metacommunity in which dispersal is mediated by wind-driven transport of resting eggs after pool desiccation. The dataset allows us to test the qualitative prediction that local landscape size promotes co-occurrence. Our analysis of these data suggests strong interspecific interactions and shows that species co-occurrence is not affected by a dispersal proxy but more likely in pools surrounded by a greater number of pools.

## Materials and methods

### Analyses

#### Weak dispersal approximation.

Moving from observing to understanding the roles of dispersal and species interactions is challenging because mathematically analyzing Eq. 1 is not trivial. To solve this problem, we approximate the equilibrium density of species *i* in some patch *l*, ${\hat{N}}_{i}^{\left(l\right)}$, by a Taylor series in *d*, where *d* equals *D* after rescaling. This rescaling is needed to remove units and permit this approximation (S1 Appendix). If dispersal is weak, when 0≤D≪1, we can ignore higher-order terms in this series and write ${\hat{N}}_{i}^{\left(l\right)}$ as the sum of what its density would have been without dispersal (which we call ${\hat{N}}_{0,i}^{\left(l\right)}$, where the 0 subscript denotes absence of dispersal, used throughout the manuscript) and the first-order (in *d*) correction (which we call ${\hat{N}}_{1,i}^{\left(l\right)}$), leading to ${\hat{N}}_{i}^{\left(l\right)}\approx {\hat{N}}_{0,i}^{\left(l\right)}+d{\hat{N}}_{1,i}^{\left(l\right)}$. The term $d{\hat{N}}_{1,i}^{\left(l\right)}$ is a correction of the local density to account for weak amounts of dispersal. Setting Eq. 1 to zero and replacing ${\hat{N}}_{i}^{\left(l\right)}$ with its approximation leads to an implicit equation for ${\hat{N}}_{1,i}^{\left(l\right)}$ (S1 Appendix), which we analyze to study how dispersal affects the probability of local persistence and coexistence.

#### Decomposition of persistence and coexistence probability.

We define the probability of local species persistence as the fraction of patches in which a focal species *i* persists, which is equivalent to the probability that a random species persists in a random patch (i.e., the probability that ${\hat{N}}_{i}^{\left(l\right)}>0$). We partition this probability into two ecologically relevant cases, using the law of total probability:


$$\begin{array}{c} P({\hat{N}}_{i}^{\left(l\right)}>0)=\underset{\text{persistence, case extinction w/o dispersal}}{\underbrace{P({\hat{N}}_{i}^{\left(l\right)}>0|{\hat{N}}_{0,i}^{\left(l\right)}=0)\cdot P({\hat{N}}_{0,i}^{\left(l\right)}=0)}}+\underset{\text{persistence, case persistence w/o dispersal}}{\underbrace{P({\hat{N}}_{i}^{\left(l\right)}>0|{\hat{N}}_{0,i}^{\left(l\right)}>0)\cdot P({\hat{N}}_{0,i}^{\left(l\right)}>0)}}. \end{array}$$ (2)

The first term describes persistence of species that would go extinct without dispersal; the second term describes persistence of species that already persist without dispersal.

The natural community-level analogue is the probability that all *n* regionally available species coexist in a randomly drawn patch, which we partition by the same logic:



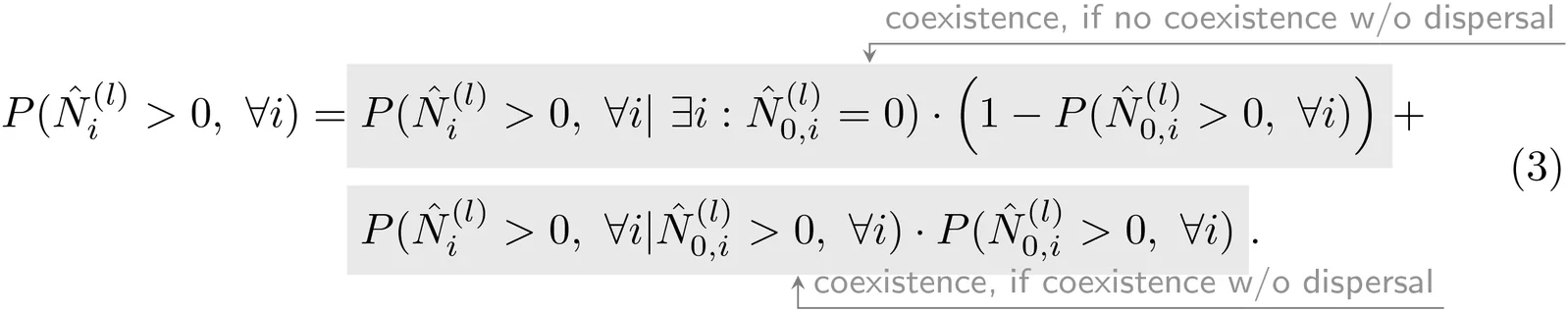



where $P({\hat{N}}_{0,i}^{\left(l\right)}>0,\;\forall i)$ is the probability that all species have positive density in absence of dispersal.

#### Calculation of persistence and coexistence probability.

To obtain explicit expressions for the conditional probabilities appearing in Eqs. 2 and 3, given spatial variation of the intrinsic growth rates $r_{i}^{\left(l\right)}$, we introduced the following simplifying assumptions.

First, all the $r_{i}^{\left(l\right)}$ are i.i.d. random variables following a standard normal distribution, truncated to the positive real numbers, and standardized such that the mean across species within any given patch, and the mean across patches for any given species, are both 1. This introduces spatial heterogeneity because species grow differently in different patches (as in [34]), but also implies that species are regionally equivalent: the arithmetic mean of $r_{i}^{\left(l\right)}$ across patches is identical for every species (while each species is free to have its largest growth rate in one or several patches), as illustrated in Fig 1. This assumption makes $P({\hat{N}}_{0,i}^{\left(l\right)}>0,\;\forall i)$ from Eq. 3 equivalent to the *feasibility domain* Ξ(n) of an *n*-species community. This is the fraction of growth-rate vectors **r** for which the equilibrium is an interior equilibrium: all densities are strictly positive. Technically speaking, it is the solid angle spanned by the columns of the interaction matrix $𝐀=(a_{ij})$ on the positive orthant of the *n*-dimensional unit sphere, divided by the total volume of that orthant [30,31].

**Fig 1:**
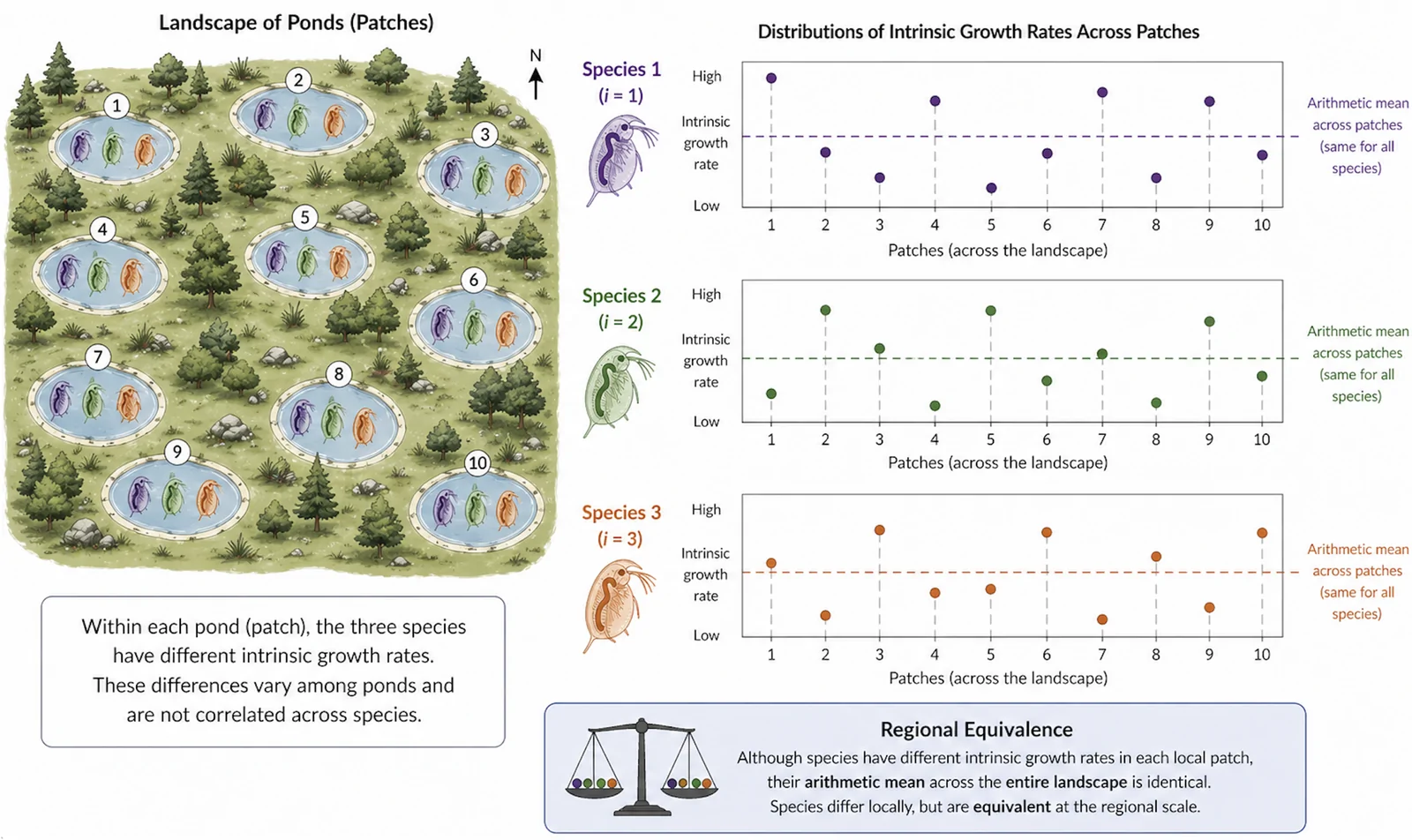
Regional equivalence, illustrated for a pond metacommunity. A landscape of 10 pond patches inhabited by three water flea species illustrates the concept of *regional equivalence*. Intrinsic growth rates vary among patches and differ within a given pond. Although species exhibit distinct, non-covarying spatial patterns in patch-specific growth rates $r_{i}^{\left(l\right)}$, their arithmetic mean across the landscape is identical for all species. Thus, species differ locally but are equivalent when averaged at the regional scale. The diagram was generated using ChatGPT (OpenAI Terms of Use).

Second, we assume a diffuse interaction scheme: all pairwise species interactions are exactly equal ($a_{ij}=a$ for all i≠j) and weaker than intraspecific interactions ($a<a_{ii}=1$ for all *i*).

We then calculated the conditional probabilities using the method specified in S1 Appendix, for a regional pool of *n* species (with *n* = 10) inhabiting landscapes of sizes *p* = 10–50 patches, dispersal rates between 10^–6^ to 10^–2^, and interaction coefficients *a* of 0.2 and 0.5.

### Simulations

We ran simulations with an extended model (S1 Appendix) to assess the robustness of our analytical predictions when relaxing assumptions of Eq. 1 (well-mixed dispersal and spatially uniform species interactions) and assumptions used in its analysis (regional equivalence and diffuse interspecific interactions weaker than intraspecific interactions). We used a fully factorial design varying landscape size *p* (10–50), dispersal *d* (10^–6^–10^–1^), mean interspecific interaction strength $\overset{―}{a}=1/n\sum_{j\neq i}^{n}a_{ij}$ (0.2, 0.5, or 1), variation in interspecific interaction strength across pairs (coefficients of variation of 0 or 0.2), spatial variation in interactions (absent or present), regional equivalence of growth rates (present or absent), and dispersal structure (well-mixed or following an exponentially declining dispersal kernel). The combination of weak dispersal, $\overset{―}{a}<1$, no variation in interaction strength, spatially uniform interactions, regional equivalence, and well-mixed dispersal corresponds to the assumptions underlying our analytical results and therefore provides a direct comparison with their predictions. Note that $\overset{―}{a}\geq 1$ represents preemptive competition, whereby species arriving first exclude subsequent invaders [21]. We ran 100 replicate simulations for each factorial combination, with *n* = 10 regionally available species.

To introduce spatial variation in interpecific species interactions, we multiplied each interaction coefficient by an independent draw from *U*[0.8,1.2]. To break regional equivalence, we multiplied the regional mean growth rate of one randomly selected species by 1.5 and divided that of the other species by the same factor. For spatially structured dispersal, we assigned patches random coordinates within a unit square and each species a characteristic dispersal distance c~U[0.5,4]. Dispersal between two patches separated by distance *s* was proportional to *e*^–*s*/*c*^, with rates standardized so that their mean equaled that of the corresponding well-mixed scenario.

For each factorial combination and replicate, we sampled strictly positive local growth rates by taking the absolute values of *pn* draws from a standard normal distribution and iteratively normalized them so that both the mean across species within each patch and the mean across patches for each species equaled 1. Species were initialized at one tenth of their local carrying capacity, and metacommunity dynamics were simulated to equilibrium using rootSolve [35,36] in R [37]. Species with final densities below 10^–3^ were considered extinct. We calculated patch occupancy as the fraction of patches in which all *n* species persisted.

To facilitate comparison with the analytical results and be able to tell what levels of *d* can be considered sufficiently weak, we ran an additional simulation to evaluate within which range *d* had a linear effect on local density. The detailed simulation protocol is in S1 Appendix.

### *Daphnia* metacommunity

*Daphnia* are small (0.5–5 mm) planktonic microcrustaceans that reproduce clonally (parthenogenetically) during the ice-free season and switch to the production of desiccation- and freezing-resistant resting eggs (ephippia) at the end of the season or when the pool dries out [38]. Interactions among *Daphnia* species tend to be strong, with preemptive competition, the monopolization of pools by the first colonizing species or genotype, a common outcome in both natural lake records and controlled experiments [39,40], though not previously established for this Tvärminne metacommunity itself.

The occurrence of three *Daphnia* species (*D. magna*, *D. pulex*, *D. longispina*) in 567 rock pools across 17 islands of the Tvärminne archipelago (Finland) has been censused twice per year since 1982 [41–43]. These islands form seven spatial clusters (map in Fig A in S1 Appendix). We attributed islands to a cluster based on proximity: islands closer than 100 meters to each other were considered as a single cluster. We assumed dispersal between two island clusters is negligible. Within clusters, pools are connected by wind-driven dispersal of resting eggs following pool desiccation [38]. We used simulated desiccation rates for every pool and year from Altermatt et al. [44] as a proxy of the dispersal rate. This was only possible for observations until 2006.

We carried out two complementary analyses on these data. Given their important role for local coexistence in metacommunities, and their commonness among *Daphnia* species, we first evaluated the prevalence of preemptive competition [10,21] among species, since confirming it directly in our own data–-rather than relying on inference from other systems–-determines how we parameterize the system-specific simulations below (i.e., $\overset{―}{a}\geq 1$ vs. $\overset{―}{a}<1$). Our second objective was to test if the probability of observing a species pair in a pool is greater when the pool has more neighboring pools, and has experienced more frequent desiccation events. We focus on species pairs because co-occurrence of all three species was very rare, having been observed in only 0.33% of all sampling events. After removing observations without matching desiccation data, and one island for which we only had a single observation for the preemptive competition analysis, we retained data on 401 pools across 14 islands.

To address our first objective, we retained only those pools that contained a single focal species in spring, and analyzed community composition in the subsequent summer. We identified four potential summer compositions: Same composition as in spring (1); Replacement of the focal species by one species (2); Replacement of the focal species by two species (3); Presence of two species, including the focal species (4). We modeled the probability for case (1) to occur, since if case (1) would be most frequent for each of the three focal species, this would be evidence for preemptive competition. We only used data of pools that were relatively isolated, i.e., had less than 20 surrounding pools within a 100 m radius, assuming these would better reflect the outcome of local dynamics. To these data, we fitted a Bayesian binomial generalized linear mixed model (GLMM) with a logit link function for each species. The response variable was binary: 0 (focal not present as only species in summer) or 1 (focal present as only species in summer). We modeled $p_{A,ijt}$, the probability of observing the focal species *A* as the only species in the *j*^th^ pool of the *i*^th^ island in summer of year *t*. The fixed effect was the presence of the focal species as *t*he single species in spring of the same year and in the same pool. We included island, pool identity (nested within island), and year as random effects:


$$\begin{array}{cc} Y_{A,ijt} & \sim Bernoulli(p_{A,ijt}) \end{array}$$ (4)

$$\begin{array}{cc} logit(p_{A,ijt}) & =\beta_{0}+\beta_{1}(\text{spring presence}{)}_{A,ijt}+u_{i}+v_{ij}+w_{t}\;. \end{array}$$ (5)

where $u_{i}\sim 𝒩(0,\sigma_{u}^{2})$, $v_{ij}\sim 𝒩(0,\sigma_{v}^{2})$, and $w_{t}\sim 𝒩(0,\sigma_{w}^{2})$. We relied on the default priors offered by the brms [45] package: improper uniform (flat) functions for all means, and half-Student t-distributions for all standard deviations. The latter always had 3 degrees of freedom, location parameter zero, and scale parameter equal to 2.5 times the standard deviation of the response variable after applying the logit link function.

To address our second objective, an intuitive approach would have been to use the number of pools within a cluster as a predictor of the fraction of pools where species coexist. However, this has as a caveat that pool density differed per cluster, possibly introducing important differences in dispersal rates between them. We therefore instead refocused our analysis on the pool level, asking if co-occurrence was more likely in pools surrounded by a greater number of other pools and in clusters with higher desiccation rates (and therefore presumably higher dispersal rates). Within each cluster, we measured the distance between each pair of pools and counted, for each pool, the number of neighbor pools within a given distance. We carried out our statistical analysis for distances of 50 and 100 meters to test the robustness of the obtained results. Our statistical method consisted of fitting a Bayesian binomial generalized linear mixed model (GLMM) with a logit link function. The response variable was binary: 0 (single species present in a pool) or 1 (pair present in a pool). We modeled $p_{ijkt}$, the probability of observing a species pair in the *j*^th^ pool on the *i*^th^ island, belonging to cluster *k*, in year *t*. Fixed effects were the number of surrounding pools ($P_{ij}$; constan*t* across years for each pool) and the simulated desiccation rate ($S_{kt}$; varying among clusters and years). We included island, pool identity (nested within island), and year as random effects to account for the hierarchical structure of the sampling design:


$$\begin{array}{cc} Y_{ijkt} & \sim Bernoulli(p_{ijkt}) \end{array}$$ (6)

$$\begin{array}{cc} logit(p_{ijkt}) & =\beta_{0}+\beta_{1}P_{ij}+\beta_{2}S_{kt}+u_{i}+v_{ij}+w_{t}\; \end{array}$$ (7)

where $u_{i}\sim 𝒩(0,\sigma_{u}^{2})$, $v_{ij}\sim 𝒩(0,\sigma_{v}^{2})$, and $w_{t}\sim 𝒩(0,\sigma_{w}^{2})$. We fitted this model for both the spring and summer samples and for the two distances (*b* = 50 m and 100 m). Like for our first objective, we used the R [37] package brms [45] for model fitting, with the same default priors as before.

Finally, we applied the metacommunity model to the empirical system to test if the predictions continued to hold when parameterizing the model for a natural metacommunity. To this end, we ran an additional set of simulations with the extended model (S1 Appendix), parameterized to mimic the Tvärminne rock-pool *Daphnia* metacommunity. To do so, we varied the number of patches from 3 to 100 to mimic the observed values, and varied the dispersal rates based on colonization rates of Dubart et al. [43], in which realized colonization γ of an empty patch is the product of the per-capita colonization coefficient and the surrounding occupancy (i.e., propagule pressure from neighbouring pools). Combining their reported values for these quantities implies effective annual among-patch coupling probabilities of order m~0.03. Converting this annual probability to a continuous-time daily rate gives $d=-\log(1-m)/365\approx 8.3\times 10^{-5}\;{day}^{-1}$, i.e., on the order of $10^{-4}\;{day}^{-1}$. We therefore varied *d* over the range $[10^{-6},\;10^{-4}]\;{day}^{-1}$. Because *Daphnia* rock-pool metacommunities are thought to be governed by preemptive competition, we varied the mean interspecific interaction strength $\overset{―}{a}$ between 1 and 1.4, which is equal to or larger than the intraspecific interactions (set to 1). We further chose an exponential dispersal kernel with characteristic distances varied across species between 0.1 and 0.5 and created a unit square landscape (as in the main simulations). This represents the fact that the typical cluster is several hundred meters wide while dispersal beyond 100m is considered unlikely, leading to localized dispersal. Within these system-specific model parameterization, we varied the coefficient of variation on species interactions (two levels), whether these interactions varied across patches (four levels), and whether there was regional equivalence of the growth rate (three levels).

We ran the model with *n* = 3 species, matching the regional presence of three focal *Daphnia* taxa in the dataset, and assessed coexistence across all patches. Then, we identified parameter combinations for which all three species coexisted in less than 1% of the patches, matching the rare coexistence of all three species in the empirical system. For these parameter combinations, we evaluated how dispersal and landscape size affected co-occurrence of species pairs (*n* = 2), matching our statistical co-occurrence analysis above.

### Computer code and data

Data and computer code are available online at https://doi.org/10.5281/zenodo.15311497 and https://github.com/fdelaend/occupancy. All code was written in the free software R [37], version 4.4.0 Puppy Cup, using the packages bayesplot, pracma, rootSolve, feasoverlap, geodist, gridExtra, brms, tidybayes, patchwork, reticulate, xtable, tidyverse, gridExtra, sf, ggspatial, cowplot, and grateful [35,36,45–59], all available from CRAN.

## Results

### Species persistence

We start by recalling the decomposition (Eq. 2) introduced in the Methods, which partitions the probability that a randomly chosen focal species *i* persists in a random patch *l* into two ecologically relevant cases: (i) persistence when the focal species would be excluded without dispersal, and (ii) persistence when it would have persisted without dispersal. Below we treat each case in turn. Throughout, subscripts 0 denote densities that species *i* would attain if there was no dispersal and thus patches were completely disconnected.

Focusing on the first part, we were able to express species *i*’s equilibrium density in a patch *l*, ${\hat{N}}_{i}^{\left(l\right)}$, solely as a function of equilibrium densities without dispersal (S1 Appendix):



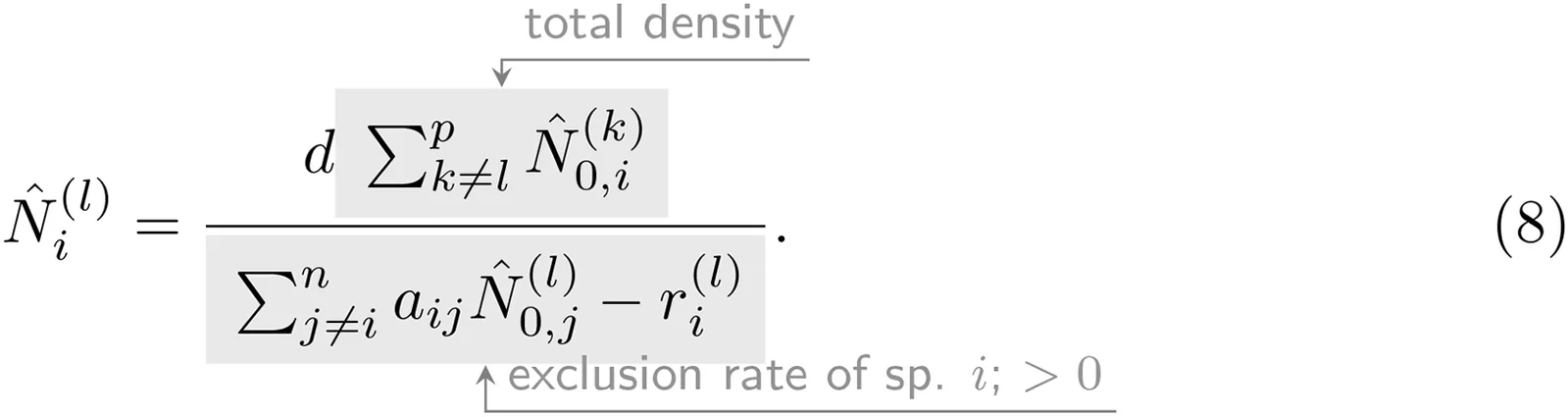



The numerator of Eq. 8 contains the sum of species *i*’s equilibrium densities in absence of dispersal, ${\hat{N}}_{0,i}^{\left(k\right)}$, across all patches except the focal patch. The denominator of Eq. 8 is the negative of the growth rate when species *i* invades patch *l* when a*ll* other species are at their equilibrium if there were no dispersal, ${\hat{N}}_{0,j}^{\left(l\right)}$. This latter growth rate is the invasion growth rate of coexistence theory [33]. The invasion growth rate quantifies the growth rate of a species following their introduction in an established community and must be positive for the species to persist. Given the case at hand (exclusion without dispersal), the invasion growth rate must be *negative*, which is why we coin its negative as the *exclusion rate* of species *i*. This means Eq. 8’s denominator is necessarily positive. Thus, we recover the result that a species is guaranteed to persist in all patches if dispersal is positive (*d* > 0) and when it would survive without dispersal in at least one other patch that is part of the spatial network. This conclusion is general: it does not depend on the exact parameter setting of Eq. 1 and therefore should hold for any number of species, and any species interaction scheme or landscape structure.

In realistic settings, having a positive density is insufficient for long-term persistence: low densities increase the risk of stochastic extinction. Thus, in many real-world applications, ${\hat{N}}_{i}^{\left(l\right)}$ needs to be larger than some extinction threshold θ. Eq. 8 shows that ${\hat{N}}_{i}^{\left(l\right)}$ is larger when the focal species is more abundant across the spatial network of disconnected patches (has greater $\sum_{k\neq l}^{p}{\hat{N}}_{0,i}^{\left(k\right)}$), dispersal is larger (*d* is larger), and the species gets excluded more slowly. This result quantifies how species persistence emerges as the net result of the total density across patches and local dynamics, both assessed in the case patches are disconnected. Importantly, more challenging local interactions can be compensated by adding patches, i.e., increasing landscape size. The added individuals come from the p−1 other patches, each of which donates a fraction ∝d of its equilibrium density to the focal patch.

By making strong assumptions on the distribution of species growth rates across patches (regional equivalence, see Methods) and species interactions (diffuse interactions that are weaker than intraspecific interactions, see Methods), we can calculate exactly how the numerator of Eq. 8 depends on interaction strength and landscape size (the number of patches *p*, Fig 2A), and how the denominator of Eq. 8 depends on the interaction strength *a* (Fig 2B) (details in S1 Appendix). Briefly, the total density across patches, in the *numerator* of Eq. 8, is identical for all species:


$$\begin{array}{c} \sum_{k\neq l}^{p}{\hat{N}}_{0,i}^{\left(k\right)}=\frac{1}{n}\sum_{m=1}^{n}f(m)(p-1)\frac{m{\overset{―}{r}}_{m}}{1+a(m-1)}\;\forall \;i, \end{array}$$ (9)

where *m* is the number of locally persisting species without dispersal, which happens with probability *f*(*m*), and ${\overset{―}{r}}_{m}$ is the mean growth rate across those species. This last growth rate does not have a simple closed form. In S1 Appendix, we show how to approximate this mean growth rate, and that it matches simulated values (Fig B in S1 Appendix). The *denominator* of Eq. 8 is a random variable that varies across patches and species. It depends on the spatial distribution of the total local density when there is no dispersal, which in turn depends on *f*(*m*), and the distribution of the focal species’ growth rate.

**Fig 2:**
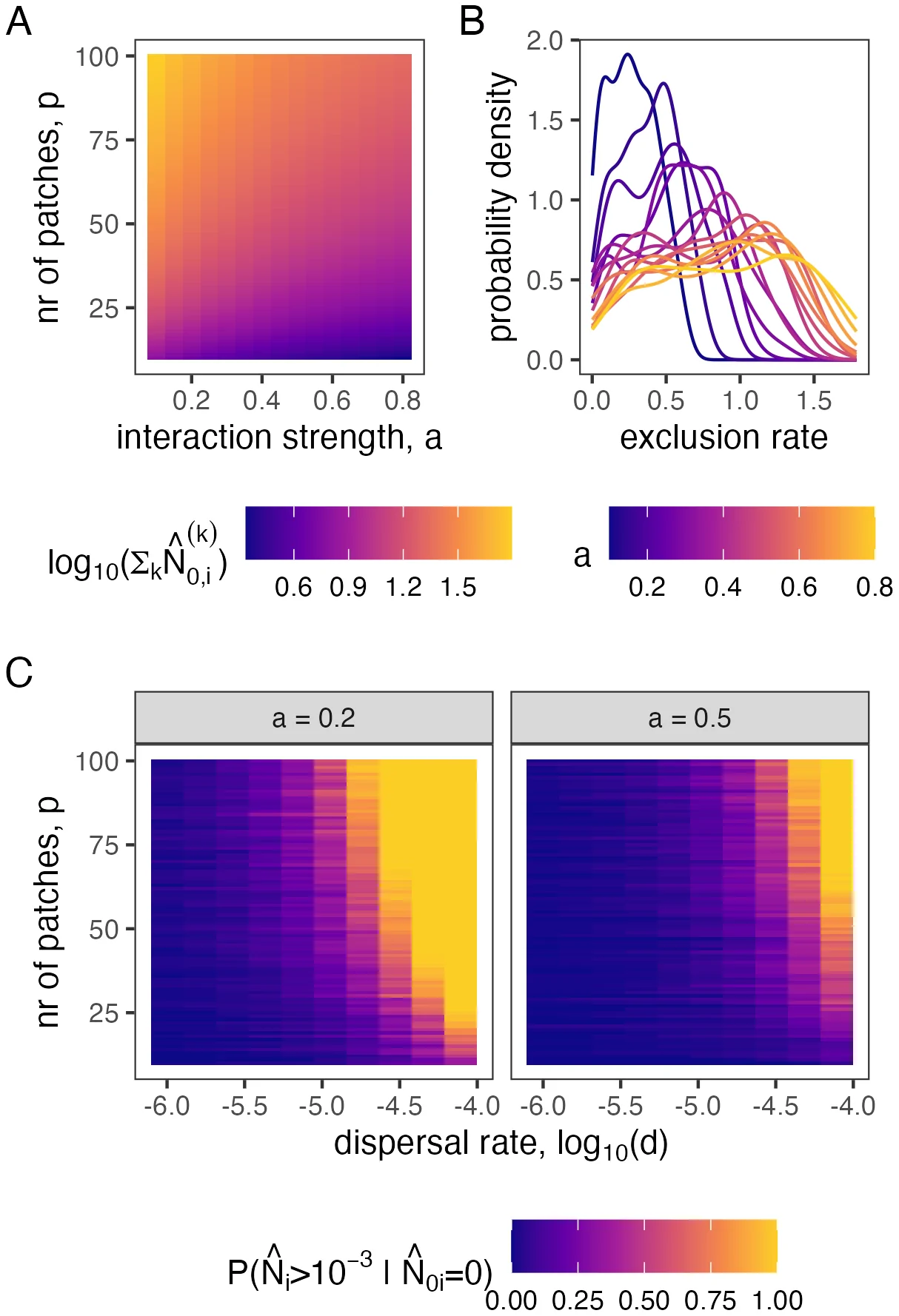
Landscape size and interaction strength predict species persistence (${\hat{N}}_{i}^{\left(l\right)}>10^{-3}$) in case exclusion would happen without dispersal (${\hat{N}}_{0,i}^{\left(l\right)}=0$). All values are analytical predictions from Eq. 8, assuming that interspecific competition is diffuse and weaker than intraspecific competition ($a_{ij}=a<a_{ii}=1$) and assuming regional equivalence (all species have the same regional mean growth rate). Growth rates $r_{i}^{\left(l\right)}$ are drawn i.i.d. from a truncated-normal distribution on ${\mathbb{R}}^{+}$, rescaled so that both the across-species mean within a patch and the across-patch mean for any species are equal to 1. ${\hat{N}}_{i}^{\left(l\right)}$ and ${\hat{N}}_{0,i}^{\left(l\right)}$ are the equilibrium densities of species *i* in patch *l* with and without dispersal, respectively. **(A)** A species’ total density across all patches is fixed, identical for all species, and depends only on *a* and the number of patches ***p*. (B)** Species’ exclusion rates vary with ***a*. (C)** Probability that ${\hat{N}}_{i}^{\left(l\right)}$ exceeds the extinction threshold (10^–3^), for *n* = 10 species.

Combining both parts of Eq. 8 shows how local persistence of a focal species depends on landscape size, dispersal rate, and local interaction strength (Fig 2C). Persistence is more likely in larger and better-connected networks, and when interactions are weaker, the total density across patches is higher, and exclusion is slower, respectively. The resulting probability that a focal species in the exclusion case exceeds the extinction threshold reads


$$\begin{array}{c} P\;\left({\hat{N}}_{i}^{\left(l\right)}>\theta \;|\;{\hat{N}}_{0,i}^{\left(l\right)}=0\right)=P\;\left(\frac{d\sum_{k\neq l}^{p}{\hat{N}}_{0,i}^{\left(k\right)}}{a\sum_{j\neq i}^{n}{\hat{N}}_{0,j}^{\left(l\right)}-r_{i}^{\left(l\right)}}>\theta \right), \end{array}$$ (10)

where the probability is taken conditional on $r_{i}^{\left(l\right)}<a\sum_{j\neq i}^{n}{\hat{N}}_{0,j}^{\left(l\right)}$ (i.e., the focal species being locally excluded without dispersal).

We finally focus on the second case of Eq. 2, dealing with persistence of species that persist without dispersal. Under regional equivalence and diffuse interspecific interactions that are weaker than intraspecific ones (*a* < 1), the first-order correction ${\hat{N}}_{1,i}^{\left(l\right)}$ to the focal species’ equilibrium density takes the form (S1 Appendix):


$$\begin{array}{c} {\hat{N}}_{1,i}^{\left(l\right)}=\frac{a\left((1-a){\overset{―}{N}}_{1}^{\left(l\right)}(n-m)+(m-1)\;\overset{―}{\rho }+(2-m)\;\rho_{i}\right)+(p-1)(1-a)-\rho_{i}}{\left(1-a)\;(a(m-1)+1\right)}, \end{array}$$ (11)

where $\rho_{i}\equiv \sum_{k\neq l}^{p}{\hat{N}}_{0,i}^{\left(k\right)}/{\hat{N}}_{0,i}^{\left(l\right)}$ is the focal species’ regional-to-local density ratio, $\overset{―}{\rho }$ is the mean of $\rho_{j}$ across the other locally persisting species, and ${\overset{―}{N}}_{1}^{\left(l\right)}$ is the mean first-order correction across the species that do *not* persist locally without dispersal. In the S1 Appendix, we show that this correction is positive for two limiting cases (a→1, with *n*>> *p*, and a→0). Numerical evaluation across the parameter range of *a* explored here confirms this conclusion (Fig C in S1 Appendix): the first-order correction term is nearly always positive, and so whenever a species persists without dispersal, it almost certainly persists with dispersal as well, $P({\hat{N}}_{i}^{\left(l\right)}>0|{\hat{N}}_{0,i}^{\left(l\right)}>0)\approx 1$.

### Coexistence: Community-level patch occupancy

We can now use our species-level results to probe community-level patch occupancy, building on the community-level decomposition (Eq. 3) introduced in the Methods. For the first case (no coexistence without dispersal), our species-level results imply that the *m* species that do persist without dispersal will persist with dispersal as well. We can therefore focus on the n−m species that do not persist without dispersal. We approximate the probability for all of these to persist as the (n−m)th power of $P({\hat{N}}_{i}^{\left(l\right)}>\theta |{\hat{N}}_{0,i}^{\left(l\right)}=0)$, where θ is again a small extinction threshold and ${\hat{N}}_{i}^{\left(l\right)}$ is given by Eq. 8. Denoting the distribution across the patches of the number of persisting species again as *f*(*m*), we write:


$$\begin{array}{c} P({\hat{N}}_{i}^{\left(l\right)}>\theta ,\;\forall i|\;\exists i:{\hat{N}}_{0,i}^{\left(l\right)}=0)=\sum_{m=1}^{n-1}f(m)P^{n-m}({\hat{N}}_{i}^{\left(l\right)}>\theta |{\hat{N}}_{0,i}^{\left(l\right)}=0). \end{array}$$ (12)

For the second case (coexistence without dispersal), our species-level results imply that $P({\hat{N}}_{i}^{\left(l\right)}>\theta ,\;\forall i|{\hat{N}}_{0,i}^{\left(l\right)}>\theta ,\;\forall i)\approx 1$. Overall, we obtain the final result for patch occupancy:


$$\begin{array}{c} P({\hat{N}}_{i}^{\left(l\right)}>\theta ,\;\forall i)=\left[\sum_{m=1}^{n-1}f(m)\left(P^{n-m}({\hat{N}}_{i}^{\left(l\right)}>\theta |{\hat{N}}_{0,i}^{\left(l\right)}=0)\right)\right]\cdot \left[1-\Xi (n)\right]+\Xi (n), \end{array}$$ (13)

where we have abbreviated $P({\hat{N}}_{0,i}^{\left(l\right)}>\theta ,\;\forall i)$ as Ξ(n), the feasibility-domain size introduced in the Methods. For the case of regional equivalence and diffuse interactions that are weaker than intraspecific interactions, we can compute patch occupancy analytically using Eq. 13 and the methods explained in S1 Appendix. The calculated values compare well with simulations of Eq. 1 that respect the two assumptions our analyses are based on. Specifically, patch occupancy sharply increases with the dispersal rate *d*, and this happens at lower *d* when interactions are weaker or the spatial network is larger (Fig 3A).

**Fig 3:**
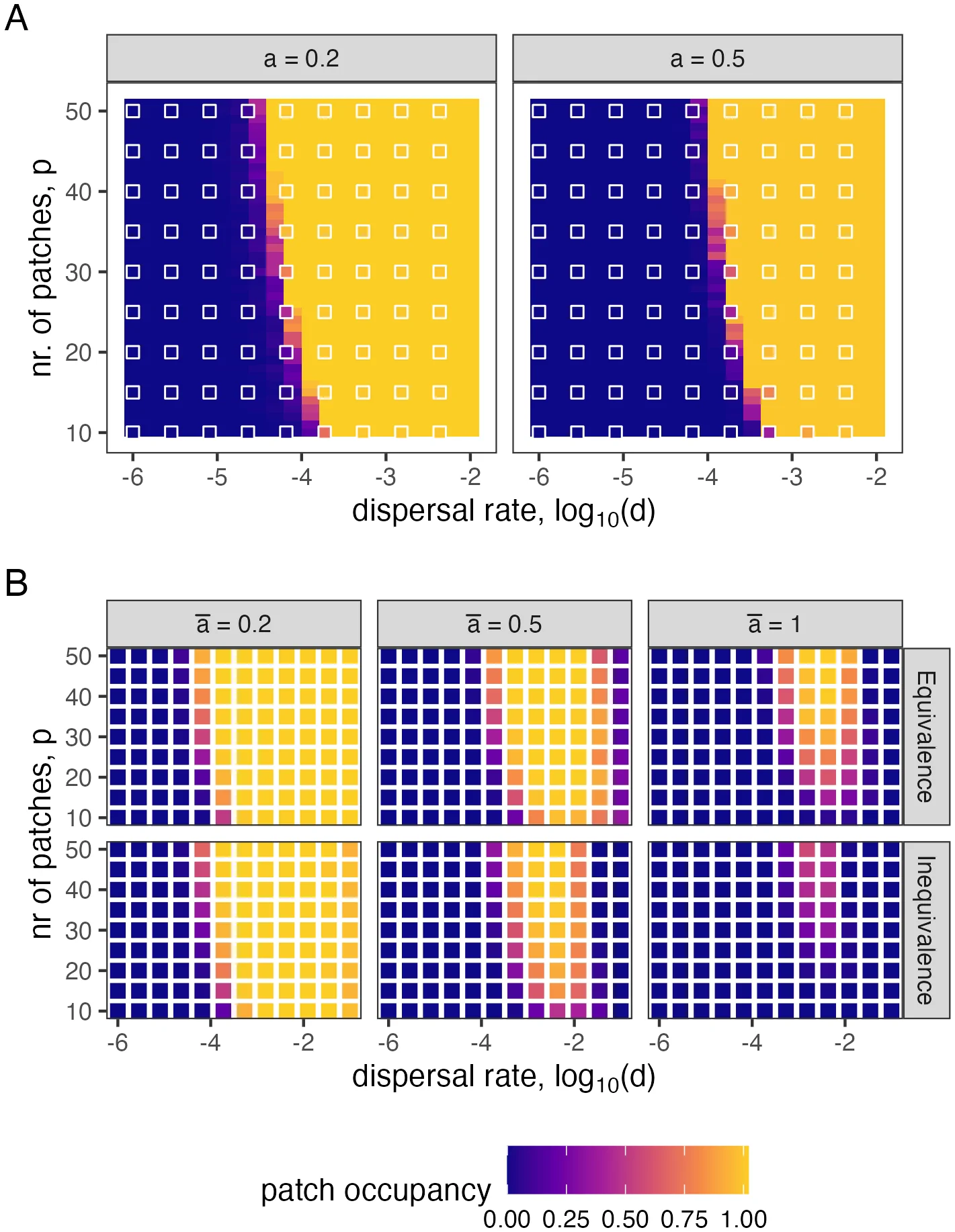
**A: Predicted (background color) and simulated (filled squares, overplotting the heat map in the background) community-level patch occupancy (fraction of patches in which all regionally available species persist) as a function of the dimensionless dispersal rate *d* and landscape size *p*.**Simulations are numerical integrations of Eq. 1 with *n* = 10 species, and respecting the two assumptions of our analyses. The simulation protocol is in Section Simulations. Background colors are analytical predictions for *n* = 10 by Eq. 13. Panels correspond to two values of the interspecific interaction strength *a*, which is the same across all species pairs (diffuse interactions) and always weaker than intraspecific interactions (all set to $a_{ii}=1$). Note that numerical exploration suggests values of *d* up to ≈0.005 are weak in this model (Fig D in S1 Appendix). **B: Weak dispersal and landscape size promote community-level patch occupancy, even when the assumptions of our analyses are relaxed.** Filled squares show simulated patch occupancy (fraction of patches where all *n* = 10 species coexist) as a function of the mean dimensionless dispersal rate *d* and the number of patches ***p*.** Interactions vary spatially and are no longer diffuse: they vary across species pairs within a patch with coefficient of variation $\text{CV}(a_{ij})=0.2$. Dispersal follows an explicit exponential kernel with characteristic distances for species evenly spaced between 0.5 and 4 (inter-patch distances vary between about 10^–5^ and 1.4). Rows correspond to regional equivalence (all species have the same regional mean growth rate; upper row) or regional inequivalence (one species has a 1.5-fold larger, and the others a proportionally smaller, regional mean growth rate; bottom row). Columns correspond to values of $\overset{―}{a}$. Note that patch occupancy declines when dispersal is too fast (*d* > 0.01) and interactions are strong ($\overset{―}{a}=0.5$ or 1). Simulations are numerical integrations of the extended model, S1 Appendix, with *n* = 10 species, relaxing all assumptions. The simulation protocol is in Section Simulations.

Our model has implicit assumptions and our analytical predictions assume regional equivalence and that competition is diffuse and weaker than intraspecific competition. Finally, our results only use equilibrium population densities to determine coexistence, but the stability of these equilibria also matters for long-term coexistence. We therefore ran dynamical simulations where we randomly seeded species across patches and relaxed all assumptions. Specifically, we allowed interactions to vary across patches and across species pairs within a patch (abandoning diffuse interactions), considered various mean interaction strengths (including preemptive competition), allowed a single species to have a higher regional mean intrinsic growth rate than the others (breaking regional equivalence of the growth rate), and allowed dispersal to occur according to explicit dispersal kernels. Adding all these complexities did not qualitatively change the results: weak dispersal always promotes coexistence, and more so in larger landscapes (Fig 3B). Only when dispersal becomes sufficiently strong (corresponding to *d* > 0.005), i.e., strong enough that the linear approximation underlying our analysis no longer holds (Fig D in S1 Appendix), and for strong interspecific competition ($\overset{―}{a}=0.5$ or 1), does the effect of dispersal on patch occupancy become unimodal (Fig 3B). Under these conditions, the effect of landscape size, if any, is consistently positive. It is worth pointing out that “strong” dispersal in the mathematical sense used here is not necessarily ecologically strong. Because *r* is normalized to unity by construction, *d* measures the fraction of individuals dispersing per local per-capita generation time.

### *Daphnia* co-occurrence in rockpools

We now turn to the empirical test of our theoretical predictions, using the long-term *Daphnia* dataset described in the Methods. Two questions are addressed: (i) is there evidence for preemptive competition?, and (ii) does the probability of pair co-occurrence depend on local landscape size and on the desiccation rate (our proxy for dispersal frequency)?

For all three species, we found that if it was present alone in a rockpool in spring, it was very likely present alone in that same rockpool in summer as well (probability of about 90%, Fig 4; Table A in S1 Appendix). Specifically, the regression model (Eq. 4) showed that having the focal species as the only species in a pool in spring made it about 1000 times more likely to have that same species present as the only species in summer too, consistent with preemptive competition (log odds ratio increases by about 7; Fig 4). Fig E in S1 Appendix shows that predictions matched observed response-variable distributions.

**Fig 4:**
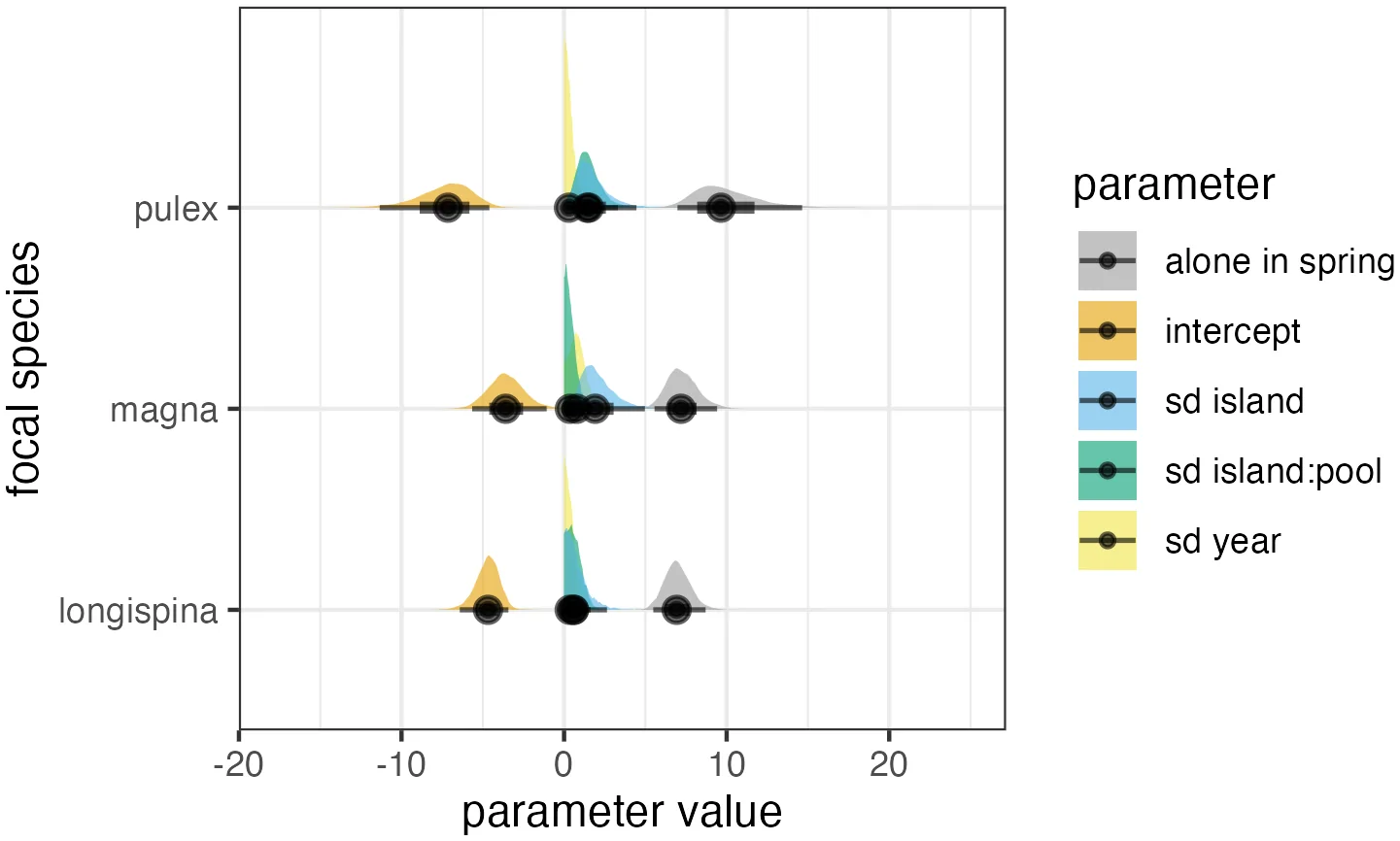
Evidence for preemptive competition across three *Daphnia* species in Tvärminne rock pools. Posterior distributions of the fixed-effect coefficients (of having the focal species as the sole species in spring) and of the random-effect standard deviations (island, pool within island, year) from Bayesian hierarchical binomial GLMMs (Eq. 4) fitted separately for each focal species. A positive coefficient for all three species indicates preemptive competition: when a species occupies a pool alone in spring, it is likely to be found alone in the same pool in the subsequent summer. Dots are posterior means; thick and thin intervals are the 66% and 95% credible intervals, respectively. A negative intercept corresponds to a baseline log-odds < 0, i.e., a probability < 0.5 of the focal species being the sole occupant in summer when it was not the sole occupant in spring. Numerical values are in Table A in S1 Appendix.

Co-occurrence of two *Daphnia* species was rare in pools surrounded by fewer than 10 other pools: less than 10% of such pools had species pairs. However, this percentage increased with the number of surrounding pools, regardless of the radius considered (Figs 5 and 6). This effect was consistent between sampling times (spring or summer). In contrast, the desiccation rate (our proxy for dispersal frequency) did not significantly influence co-occurrence (Fig 5). Fig F in S1 Appendix confirms that the posterior predictive distribution matches the observed response-variable distribution well. Detailed results are reported in Table B in S1 Appendix.

**Fig 5:**
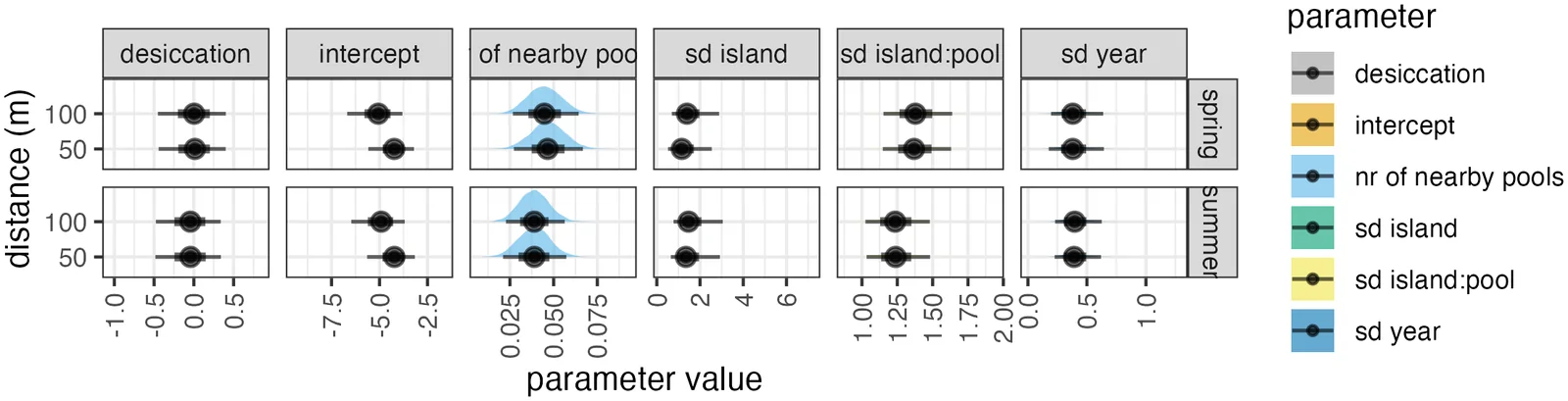
Occurrence probability increases with local connectivity, while desiccation has no effects. Posterior distributions of fixed-effect coefficients and random-effect standard deviations from Bayesian hierarchical models fitted separately for spring and summer and at two spatial scales (50 m and 100 m). The effect of the number of nearby pools is consistently positive across seasons and distances, whereas desiccation effects are centered near zero. Dots are posterior means; thick and thin intervals are the 66% and 95% credible intervals, respectively; distributions illustrate posterior uncertainty in both fixed and random effects. Posterior distributions of the fixed effects are tabulated in Table B in S1 Appendix.

**Fig 6:**
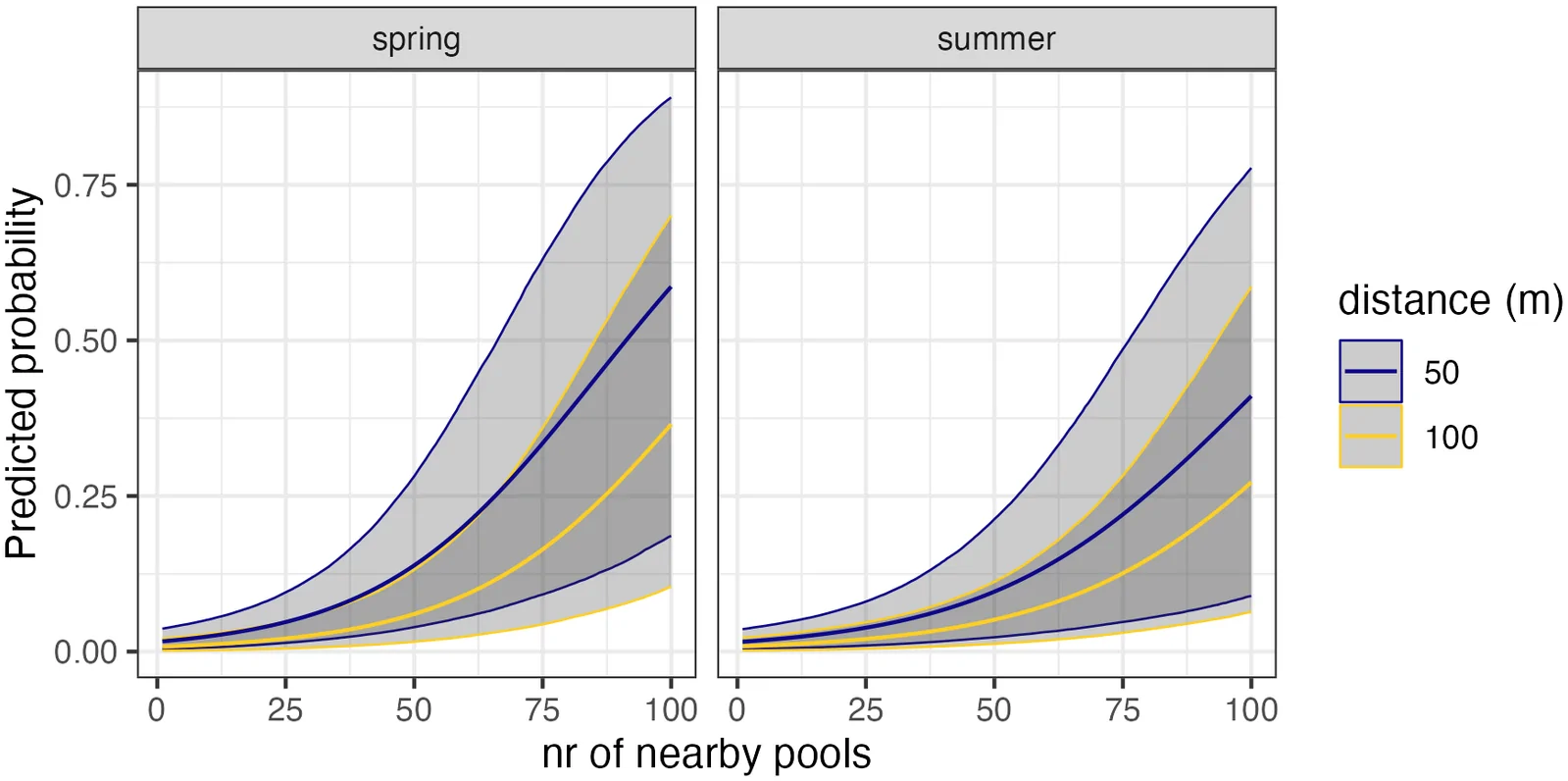
The number of surrounding pools increases the occurrence probability of *Daphnia* species pairs. Predicted probability of pair co-occurrence in a pool (response: 1 if two of the three *Daphnia* species were present; 0 if a single species was present) as a function of the number of neighbouring pools within a radius *b*, shown separately for spring and summer and for two spatial scales (*b* = 50 m and 100 m). Predictions are from a Bayesian binomial GLMM (Eq. 6) with fixed effects “number of nearby pools” and “desiccation rate” and random intercepts for island, pool nested within island, and year. Lines represent posterior mean predictions; shaded ribbons are 95% credible intervals.

We next simulated the effect of dispersal and number of patches with the extended model (S1 Appendix), parameterized to reflect the empirical system. Across all parameter combinations that reproduced the rare three-species coexistence observed in the empirical system, the number of patches had a consistently positive effect on the patch occupancy of species pairs (Fig 7). This pattern held regardless of whether growth rates were regionally equivalent or inequivalent, whether interactions varied little or substantially across species (cv(*a*) = 0.01 vs. 0.2), and across the full range of mean interspecific interaction strengths $\overset{―}{a}$ tested. As expected, higher mean interspecific competition suppressed pairwise co-occurrence relative to weaker competition, and greater variation in interaction coefficients elevated co-occurrence.

**Fig 7:**
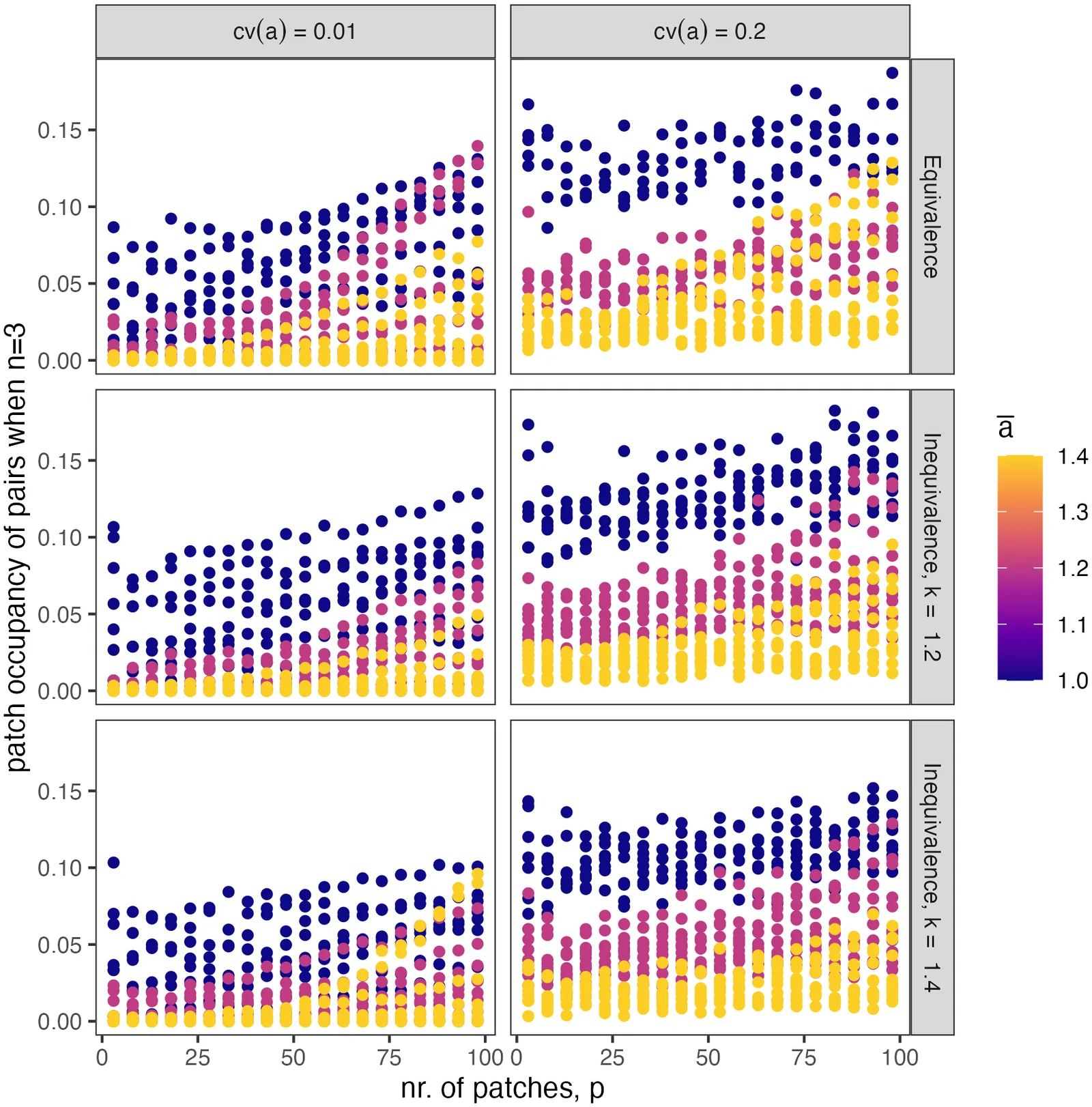
Landscape size consistently promotes pairwise species co-occurrence under system-specific parameterization. Patch occupancy of species pairs (fraction of patches occupied by exactly two of the three *Daphnia* species) as a function of the number of patches *p*, for parameter combinations in which full three-species coexistence is rare (< 1% of patches), matching the empirical system. Columns correspond to low and high variation in interspecific interaction coefficients (cv(*a*) = 0.01 and 0.2); rows correspond to regional equivalence or inequivalence of growth rates (inequivalence level *k* = 1.2 and 1.4). Colour encodes the mean interspecific interaction strength $\overset{―}{a}$. Each point represents a distinct parameter combination. Across all panels, the number of patches has a positive effect on pairwise co-occurrence, demonstrating the generality of the result under empirically motivated parameterization of the Tvärminne *Daphnia* metacommunity.

Within the range of dispersal rates that reproduced the observed rare coexistence of all three species ($d\in [10^{-6},10^{-4}]\;{day}^{-1}$), the effect of dispersal rate on pairwise co-occurrence was positive but weaker than the effect of the number of patches (Fig 8). This suggests that, within the range of dispersal rates consistent with the empirical system, the size of the metacommunity is a stronger driver of pairwise co-occurrence than the dispersal rate.

**Fig 8:**
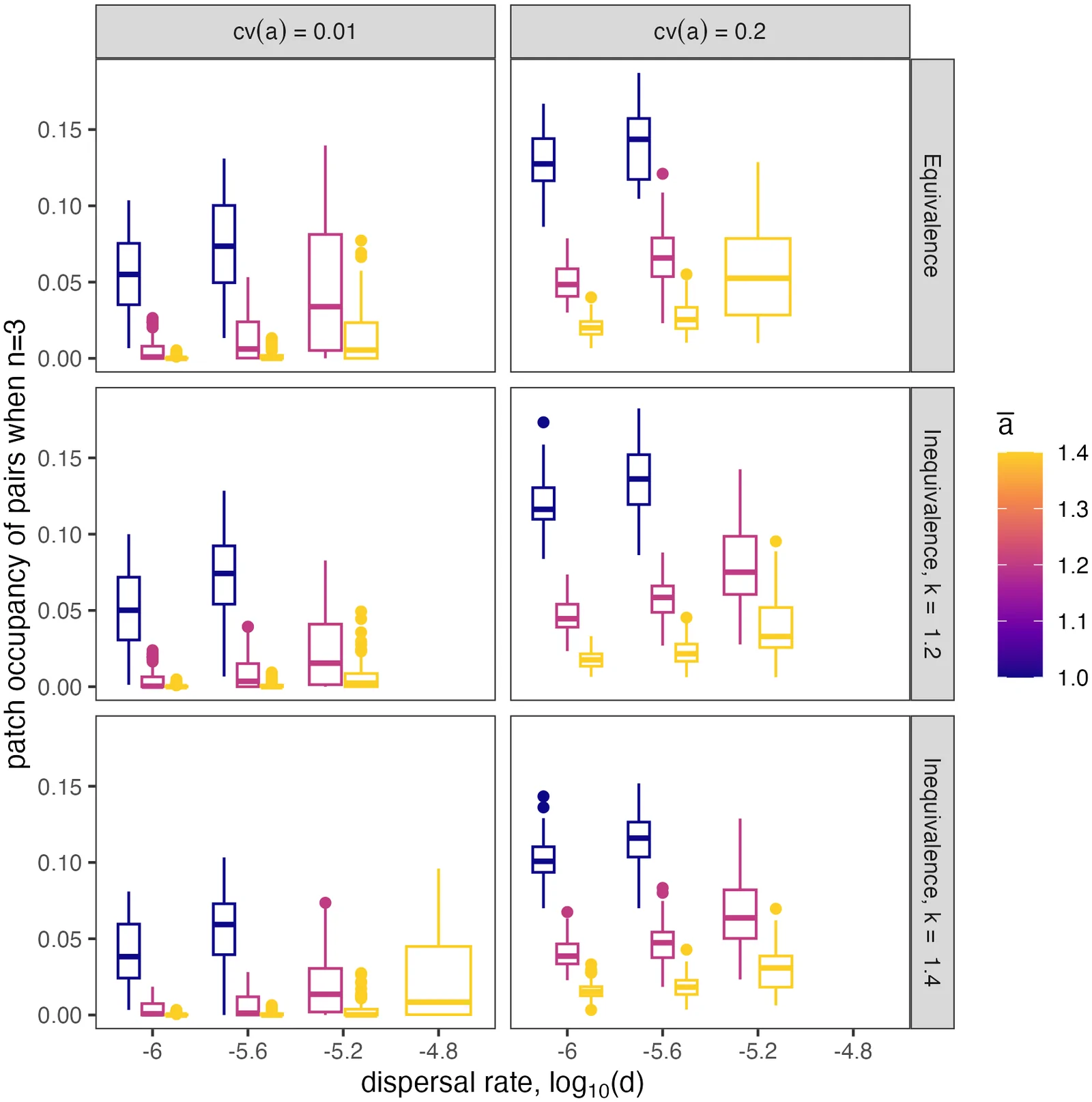
Dispersal rate has a weaker effect on pairwise co-occurrence than metacommunity size within the empirically constrained range. Patch occupancy of species pairs as a function of dispersal rate *d* (log_10_ scale), for the same parameter combinations as in Fig 7. Boxplots summarise the distribution across numbers of patches *p* and spatial variation of interactions; colours encode mean interspecific interaction strength $\overset{―}{a}$. Panel layout follows Fig 7. Note that at the highest dispersal rate ($\log_{10}(d)=-4.8$), some $\overset{―}{a}$ levels are absent because those parameter combinations no longer satisfy the criterion of rare three-species coexistence. Within the empirically motivated dispersal range, variation across dispersal levels is small relative to within-level variation driven by differences in the number of patches, reinforcing that metacommunity size is the dominant driver of pairwise co-occurrence. Simulations are numerical integrations of the extended model, see S1 Appendix, with *n* = 3 species. The simulation protocol is in Section *Daphnia* metacommunity.

## Discussion

We have presented theory and simulations showing that weak dispersal and landscape size (number of patches) inevitably promote local persistence and coexistence of competing species. Our analyses show how persistence emerges as a balance between local exclusion and the total density across patches of a species, and that both parts can be estimated by treating the patches as if they were disconnected. We show that the fraction of patches in which all regionally available species coexist, which we coin *community-level patch occupancy*, is tightly linked to the size of the feasibility domain [30,31], an important concept in coexistence theory. Finally, we analyse data of a microcrustacean metacommunity inhabiting rock pools at the coast of Finland, showing that species more often co-occur when they are surrounded by more patches (rock pools). Simulations with our metacommunity model, tailored to the empirical system, conform this conclusion.

Our results shed new light on classic findings on biodiversity maintenance in spatial networks, obtained with a variety of models and data collection methods. The equilibrium theory of island biogeography [60] and its heir, the neutral theory of biodiversity [61,62], both predict that local richness emerges as a balance between local extinction and immigration. Our results highlight how the *exclusion rate* (the negative of the invasion growth rate) drives local loss of species. Its magnitude can be driven either by unsuitable local conditions (abiotic, environmental filtering, low $r_{i}^{\left(l\right)}$[63],), or by intense interspecific competition (biotic, large $\sum_{j\neq i}a_{ij}N_{j}$). Our results suggest that immigration, when dispersal is weak, is a simple linear function of the size of the landscape: all else equal, total density across patches is smaller in smaller networks, which makes density in a focal patch to fall more readily below the threshold for extinction. This relation holds under the well-mixed assumption, in which new patches connect to all other existing patches. Explanations for the positive effect of landscape size on local richness often evoke a greater niche availability in larger networks [64]. Our results show that landscape size in and by itself can have an effect on species persistence, and therefore mean species richness, without greater niche availability: an additional patch increases the total density across patches for every species, which can be interpreted as each species being assigned a slightly larger realized niche. In spirit, this mechanism is the same as the one driving the species–area relationship in the equilibrium theory of island biogeography and its recent extensions, which states that smaller islands host fewer species because population densities are lower, making stochastic extinctions more likely [60,65].

Our results can also be interpreted in the context of more recent concepts in spatial population and community ecology. At the level of single metapopulations, a classic result is that a species can only persist when its metapopulation capacity λ exceeds a well-defined threshold value [25–28]. The metapopulation capacity is obtained (at least in simple cases) as the leading eigenvalue of the matrix $M^{\left(kl\right)}/E^{\left(k\right)}$, where *M*^(*kl*)^ encodes the dispersal rate from patch *l* to patch *k*, and *E*^(*k*)^ is the rate of extinction in patch *k*. All other things equal, adding more patches to a *l*andscape necessarily leads to a higher λ [26,28]. Thus, Eq. 8 reflects the same idea as the fact that λ must exceed a threshold value, with the dispersal matrix *M*^(*kl*)^ and extinction rates *E*^(*k*)^ playing the role of Eq. 8’s numerator and denominator, respectively. Our main result can therefore be interpreted as a community-level extension of the well-known criterion for metapopulation persistence. This goes beyond the earlier results of Häussler et al. [27] in that it applies to all communities described by Eq. 1, not just trophic metacommunities with strictly bottom-up interactions. More recently, Padmanabha et al. [66] have introduced the concept of local landscape-mediated fitness as the ratio between colonization and extinction at a focal patch, and show that persistence is more likely as this ratio increases. Again, this is conceptually similar to our results (Eq. 8).

At the level of multiple species, dispersal can generate mass effects, whereby the influx of individuals alters local population densities and thereby interacts with local population dynamics [16,67]. One particular manifestation is the rescue of locally non-viable populations: dispersal can maintain a species in a patch where it would otherwise go extinct. Such effects are typically associated with strong dispersal [20]. Our analyses show, however, that a species that does not persist in the absence of dispersal (i.e., has a negative invasion growth rate) becomes more likely to reach a viable density as dispersal or the number of patches increases (Eq. 8). Thus, our results describe a rescue effect as a particular case of mass-effect dynamics and show that such rescue need not require strong dispersal.

We compared these findings against data from a zooplankton metacommunity of *Daphnia* species. It is worth noting that our closed-form analytical results (Eqs. 8–13) assume diffuse interspecific interactions that are weaker than intraspecific interactions (*a* < 1; see Methods). Because interactions among *Daphnia* species are preemptive ($\overset{―}{a}\geq 1$; Fig 4), the Tvärminne metacommunity falls outside this analytically tractable regime. We therefore compare the empirical results with our simulations rather than directly with the closed-form predictions. Simulations parameterized to capture the preemptive competition and rare three-species coexistence observed in this system show that the positive effect of landscape size on patch occupancy persists when $\overset{―}{a}\geq 1$, whereas the effect of dispersal becomes weaker and more variable (Figs 7 and 8).

A calibration of a patch occupancy model to the same Tvärminne *Daphnia* dataset analysed here has shown that temperature affects rates of colonization and extinction, and often unequally so among species [68], confirming earlier studies on the same system [44,69]. Amid these changes in local species interactions and most likely deviations from regional equivalence, our analysis suggests that coexistence is more likely in pools that are surrounded by a greater number of neighbour pools, offering support for our theoretical result that coexistence in a patch is a numbers game despite changing the specific parameter settings of our model. We did not find effects of desiccation rate (our proxy for dispersal frequency) on co-occurrence. Our simulations with the extended model, parameterized to reflect preemptive competition and rare coexistence in the empirical system, reproduced this result.

Our prediction that weak dispersal and landscape size are bound to promote biodiversity is supported by studies on microbiome composition, finding that greater host density (i.e., a higher number of patches), or more intense social interactions promote alpha diversity of the microbiome. This has been found for density of rodent hosts [70], grooming relationships among baboon hosts [71], and relationships between spouses in humans [72].

While our analyses and simulations cover a wide range of ecological settings, several limitations suggest avenues for future work. First, our analytical results are strictly valid only where dispersal has a linear effect on local density (i.e., *d* < 0.005 in our setup; Fig D in S1 Appendix), such that total population size is essentially unchanged. Simulations relaxing this assumption (up to *d* = 1) show that the effect of landscape size remains robust, whereas the effect of dispersal on coexistence becomes unimodal when interactions are strongly preemptive; capturing this analytically would require models that explicitly couple local and spatial dynamics. Second, we gauge persistence and coexistence through feasibility alone, disregarding local asymptotic stability [73–75]. Because weak dispersal and more patches are both known to stabilize metacommunities, our conclusions on population density are unlikely to be overturned by stability considerations, except perhaps in communities with strong functional segregation, where dispersal can instead destabilize [76]. Our analytical treatment is also less general for species that persist without dispersal, requiring the additional assumptions of diffuse interactions and regional equivalence. Third, we approximate demographic stochasticity only through an extinction threshold, even though stochastic immigration is known to shape macroecological patterns in similar models [20,34]. Fourth, we focus on local persistence and coexistence; extending the framework to regional coexistence using regional proxies for interactions, growth, and dispersal is a natural next step [34,68,77]. Fifth, we assume dispersal is cost-free; relaxing this [78] would lower the total density across patches (the numerator of Eq. 8) and could offset the benefits of more patches or stronger dispersal, consistent with earlier work [79]. Finally, although we model heterogeneous landscapes, spatial heterogeneity in local densities can also arise in nominally homogeneous landscapes from variable interaction strengths alone [23]; combined with weak dispersal, this is likewise expected to promote local richness [3], in contrast to the destabilizing effect of dispersal reported for strictly homogeneous models [20,66].

Overall, our results support the inevitability of local persistence and coexistence of competing species in spatial networks, provided dispersal is weak. These results provide a stepping stone towards more complex scenarios of landscape structure, dispersal syndromes, and alternative species interaction schemes.

## Supporting information

S1 AppendixSupplementary Methods, Tables and Figures.Single PDF (S1_Appendix.pdf) containing: the full derivations of the small-dispersal approximation and of the species-level (Eq. 2) and community-level (Eq. 3) decompositions of persistence and coexistence probability; the explicit calculation of $P({\hat{N}}_{i}^{\left(l\right)}>\theta |{\hat{N}}_{0,i}^{\left(l\right)}=0)$ and $P({\hat{N}}_{i}^{\left(l\right)}>0|{\hat{N}}_{0,i}^{\left(l\right)}>0)$ under regional equivalence and diffuse interactions; the simulation protocol used to verify the analytical results; the extended simulation model used to relax the well-mixed and equal-characteristic-distance assumptions (including the patch-specific interaction matrix, the exponential dispersal kernel, and the dispersal rescaling); the Bayesian binomial GLMMs fitted to the Tvärminne *Daphnia* data; and Figs A–F and Tables A–B referenced in the main text.(PDF)
