# Supplementary material for: Weak dispersal and landscape size inevitably promote local biodiversity in heterogeneous metacommunities of competing species
Source: PLoS Comput Biol. 2026 Oct 16;22(10):e1014028. doi: 10.1371/journal.pcbi.1014028 (PMC13705597; doi:10.1371/journal.pcbi.1014028)
Supplement: Supplementary file 1 [file pcbi.1014028.s001.pdf]

Weak dispersal and landscape size inevitably promote local biodiversity in heterogeneous metacommunities of competing species: Supporting Information

De Laender et al.

Contents

1 Supplemental methods **3**

1.1 Weak dispersal approximation . . . 4

1.2 Analyses . . . 6

1.2.1 Case 1: Exclusion without dispersal . . . 7

1.2.2 Case 2: Persistence without dispersal . . . 14

1.3 Simulation model . . . 16

1.4 Simulations for the *Daphnia* rockpool metacommunity . . . 18

2 Supplemental tables **21**

2.1 Preemptive competition . . . 21

2.2 Co-occurrence . . . 23

3 Supplemental figures **24**

3.1 For methods . . . 24

3.2 For results . . . 25

1 Supplemental methods

Model and notation

Model. We consider n species occupying p patches, with local densities $N_i^{(l)}(t)$ ($i = 1, \dots, n$; $l = 1, \dots, p$) governed by the spatial Lotka–Volterra competition model:

$$\frac{\mathrm{d}N_i^{(l)}}{\mathrm{d}t} = N_i^{(l)} \left(r_i^{(l)} - a_{ii} N_i^{(l)} - \sum_{j \neq i}^{n} a_{ij} N_j^{(l)} \right) - (p-1) D \, N_i^{(l)} + D \sum_{k \neq l}^{p} N_i^{(k)}, \tag{S1}$$

where $r_i^{(l)}$ is the intrinsic growth rate of species i in patch l, a_{ii} and a_{ij} are intra- and interspecific competition coefficients, and D is a dispersal rate (assumed identical for all species and all patch pairs, i.e., well-mixed dispersal). Throughout, we set $a_{ii} = 1$ for all i.

Notation. We use the following notation throughout:

- m: number of species that locally coexist in a patch in the absence of dispersal ($1 \leq m \leq n$). Because of differences in local growth rates and competitive exclusion, m can be smaller than n.

- $f(m)$: probability (fraction of patches) that a randomly drawn patch contains exactly m coexisting species in the absence of dispersal.

- $\Xi(m)$: size of the feasibility domain for a sub-community of m species. The feasibility domain is the volume in growth-rate space in which all m species have strictly positive equilibrium densities, given the interaction matrix $\mathbf{A} = (a_{ij})$ [1, 2]. It shrinks with m, meaning that it is geometrically harder for large sub-communities to be feasible.

- $\overline{r}_m^{(l)}$: mean of the growth rates of the m species that persist in patch l without dispersal.

- $\overline{r}_{-m}^{(l)}$: the mean of the growth rates of the $n - m$ excluded species in patch l without dispersal.

- $\overline{r}$: regional mean growth rate (equal across species in case of regional equivalence).

- $\hat{N}_{0,i}^{(l)}$, $\hat{N}_{1,i}^{(l)}$: 0th- and 1st-order terms of the equilibrium density of species i in patch l when expanded in d (Eq. S5).

1.1 Weak dispersal approximation

Let us write the equilibrium condition of the main model in the main text by setting the time derivative on its left hand side to zero:

$$\hat{N}_i^{(l)} \left(r_i^{(l)} - \hat{N}_i^{(l)} - \sum_{j \neq i}^{n} a_{ij} \hat{N}_j^{(l)} - (p-1)D \right) + D \sum_{k \neq l}^{p} \hat{N}_i^k = 0. \tag{S2}$$

We make one further change: the terms containing a factor of D are multiplied and divided by a constant D_0 expressing unit dispersal. This leaves the expression unchanged, but allows one to write solutions in terms of D/D_0, which is a unitless parameter. Let us denote this fraction by d; then $D = D_0 d$. Eq. S2 can now be written as

$$\hat{N}_i^{(l)} \left(r_i^{(l)} - \hat{N}_i^{(l)} - \sum_{j \neq i}^{n} a_{ij} \hat{N}_j^{(l)} - (p-1)D_0 d \right) + D_0 d \sum_{k \neq l}^{p} \hat{N}_i^k = 0. \tag{S3}$$

The solution to Eq. S3 can always be written as a formal power series in d (which is why it was important to make it dimensionless):

$$\hat{N}_i^{(l)} = \hat{N}_{0,i}^{(l)} + d\hat{N}_{1,i}^{(l)} + d^2 \hat{N}_{2,i}^{(l)} + \ldots \tag{S4}$$

Assuming dispersal is weak, $0 \leq d \ll 1$, allows us to truncate the series to the linear term (neglecting $O(d^2)$):

$$\hat{N}_i^{(l)} \approx \hat{N}_{0,i}^{(l)} + d\hat{N}_{1,i}^{(l)}. \tag{S5}$$

The accuracy of this truncation is confirmed numerically against full simulations in Fig. D below, which shows that the effect of d on local biomass is approximately linear up to $d \approx 0.005$.

We substitute Eq. S5 into Eq. S3 up to that linear term:

$$\left(\hat{N}_{0,i}^{(l)} + d\hat{N}_{1,i}^{(l)}\right)\left(r_i^{(l)} - \left(\hat{N}_{0,i}^{(l)} + d\hat{N}_{1,i}^{(l)}\right) - \sum_{j\neq i}^{n} a_{ij}\left(\hat{N}_{0,j}^{(l)} + d\hat{N}_{1,j}^{(l)}\right) - (p-1)D_0 d\right) + D_0 d\sum_{k\neq l}^{p}\left(\hat{N}_{0,i}^{(k)} + d\hat{N}_{1,i}^{(k)}\right) = 0. \tag{S6}$$

Since d is a parameter and the equation must hold for any value it might take, this equation can only hold if the 0th-order terms and the 1st-order terms in d both individually sum to zero. Collecting the 0th-order terms first:

$$\hat{N}_{0,i}^{(l)}\left(r_i^{(l)} - \hat{N}_{0,i}^{(l)} - \sum_{j\neq i}^{n} a_{ij}\hat{N}_{0,j}^{(l)}\right) = 0, \tag{S7}$$

which is the usual Lotka–Volterra equilibrium condition in patch l.

Continuing with the 1st-order terms in d in Eq. S6:

$$\hat{N}_{0,i}^{(l)}\left(-d\hat{N}_{1,i}^{(l)} - \sum_{j\neq i}^{n} d a_{ij}\hat{N}_{1,j}^{(l)} - (p-1)D_0 d\right) + d\hat{N}_{1,i}^{(l)}\left(r_i^{(l)} - \hat{N}_{0,i}^{(l)} - \sum_{j\neq i}^{n} a_{ij}\hat{N}_{0,j}^{(l)}\right) + D_0 d\sum_{k\neq l}^{p}\hat{N}_{0,i}^{(k)} = 0. \tag{S8}$$

Dividing by d:

$$\hat{N}_{0,i}^{(l)}\left(-\hat{N}_{1,i}^{(l)} - \sum_{j\neq i}^{n} a_{ij}\hat{N}_{1,j}^{(l)} - (p-1)D_0\right) + \hat{N}_{1,i}^{(l)}\left(r_i^{(l)} - \hat{N}_{0,i}^{(l)} - \sum_{j\neq i}^{n} a_{ij}\hat{N}_{0,j}^{(l)}\right) + D_0\sum_{k\neq l}^{p}\hat{N}_{0,i}^{(k)} = 0. \tag{S9}$$

Rearranging and simplifying:

$$\hat{N}_{1,i}^{(l)}\left(r_i^{(l)} - 2\hat{N}_{0,i}^{(l)} - \sum_{j\neq i}^{n} a_{ij}\hat{N}_{0,j}^{(l)}\right) - \hat{N}_{0,i}^{(l)}\sum_{j\neq i}^{n} a_{ij}\hat{N}_{1,j}^{(l)} + D_0\left(\sum_{k\neq l}^{p}\hat{N}_{0,i}^{(k)} - (p-1)\hat{N}_{0,i}^{(l)}\right) = 0. \tag{S10}$$

This is a linear equation in the $\hat{N}_{1,i}^{(l)}$, assuming that the $\hat{N}_{0,j}^{(l)}$ have already been calculated and are known. In that case, the bracketed term above is the coefficient of $\hat{N}_{1,i}^{(l)}$ and $-a_{ij}\hat{N}_{0,i}^{(l)}$ are the coefficients of $\hat{N}_{1,j}^{(l)}$, $\forall\ j \neq i$, and the final term $D_0(\sum_{k\neq l}^{p}\hat{N}_{0,i}^{(k)} - (p-1)\hat{N}_{0,i}^{(l)})$ is the intercept. In what follows, we use Eq. S10 to analyze persistence of a focal species i in patch l for two

cases: either species i goes extinct when there is no dispersal ($\hat{N}_{0,i}^{(l)} = 0$), or it persists when there is no dispersal ($\hat{N}_{0,i}^{(l)} > 0$).

Note that we omit D_0 in the next sections and in the main text, since $D_0 = 1$ is a unit dispersal rate introduced just for being able to express a power series in the unitless parameter $d = D/D_0$.

1.2 Analyses

We define local species persistence probability as the fraction of patches in which a focal species i persists, which is equivalent to the probability that a random species persists in a random patch (i.e., the probability that $\hat{N}_i^{(l)} > 0$). We partition this probability into two ecologically relevant cases, using the law of total probability:

$$P(\hat{N}_i^{(l)} > 0) = \underbrace{P(\hat{N}_i^{(l)} > 0 | \hat{N}_{0,i}^{(l)} = 0) \cdot P(\hat{N}_{0,i}^{(l)} = 0)}_{\text{persistence, case exclusion w/o dispersal}} + \underbrace{P(\hat{N}_i^{(l)} > 0 | \hat{N}_{0,i}^{(l)} > 0) \cdot P(\hat{N}_{0,i}^{(l)} > 0)}_{\text{persistence, case persistence w/o dispersal}} . \tag{S11}$$

The first term describes persistence of species that would go extinct without dispersal ("rescue"); the second term describes persistence of species that already persist without dispersal.

The natural community-level analogue is the probability that all n regionally available species coexist in a randomly drawn patch, which we partition by the same logic:

$$\begin{aligned} P(\hat{N}_i^{(l)} > 0,\ \forall i) = & \overbrace{P(\hat{N}_i^{(l)} > 0,\ \forall i |\ \exists i : \hat{N}_{0,i}^{(l)} = 0) \cdot \Big(1 - P(\hat{N}_{0,i}^{(l)} > 0,\ \forall i)\Big)}^{\text{coexistence, if no coexistence w/o dispersal}} + \\ & \underbrace{P(\hat{N}_i^{(l)} > 0,\ \forall i | \hat{N}_{0,i}^{(l)} > 0,\ \forall i) \cdot P(\hat{N}_{0,i}^{(l)} > 0,\ \forall i)}_{\text{coexistence, if coexistence w/o dispersal}} . \end{aligned} \tag{S12}$$

where $P(\hat{N}_{0,i}^{(l)} > 0,\ \forall i)$ is the probability that all species have positive density in absence of dispersal.

To obtain explicit expressions for the conditional probabilities appearing in Eqs. S11 and S12, given spatial variation of the intrinsic growth rates $r_i^{(l)}$, we introduce the following simplifying assumptions:

- *Regional equivalence of growth rates.* For each species i, the patch-average $\frac{1}{p}\sum_l r_i^{(l)}$ is identical across species (and equal to 1 by our normalization). In other words, no species is intrinsically superior at the regional scale; differences between species arise only locally, because local growth rates $r_i^{(l)}$ vary across patches and species (illustrated in Fig. 1 of the main text.).

- *Diffuse interactions.* All interspecific coefficients are equal: $a_{ij} = a$ for all $i \neq j$, with $0 \leq a < 1$.

Given these assumptions, we can now calculate $\hat{N}_i^{(l)}$ for each of the two cases listed above.

1.2.1 Case 1: Exclusion without dispersal

Equilibrium density If the focal species i goes extinct in patch l when there is no dispersal, it implies that $\hat{N}_{0,i}^{(l)} = 0$. Eq. S10 then simplifies to:

$$\hat{N}_{1,i}^{(l)} \left(r_i^{(l)} - \sum_{j \neq i}^{n} a_{ij} \hat{N}_{0,j}^{(l)} \right) + \sum_{k \neq l}^{p} \hat{N}_{0,i}^{(k)} = 0, \tag{S13}$$

leading to:

$$\hat{N}_{1,i}^{(l)} = \frac{\sum_{k \neq l}^{p} \hat{N}_{0,i}^{(k)}}{\sum_{j \neq i}^{n} a_{ij} \hat{N}_{0,j}^{(l)} - r_i^{(l)}}, \tag{S14}$$

which implies that:

$$\begin{aligned} \hat{N}_i^{(l)} &\approx \hat{N}_{0,i}^{(l)} + d\hat{N}_{1,i}^{(l)} \\ &= 0 + \frac{d \sum_{k \neq l}^{p} \hat{N}_{0,i}^{(k)}}{\sum_{j \neq i}^{n} a_{ij} \hat{N}_{0,j}^{(l)} - r_i^{(l)}} \\ &= \frac{d \sum_{k \neq l}^{p} \hat{N}_{0,i}^{(k)}}{\sum_{j \neq i}^{n} a_{ij} \hat{N}_{0,j}^{(l)} - r_i^{(l)}}. \end{aligned} \tag{S15}$$

To obtain a closed-form expression for the right-hand side of Eq. S15, we now invoke the assumptions of *Regional equivalence of growth rates* and *Diffuse interactions.* Given these assumptions, we can calculate the two sums contained in Eq. S15: the total density of the

focal species i across all disconnected patches but the focal patch l ($\sum_{k\neq l}^{p} \hat{N}_{0,i}^{(k)}$), and the total density across all species within our focal patch l, in absence of dispersal ($\sum_{j\neq i}^{n} \hat{N}_{0,j}^{(l)}$). Under regional equivalence and diffuse interactions, the first of these sums is the same for all species. It therefore suffices to know the total density across all species and all patches but the focal one, which we define here as $\mathcal{N}_0$, and then divide this number by the number of species:

$$\sum_{k\neq l}^{p} \hat{N}_{0,i}^{(k)} = \frac{\mathcal{N}_0}{n} \ , \forall i \tag{S16}$$

To compute $\mathcal{N}_0$, we need to know (1) how many patches contain how many species, and (2) what the total density is of a patch when we know its number of species.

To address (1), we first define $f(m)$ as the fraction of patches that have exactly m locally coexisting species:

$$f(m) = \sum_{s} \xi(s) = \binom{n}{m} \Xi(m), \tag{S17}$$

where $m \leq n$ and $\xi(s)$ is the feasibility domain size for a specific sub-community $\mathcal{C}_s$ of m species ($|\mathcal{C}_s| = m \ \forall s$). The fact that all species pairs interact with the same strength a implies that $\xi(s)$ is the same for all $\mathcal{C}_s$ and only depends on m: the feasibility domain shrinks with m, all else equal [1]. We define $\Xi(m)$ as the function describing this dependence.

To address (2), the total density across all m species, where m can take any value between 1 and n, we first rewrite Eq. S7 as a matrix equation:

$$\hat{\mathbf{N}}_0 = \mathbf{A}^{-1}\mathbf{R}, \tag{S18}$$

where $\hat{\mathbf{N}}_0$ collects all equilibria, $\mathbf{A}^{-1}$ is the inverse of the interaction matrix, and $\mathbf{R}$ is the vector of growth rates. Given the diffuse interactions assumption, this interaction matrix $\mathbf{A}$ is

$$\mathbf{A} = \begin{pmatrix} 1 & a & \dots & a \\ a & 1 & \dots & a \\ \dots & \dots & \dots & \dots \\ a & a & \dots & 1 \end{pmatrix}, \tag{S19}$$

where diagonals are effects of species on themselves (taken to be $a_{ii} = 1,\ \forall i$), and off-diagonals are interspecific interaction coefficients $a_{ij} = a,\ \forall i, j$.

Because of the simple structure of $\mathbf{A}$, the Sherman-Morrison formula yields a particularly simple expression for $\mathbf{A}^{-1}$:

$$\mathbf{A}^{-1} = \mathbf{B}^{-1} - \frac{\mathbf{B}^{-1}\mathbf{u}\mathbf{v}^{\mathbf{T}}\mathbf{B}^{-1}}{1 + \mathbf{v}^{\mathbf{T}}\mathbf{B}^{-1}\mathbf{u}}, \tag{S20}$$

where

$$\begin{aligned} \mathbf{B} &= \begin{pmatrix} 1-a & 0 & \dots & 0 \\ 0 & 1-a & \dots & 0 \\ \dots & \dots & \dots & \dots \\ 0 & 0 & \dots & 1-a \end{pmatrix}, \\ \mathbf{u} &= \begin{pmatrix} \sqrt{a} & \sqrt{a} & \dots & \sqrt{a} \end{pmatrix}^{\mathbf{T}}, \\ \mathbf{v} &= \mathbf{u}^{\mathbf{T}}, \end{aligned} \tag{S21}$$

such that

$$\mathbf{B}^{-1} = \begin{pmatrix} 1/(1-a) & 0 & \dots & 0 \\ 0 & 1/(1-a) & \dots & 0 \\ \dots & \dots & \dots & \dots \\ 0 & 0 & \dots & 1/(1-a) \end{pmatrix},$$
$$\mathbf{B}^{-1}\mathbf{u}\mathbf{v}^{\mathbf{T}}\mathbf{B}^{-1} = \begin{pmatrix} a/(1-a)^2 & a/(1-a)^2 & \dots & a/(1-a)^2 \\ a/(1-a)^2 & a/(1-a)^2 & \dots & a/(1-a)^2 \\ \dots & \dots & \dots & \dots \\ a/(1-a)^2 & a/(1-a)^2 & \dots & a/(1-a)^2 \end{pmatrix}, \tag{S22}$$
$$1 + \mathbf{v}^{\mathbf{T}}\mathbf{B}^{-1}\mathbf{u} = 1 + ma/(1-a).$$

Therefore, the diagonal elements of $\mathbf{A}^{-1}$ are all identical; we denote them by α_{ii}:

$$\alpha_{ii} \equiv \left(\mathbf{A}^{-1}\right)_{ii} = -\frac{a(m-2)+1}{(a-1)(a(m-1)+1)} \quad \forall i, \tag{S23}$$

while the off-diagonals, again all identical, we denote α_{ij}:

$$\alpha_{ij} \equiv \left(\mathbf{A}^{-1}\right)_{ij} = \frac{a}{(a-1)(a(m-1)+1)} \quad \forall j \neq i. \tag{S24}$$

This means that $\hat{N}_{0,i}^{(l)}$ can now be computed by substituting Eqs. S23 and S24 into Eq. S18:

$$\begin{aligned} \hat{N}_{0,i}^{(l)} &= \alpha_{ii}\, r_i^{(l)} + \sum_{j\neq i}^{m} \alpha_{ij}\, r_j^{(l)} \\ &= -\frac{a(m-2)+1}{(a-1)(a(m-1)+1)} r_i^{(l)} + \frac{a}{(a-1)(a(m-1)+1)}(m-1)\overline{r}_{m-1}^{(l)} \\ &= \frac{a(m-1)\overline{r}_{m-1}^{(l)} - r_i^{(l)}(a(m-2)+1)}{(a-1)(a(m-1)+1)}, \end{aligned} \tag{S25}$$

which justifies the claim made above (after Eq. S16) that, under regional equivalence and diffuse interactions, the across-patch sum $\sum_{k\neq l}^{p} \hat{N}_{0,i}^{(k)}$ is the same for all species. Indeed, Eq. S25

expresses $\hat{N}_{0,i}^{(k)}$ as a linear function of $r_i^{(k)}$ and the local mean $\overline{r}_{m-1}^{(k)}$ of the other persisting species in patch k, with coefficients α_{ii}, α_{ij} that depend only on the local composition size m (not on species identity, by diffuse interactions). Summing over the $p-1$ non-focal patches gives

$$\sum_{k\neq l}^{p} \hat{N}_{0,i}^{(k)} = \sum_{k\neq l}^{p} \left[\alpha_{ii}(m)\, r_i^{(k)} + \alpha_{ij}(m)\,(m-1)\;\overline{r}_{m-1}^{(k)}\right]. \tag{S26}$$

By regional equivalence, the across-patch mean of $r_i^{(k)}$ is the same for every species ($=\overline{r}$); by diffuse interactions and the symmetry it imposes on the matrix $\mathbf{A}$, the local averages $\overline{r}_{m-1}^{(k)}$ are themselves species-symmetric. Both terms on the right of Eq. S26 therefore have the same value for every species i, which establishes Eq. S16.

Finally, summing Eq. S25 across all m species locally persisting in patch l gives the total local biomass $\sum_j \hat{N}_{0,j}^{(l)}$:

$$\begin{aligned}\sum_j \hat{N}_{0,j}^{(l)} &= \sum_j \frac{a(m-1)\overline{r}_{m-1}^{(l)} - r_j^{(l)}(a(m-2)+1)}{(a-1)(a(m-1)+1)}\\ &= \frac{m\,\overline{r}_m^{(l)}}{1+a(m-1)},\end{aligned} \tag{S27}$$

where $\overline{r}_{m-1}^{(l)}$ and $\overline{r}_m^{(l)}$ are the patch-specific mean growth rates of the $m-1$ and m species locally persisting in patch l, respectively. Because not all species persist in every patch (often, $m < n$), there will be a selection on $r_i^{(l)}$ [3]. More precisely, patches with $m < n$ tend to contain the m species with the highest growth rates.

To approximate $\overline{r}_m^{(l)}$, we first define the probability distribution $g^{(l)}$ of the $r_i^{(l)}$ across all species within a patch l, and denote its mean as $\overline{r}^{(l)}$. Specifically, we take $g^{(l)}$ to be a truncated normal distribution on $[0, \infty)$ with mean $\mu = 0$ and standard deviation $\sigma = 1$, rescaled so that the across-species mean within a patch and the across-patch mean for any species both equal 1. The lower truncation at zero ensures that growth rates are non-negative. By regional equivalence, $\overline{r}^{(l)}$ does not depend on species identity. If there is no dispersal, then for every species $i \notin \mathcal{C}$ (where $\mathcal{C}$ is again the composition in patch l, with $|\mathcal{C}| = m$, meaning $\mathcal{C}$ consists

of m elements; i.e., m species persist), it must hold that

$$r_i^{(l)} < a \sum_j \hat{N}_{0,j}^{(l)}, \tag{S28}$$

meaning i can't invade into patch l. This implies that the mean growth rate of the species not persisting without dispersal in patch l will be the mean of $g^{(l)}$, after truncation at $a \sum_j \hat{N}_{0,j}^{(l)}$. The distribution $g^{(l)}$ having a lower bound of zero, the mean of the excluded species, which we refer to here as $\overline{r}_{-m}^{(l)}$, will be located between zero and $a \sum_j \hat{N}_{0,j}^{(l)}$, i.e., $xa \sum_j \hat{N}_{0,j}^{(l)}$, with $0 \leq x \leq 1$. We then obtain:

$$\begin{aligned} \overline{r}_{-m}^{(l)} &\approx xa \sum_j N_{0,j}^{(l)} \\ &\approx \frac{xam\overline{r}_m^{(l)}}{1 + a(m-1)}, \end{aligned} \tag{S29}$$

where we use Eq. S27 to obtain $a \sum_j \hat{N}_{0,j}^{(l)}$ and define the mean growth rate of the m persisting species as $\overline{r}_m^{(l)}$. We can then substitute $\overline{r}_{-m}^{(l)}$ with $\frac{n\overline{r}^{(l)} - m\overline{r}_m^{(l)}}{n-m}$ and solve for $\overline{r}_m^{(l)}$, which leads to

$$\overline{r}_m^{(l)} \approx \frac{n\overline{r}^{(l)}(a(-m) + a - 1)}{m(am(x-1) - anx + a - 1)}. \tag{S30}$$

Since $\overline{r}^{(l)}$ is constant across patches (i.e., same for all l) and known by design, we can approximate $\overline{r}_m^{(l)}$ (and therefore also $\overline{r}_{-m}^{(l)}$) using only the model parameters a, m, and n, and the parameter x. The accuracy of this approximation will depend on how well the selected x allows estimating the mean of the truncated $g^{(l)}$. Setting $x = 0.5$ corresponds to a uniform distribution; here, we set $x = 0.45$ to account for the skew of $g^{(l)}$, which leads to predictions of $\overline{r}_m^{(l)}$ vs. m that match simulated values (values taken from the simulation protocol outlined in the methods, subsetting to $d = 10^{-6}$ to switch off dispersal) (Fig. B).

We then obtain $\mathcal{N}_0$ as the sum of the total densities across the $p-1$ patches with m species (called $\mathcal{N}_{0,m}$), for m varying from 1 to n:

$$\begin{aligned}\mathcal{N}_0 &= \sum_{m=1}^{n} \mathcal{N}_{0,m} \\ &= \sum_{m=1}^{n} f(m)\,(p-1)\,\frac{m\,\overline{r}_m^{(l)}}{1+a(m-1)},\end{aligned} \tag{S31}$$

where Eq. S30 gives $\overline{r}_m^{(l)}$. Dividing by n gives the total density across patches, per species (per Eq. S16):

$$\sum_{k\neq l}^{p} \hat{N}_{0,i}^{(k)} = \frac{1}{n}\sum_{m=1}^{n} f(m)\,(p-1)\,\frac{m\,\overline{r}_m^{(l)}}{1+a(m-1)}. \tag{S32}$$

Calculation of persistence probability To calculate $P(N_i^{(l)} > 0|\hat{N}_{0,i}^{(l)} = 0)$ we use Eq. S15. To calculate its numerator, $d\sum_{k\neq l}\hat{N}_{0,i}^{(k)}$, we use Eq. S32 and thus only need the model's basic parameters, and $f(m)$. Computing $f(m)$ involves computing the feasibility domain $\Xi(m)$ first (Eq. S17). To do so, one starts by taking the matrix of species interactions $\mathbf{A}$. For $m = n$, one can apply standard methods to get $\Xi(m)$. However, when $m < n$, we need to replace the columns of $\mathbf{A}$ that represent the absent species by unit vectors that have a -1 where the row number equals the column number [4]. Next, one can use this modified matrix to compute $\Xi(m)$ using the R package `feasoverlap` [2], constraining the feasibility domain on positive intrinsic growth rates.

To calculate Eq. S15's denominator, we sample 1000 values for m with probability $f(m)$. Given the sampled m, we then calculate the 1000 corresponding values for the mean growth rate of persisting species ($\overline{r}_m^{(l)}$, using Eq. S30) and total local density ($\sum_{j\neq i}^{n}\hat{N}_{0,j}^{(l)}$, using Eq. S27). We then sample $r_i^{(l)}$ such that $r_i^{(l)} < a\sum_{j\neq i}^{n}\hat{N}_{0,j}^{(l)}$ from the growth-rate distribution $g^{(l)}$, which is identical as the one used in the main-text simulation protocol, Section 1.3). We then evaluate $P(N_i^{(l)} > 0|\hat{N}_{0,i}^{(l)} = 0)$ as the fraction of resulting densities above the chosen extinction threshold (10^{-3}).

1.2.2 Case 2: Persistence without dispersal

Equilibrium density For species persisting without dispersal, the per capita growth rate at equilibrium is zero. Invoking the assumptions of *Regional equivalence of growth rates* and *Diffuse interactions*, we find:

$$
\begin{aligned}
0 &= \hat{N}_{1,i}^{(l)} \left(-\hat{N}_{0,i}^{(l)}\right) - a\hat{N}_{0,i}^{(l)} \sum_{j\neq i}^{n} \hat{N}_{1,j}^{(l)} + \sum_{k\neq l}^{p} \hat{N}_{0,i}^{(k)} - (p-1)\hat{N}_{0,i}^{(l)} \\
&= -\hat{N}_{1,i}^{(l)} - a\sum_{j\neq i}^{n} \hat{N}_{1,j}^{(l)} + \frac{\sum_{k\neq l}^{p} \hat{N}_{0,i}^{(k)}}{\hat{N}_{0,i}^{(l)}} - (p-1) \\
&= -\hat{N}_{1,i}^{(l)} - a\sum_{j\in\mathcal{C},j\neq i} \hat{N}_{1,j}^{(l)} - a\sum_{j\notin\mathcal{C}} \hat{N}_{1,j}^{(l)} + \rho_i - (p-1) \\
&= -\hat{N}_{1,i}^{(l)} - a\sum_{j\in\mathcal{C},j\neq i} \hat{N}_{1,j}^{(l)} - a\,(n-m)\,\overline{N}_1^{(l)} + \rho_i - (p-1),
\end{aligned}
\tag{S33}
$$

where we obtained the second line by dividing all terms by $\hat{N}_{0,i}^{(l)}$, and define a variable $\rho_i = \sum_{k\neq l}^{p} \hat{N}_{0,i}^{(k)} / \hat{N}_{0,i}^{(l)}$ on the third line that quantifies the ratio between the total density of a species across all but the focal patch, and the local density of that species in the focal patch. Note that we expect that $E\left[\rho_i\right] \geq 0$. On the third line, we also split the sum $\sum_{j\neq i}^{n} \hat{N}_{1,j}^{(l)}$ in two parts: one part contains those species that persist without dispersal; the other part contains those that don't. Again, we introduce $\mathcal{C}$ as the community composition without dispersal in patch l (with $|\mathcal{C}| = m$, meaning m species persist without dispersal). On the fourth line, we define the mean $\overline{N}_1^{(l)}$ as the mean of all $\hat{N}_{1,j}^{(l)}$ taken across species that *do not* persist without dispersal in patch l. For reasons explained in Section 1.2.1, this mean cannot be negative.

Eq. S33 is a system of m linear equations in the $\hat{N}_{1,i}^{(l)}$. The matrix holding the coefficients of this linear system has -1 on its diagonal and $-a$ as off-diagonals. The intercepts are in a vector with elements $-a\,(n-m)\,\overline{N}_1^{(l)} + \rho_i - (p-1)$. Because of the simple coefficient matrix, the inverse of the coefficient matrix can be obtained analogously to $\mathbf{A}^{-1}$ in Section 1.2.1: its diagonal entries equal $-\alpha_{ii}$ (i.e., $\frac{a(m-2)+1}{(a-1)(a(m-1)+1)}$) and its off-diagonal entries equal $-\alpha_{ij}$ (i.e.,

$-\frac{a}{(a-1)(a(m-1)+1)}$). The solution to this system is therefore

$$
\begin{aligned}
\hat{N}_{1,i}^{(l)} =& \left(\frac{a(m-2)+1}{(a-1)(a(m-1)+1)}\right)\left(-a\,(n-m)\,\overline{N}_1^{(l)} + \rho_i - (p-1)\right) \\
&+ \sum_{j\in\mathcal{C},j\neq i}\left(-\frac{a}{(a-1)(a(m-1)+1)}\right)\left(-a\,(n-m)\,\overline{N}_1^{(l)} + \rho_j - (p-1)\right) \\
=& \left(\frac{a(m-2)+1}{(a-1)(a(m-1)+1)}\right)\left(-a\,(n-m)\,\overline{N}_1^{(l)} + \rho_i - (p-1)\right) \\
&+ (m-1)\left(-\frac{a}{(a-1)(a(m-1)+1)}\right)\left(-a\,(n-m)\,\overline{N}_1^{(l)} + \overline{\rho} - (p-1)\right) \\
=& \frac{a\left((a-1)\overline{N}_1^{(l)}(n-m) - m\overline{\rho} + m\rho_i + \overline{\rho} + p - 2\rho_i - 1\right) - p + \rho_i + 1}{(a-1)(a(m-1)+1)} \\
=& \frac{a\left((1-a)\overline{N}_1^{(l)}(n-m) + (m-1)\overline{\rho} + (2-m)\rho_i\right) + (p-1)(1-a) - \rho_i}{(1-a)(a(m-1)+1)},
\end{aligned}
\tag{S34}
$$

where $\overline{\rho}$ must be nonnegative because the individual ρ_i must be as well.

This means that the equilibrium density with dispersal is:

$$
N_i^{(l)} \approx \underbrace{N_{0,i}^{(l)}}_{>0,\ \text{because persistence w/o dispersal}} + d\,\frac{a\left(\overbrace{(1-a)\overline{N}_1^{(l)}(n-m)}^{>0} + \overbrace{(m-1)\overline{\rho}}^{\geq 0} + (2-m)\rho_i\right) + \overbrace{(p-1)(1-a)}^{>0} - \overbrace{\rho_i}^{\geq 0}}{\underbrace{(1-a)(a(m-1)+1)}_{>0}},
\tag{S35}
$$

where annotations highlight terms in Eq. S35 that will always be positive, if interspecific interactions are weaker than intraspecific interactions ($a < 1$). The sign of $N_i^{(l)}$ will depend on the value of m and ρ_i, however. It is helpful to consider two limiting cases: one where interactions are very strong ($a \to 1$), and one where they are very weak ($a \to 0$). When interactions are very strong, every patch only contains a single species — the one with the highest growth rate $r_i^{(l)}$. If, in addition, $n \gg p$ (many more regionally available species than patches), then with high probability no species wins in more than one patch: with growth rates drawn i.i.d. across patches and species, the probability that the focal species i also has the largest $r_i^{(k)}$ in any other patch $k \neq l$ scales as $\sim (p-1)/n$ and vanishes for $n \gg p$. Under

this regime, the focal species is absent from all other patches whenever it wins patch l, making $\rho_i = 0$. This cancels out all terms with ρ_i in Eq. S35, leading to $N_i^{(l)} > 0$. When interactions are very weak, all species occur nearly everywhere, making $\rho_i \approx p-1$ and $m \approx n$, making Eq. S35:

$$\begin{aligned} N_i^{(l)} &= N_{0,i}^{(l)} + d\frac{a\left((n-1)(p-1) + (2-n)(p-1)\right) + (p-1)(1-a) - (p-1)}{(1-a)(a(n-1)+1)} \\ &= N_{0,i}^{(l)} + d\frac{a\,(p-1) + (p-1)(1-a) - (p-1)}{(1-a)(a(n-1)+1)} \\ &= N_{0,i}^{(l)}, \end{aligned} \tag{S36}$$

which confirms that $P(N_i^{(l)} > 0 | N_{0,i}^{(l)} > 0) \approx 1$: if a species persists without dispersal, it will almost certainly persist with dispersal as well.

Calculation of persistence probability We numerically calculated Eq. S34 using similar methods as in Section 1.2.1: sampling m from $f(m)$, calculating all the terms from S34, and finally calculating $N_i^{(l)}$ for all sampled values, and evaluating which fraction is greater than the chosen extinction threshold of 10^{-3}. These numerical calculations confirm that species persisting without dispersal will almost certainly persist with dispersal as well (Fig. C).

1.3 Simulation model

We tested how the analytical predictions degrade when the underlying assumptions are relaxed by running simulations to allow: (i) interactions to vary across species pairs and across patches (no diffuse, spatially-homogeneous interactions), and (ii) dispersal to occur through an explicit, species-specific dispersal kernel rather than a single rate shared by all species and all patch pairs. The corresponding system of $n \times p$ ODEs reads

$$\frac{\mathrm{d}N_i^{(l)}}{\mathrm{d}t} = N_i^{(l)}\left(r_i^{(l)} - a_{ii}^{(l)}N_i^{(l)} - \sum_{j\neq i}^{n} a_{ij}^{(l)}\,N_j^{(l)}\right) - N_i^{(l)}\sum_{k\neq l}^{p} d_{kl}^{(i)} + \sum_{k\neq l}^{p} d_{lk}^{(i)}N_i^{(k)}, \tag{S37}$$

where $a_{ij}^{(l)}$ is the per-patch interaction coefficient (with $a_{ii}^{(l)} = 1$ for all i, l) and $d_{lk}^{(i)}$ is the species-specific dispersal rate from patch k to patch l.

Simulation design. We set $n = 10$, and varied the number of patches p (10 to 50), the mean dispersal rate d (10^{-6} to 10^{-1}), the across-pairs mean $\overline{a}$ $(0.2, 0.5, 1)$ and variance of interspecific interaction strength (coefficients of variation of 0 or 0.2), whether these interactions varied across patches (yes or no), whether there was regional equivalence of the growth rate (yes or no), and if dispersal happened between all patch pairs (well-mixed scenario) or obeyed an exponentially declining dispersal kernel, whereby nearby patches exchange more individuals than farther ones. To break regional equivalence of the growth rate, we multiplied a randomly-picked species' regional mean growth rate by 1.5, and divided that of the other species by that same amount. For each parameter combination, we drew $r_i^{(l)}$ for each species i in each patch l independently from a truncated standard normal distribution on $[0, \infty)$. We started the simulations from random initial conditions $N_i^{(l)}(0) \sim \mathcal{U}(0, 1)$ until equilibrium (verified by checking that the maximum absolute time derivative was below 10^{-8}). Note that the parameter combination of weak dispersal rates $d < 10^{-2}$, the across-pairs mean $\overline{a} < 1$ and zero variance of interspecific interaction strength (coefficients of variation of 0), invariant interactions across patches, regional equivalence of the growth rates, and the well-mixed scenario, respects all implicit and explicit assumptions on which our analyses are based, and is therefore presented alongside the analytical predictions in Fig. 2 of the main text. Fig. 3 represents the case where these assumptions are violated (see caption of that figure for details).

Spatially-varying interactions. For each replicate we first drew a single base matrix $\mathbf{A}$ of dimension $n \times n$ with off-diagonal entries $\sim \mathcal{N}(\overline{a}, \sigma_a^2)$ (with $\sigma_a = \mathrm{CV}_a\,\overline{a}$) and unit diagonal, then made it symmetric. We obtained the patch-specific matrices $\mathbf{A}^{(l)}$ by element-wise multiplication of $\mathbf{A}$ with an i.i.d. matrix $\mathbf{V}^{(l)}$ whose entries are drawn from $\mathcal{U}(1 - v,\, 1 + v)$:

$$a_{ij}^{(l)} = a_{ij}\, V_{ij}^{(l)}, \qquad V_{ij}^{(l)} \sim \mathcal{U}(1 - v,\, 1 + v), \tag{S38}$$

where $v \in [0, 1]$ controls the magnitude of cross-patch variation in interaction strength. We set v to 0 or 0.2.

Explicit dispersal kernel. Patch positions $\mathbf{x}_l \in [0, 1]^2$ ($l = 1, \ldots, p$) were drawn independently from the uniform distribution on the unit square. Each species i was assigned a characteristic dispersal distance c_i, with the c_i taken to be evenly spaced on $[c_{\min}, c_{\max}] = [0.5, 4]$ across species (so that species span both shorter- and longer-ranged dispersal at the scale of the landscape). The unscaled species-specific dispersal rate from patch k to patch $l \neq k$ is then given by an exponential kernel of the inter-patch distance,

$$\tilde{d}_{lk}^{(i)} = \exp\left(-\frac{\|\mathbf{x}_l - \mathbf{x}_k\|}{c_i}\right), \qquad \tilde{d}_{ll}^{(i)} = 0, \tag{S39}$$

and we rescaled all non-zero entries of $\tilde{d}^{(i)}$ by a single multiplicative constant so that their mean equals the prescribed dispersal rate d (i.e., the mean of $\{d_{lk}^{(i)} : l \neq k\}$ across all species and patch pairs equals d):

$$d_{lk}^{(i)} = \frac{d}{\langle \tilde{d}^{(i)} \rangle_{\neq 0}} \, \tilde{d}_{lk}^{(i)}. \tag{S40}$$

This rescaling makes the explicit-kernel scenario directly comparable with the well-mixed scenario at the same average dispersal rate d.

Persistence threshold and replication. A species i was considered locally extinct in patch l if $\hat{N}_i^{(l)} < 10^{-3}$ at equilibrium. For each parameter combination we ran 100 replicate simulations with independently drawn $r_i^{(l)}$ matrices and reported the mean of the community-level patch occupancy, defined as the fraction of patches in which all n species coexist above the persistence threshold.

1.4 Simulations for the *Daphnia* rockpool metacommunity

We ran additional simulations with Eq. S37 to mimic the Tvärminne rock-pool *Daphnia* metacommunity. We varied the number of patches from 3 to 100 to mimic the observed values, and varied the dispersal rates based on colonization rates of Dubart et al. [5], in which realized

colonization γ of an empty patch is the product of the per-capita colonization coefficient and the surrounding occupancy (i.e., propagule pressure from neighbouring pools). Combining their reported values for these quantities implies effective annual among-patch coupling probabilities of order $c \sim 0.03$. Converting this annual probability to a continuous-time daily rate gives $d = -\log(1-c)/365 \approx 8.3 \times 10^{-5}\,\text{day}^{-1}$, i.e., on the order of $10^{-4}\,\text{day}^{-1}$. We therefore varied d over the range $[10^{-6}, 10^{-4}]\,\text{day}^{-1}$, which spans the lower-end and central empirical estimates (6 levels). Because *Daphnia* rock-pool metacommunities are thought to be governed by priority effects and the monopolization of pools by the first colonizing species or genotype [6, 7], we varied the mean interspecific interaction strength $\overline{a}$ between 1 and 1.4, which is equal to or larger than the intraspecific interactions (set to 1). We further chose an exponential dispersal kernel with characteristic distances varied across species between 0.1 and 0.5 and created a unit square landscape (as in the main simulations). This represents the fact that the typical cluster is several hundred meters wide while dispersal beyond 100m is considered unlikely, leading to localized dispersal. Within these system-specific model parameterization, we varied the coefficient of variation on species interactions (two levels), whether these interactions varied across patches (four levels), and whether there was regional equivalence of the growth rate (three levels).

We ran the model with $n = 3$ species, matching the presence of three focal *Daphnia* taxa in the dataset, and assessed coexistence across all patches. Then, we identified parameter combinations for which all three species coexisted in less than 1% of the patches, matching the rare coexistence of all three species in the empirical system. For these parameter combinations, we assessed coexistence of any two of the three species per patch, matching our statistical co-occurrence analysis.

Implementation. Simulations were implemented in R using the `deSolve` and `rootSolve` packages (steady states obtained via `rootSolve::runsteady` with absolute tolerance 10^{-12}, supplied with the analytic Jacobian of Eq. S37 for solver performance). A smooth cubic cutoff was applied to local densities below 10^{-10} to avoid numerical artefacts at very low abundances;

this affects only values orders of magnitude below the persistence threshold and does not alter the qualitative dynamics.

2 Supplemental tables

2.1 Preemptive competition

Table A: Estimated coefficients of the models (one per focal species), where *group* refers to the random effects.

effect	component	group	term	estimate	std.error	conf.low	conf.high	focal
fixed	cond		(Intercept)	-3.57	1.15	-5.75	-1.12	magna
fixed	cond		as.factormagna_spring1	7.28	0.94	5.62	9.32	magna
ran_pars	cond	island	sd___(Intercept)	2.11	1.10	0.63	4.94	magna
ran_pars	cond	island:pool	sd___(Intercept)	0.43	0.34	0.02	1.26	magna
ran_pars	cond	year	sd___(Intercept)	0.80	0.48	0.05	1.85	magna
fixed	cond		(Intercept)	-4.74	0.80	-6.53	-3.40	longispina
fixed	cond		as.factorlongispina_spring1	6.96	0.80	5.52	8.67	longispina
ran_pars	cond	island	sd___(Intercept)	0.82	0.77	0.03	2.79	longispina
ran_pars	cond	island:pool	sd___(Intercept)	0.65	0.45	0.03	1.68	longispina
ran_pars	cond	year	sd___(Intercept)	0.41	0.31	0.02	1.16	longispina
fixed	cond		(Intercept)	-7.40	1.70	-11.39	-4.67	pulex
fixed	cond		as.factorpulex_spring1	9.99	1.88	7.00	14.38	pulex
ran_pars	cond	island	sd___(Intercept)	1.76	1.11	0.30	4.56	pulex
ran_pars	cond	island:pool	sd___(Intercept)	1.53	0.73	0.32	3.20	pulex
ran_pars	cond	year	sd___(Intercept)	0.38	0.31	0.01	1.16	pulex

2.2 Co-occurrence

Table B: Estimated coefficients of the model for co-occurrence, where *group* refers to the random effects.

effect	component	group	term	estimate	std.error	conf.low	conf.high	sample	b
fixed	cond		(Intercept)	-4.28	0.60	-5.59	-3.21	spring	50.00
fixed	cond		nr_pools_within	0.05	0.01	0.03	0.07	spring	50.00
fixed	cond		desiccation_dynamic	0.00	0.21	-0.44	0.40	spring	50.00
ran_pars	cond	island	sd__(Intercept)	1.26	0.54	0.52	2.54	spring	50.00
ran_pars	cond	island:pool	sd__(Intercept)	1.37	0.12	1.15	1.63	spring	50.00
ran_pars	cond	year	sd__(Intercept)	0.39	0.12	0.18	0.64	spring	50.00
fixed	cond		(Intercept)	-5.11	0.74	-6.69	-3.81	spring	100.00
fixed	cond		nr_pools_within	0.04	0.01	0.03	0.06	spring	100.00
fixed	cond		desiccation_dynamic	-0.00	0.21	-0.45	0.40	spring	100.00
ran_pars	cond	island	sd__(Intercept)	1.50	0.57	0.70	2.88	spring	100.00
ran_pars	cond	island:pool	sd__(Intercept)	1.38	0.12	1.15	1.64	spring	100.00
ran_pars	cond	year	sd__(Intercept)	0.39	0.11	0.20	0.64	spring	100.00
fixed	cond		(Intercept)	-4.28	0.63	-5.65	-3.16	summer	50.00
fixed	cond		nr_pools_within	0.04	0.01	0.02	0.06	summer	50.00
fixed	cond		desiccation_dynamic	-0.05	0.21	-0.48	0.34	summer	50.00
ran_pars	cond	island	sd__(Intercept)	1.46	0.59	0.65	2.92	summer	50.00
ran_pars	cond	island:pool	sd__(Intercept)	1.24	0.11	1.03	1.48	summer	50.00
ran_pars	cond	year	sd__(Intercept)	0.40	0.10	0.23	0.62	summer	50.00
fixed	cond		(Intercept)	-4.96	0.71	-6.47	-3.69	summer	100.00
fixed	cond		nr_pools_within	0.04	0.01	0.02	0.06	summer	100.00
fixed	cond		desiccation_dynamic	-0.05	0.21	-0.48	0.34	summer	100.00
ran_pars	cond	island	sd__(Intercept)	1.59	0.59	0.77	3.05	summer	100.00
ran_pars	cond	island:pool	sd__(Intercept)	1.24	0.12	1.02	1.48	summer	100.00
ran_pars	cond	year	sd__(Intercept)	0.40	0.10	0.23	0.63	summer	100.00

3 Supplemental figures

3.1 For methods

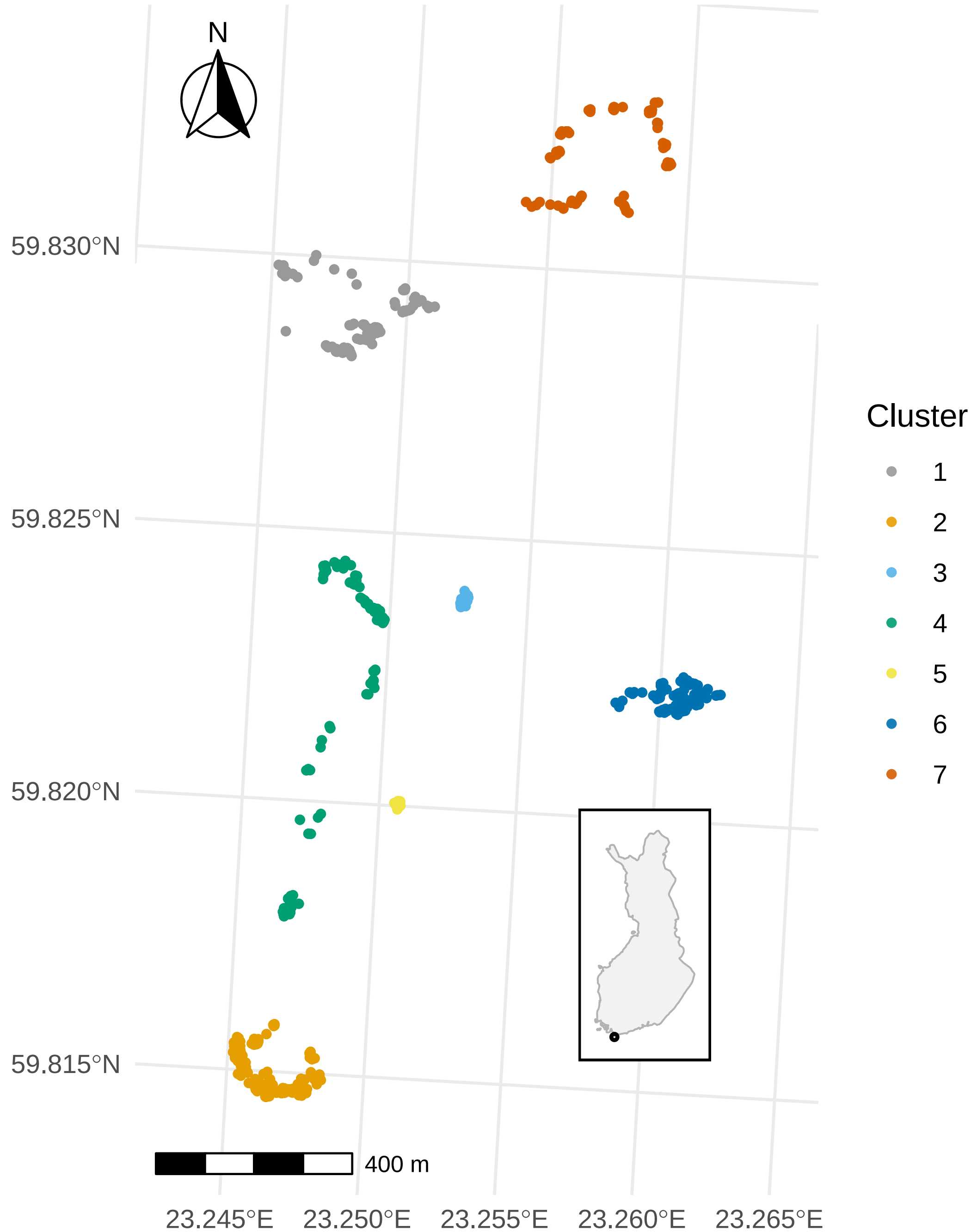

Fig A: **Map of the Tvärminne study area.** Rock-pool locations (n = 401) across 14 islands grouped into 7 island clusters. Inset: location of the pools on the southern coast of Finland. Map data were obtained from the Natural Earth public-domain dataset (`https://www.naturalearthdata.com`) and accessed using the `rnaturalearth` and `rnaturalearthdata` R packages.

3.2 For results

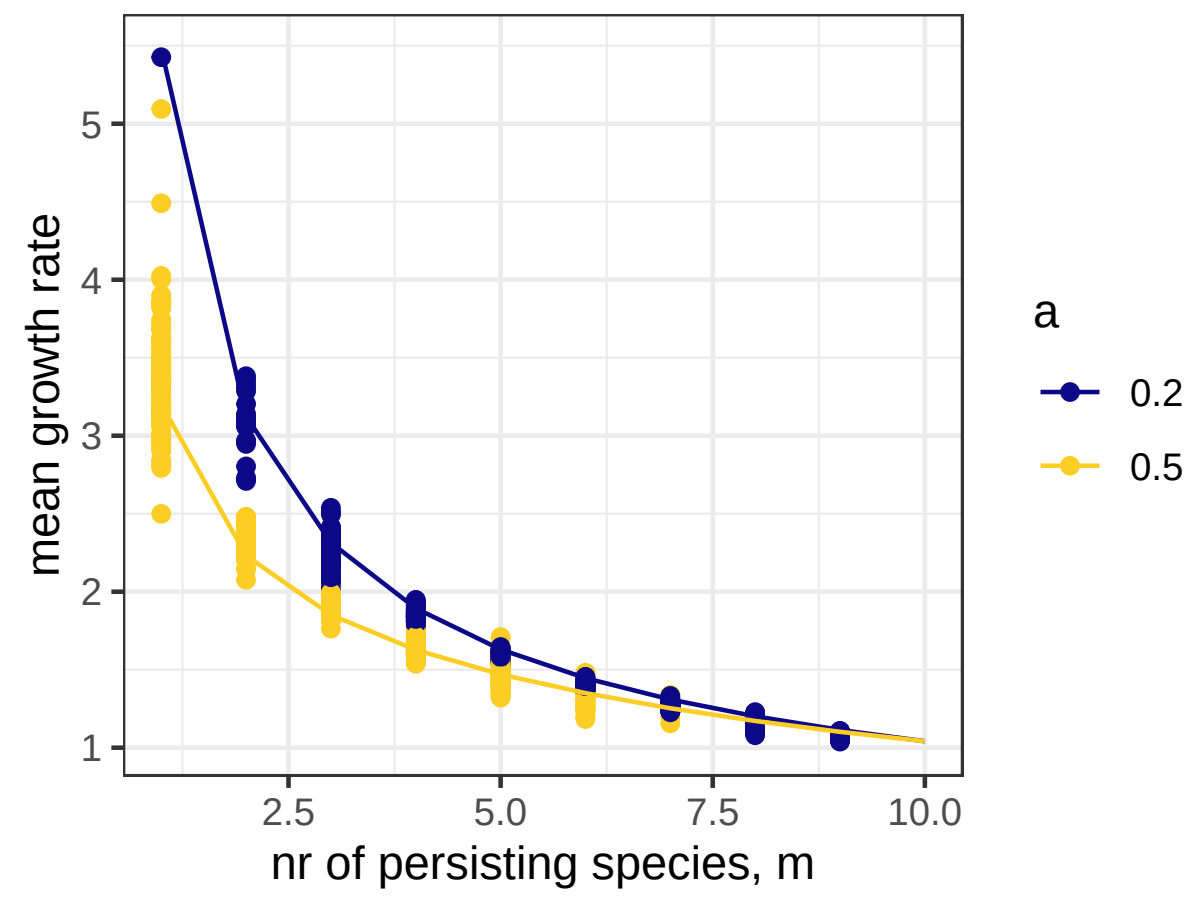

Fig B: **The approximation for $\bar{r}^{(l)}_m$ (Eq. S30) matches simulated values across m.** Points: simulated values of the mean growth rate of the m locally persisting species, obtained from the simulation protocol outlined in the methods (subsetting to $d = 10^{-6}$ to switch off dispersal). Line: analytical approximation (Eq. S30) with $x = 0.45$.

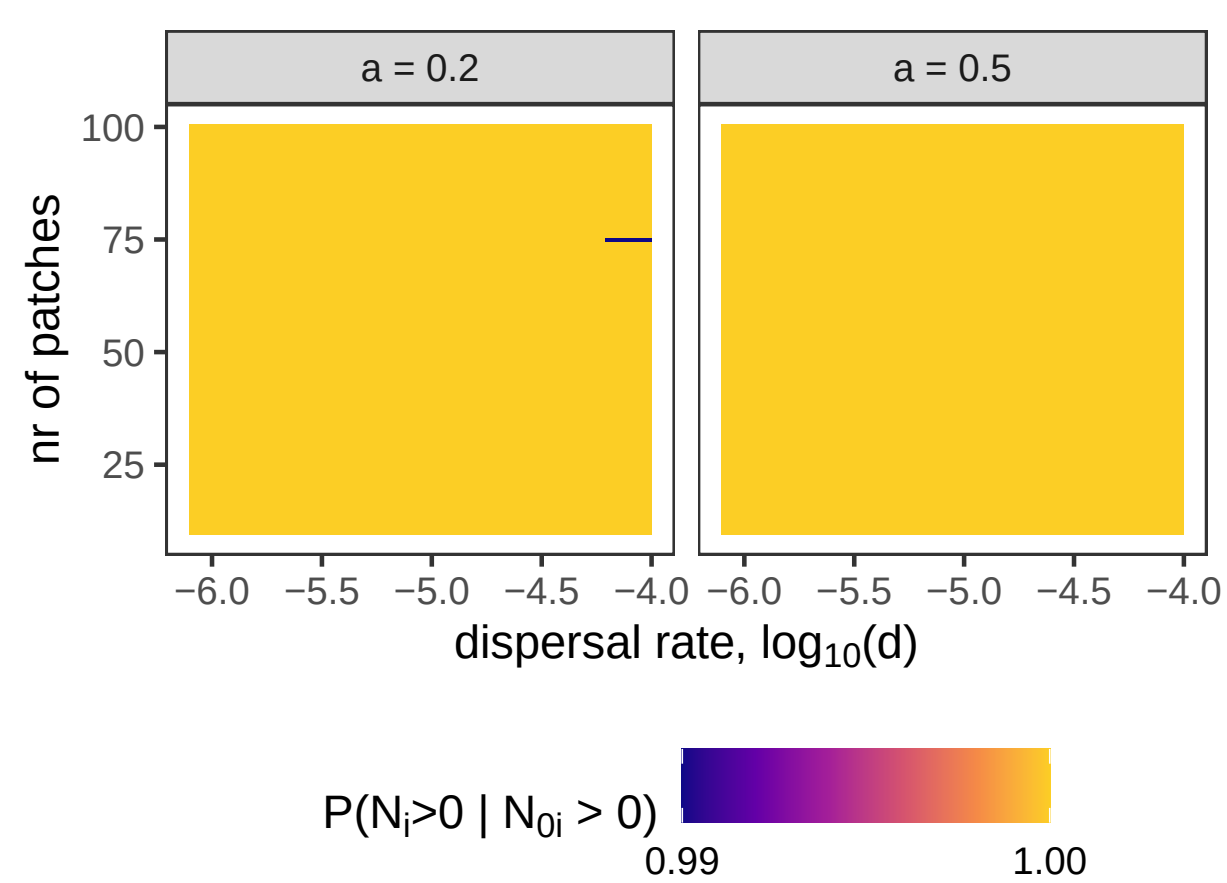

Fig C: **Species persisting without dispersal almost certainly persist with dispersal as well.** Numerical evaluation of Eq. S34 confirms that $P(N^{(l)}_i > 0 \mid N^{(l)}_{0,i} > 0) \approx 1$ across the parameter space considered, provided local interspecific interactions are weaker than intraspecific interactions ($a < 1$), interactions are diffuse, and species are regionally equivalent. This result justifies focusing, in the main text, on the exclusion case as the relevant driver of community-level persistence under weak dispersal.

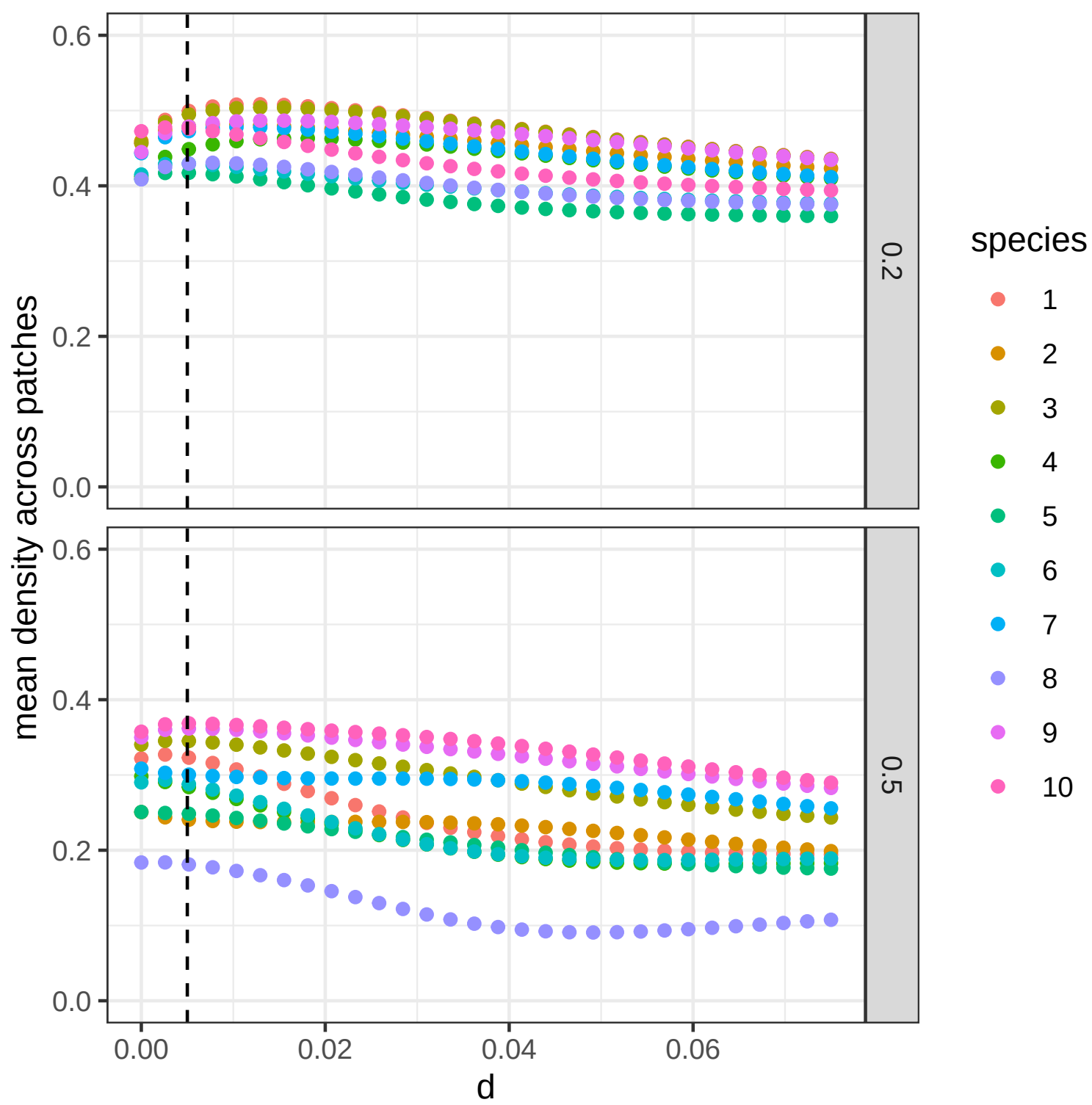

Fig D: Simulation with the main model for $n = 10$, $p = 50$, two values for a (diffuse competition, identical across patches), and assuming regional equivalence of the growth rate. Dispersal rate d affects mean biomass in a nonlinear way. Only when d is small enough (up to ≈ 0.005; dashed line) can this effect be approximated by a linear function.

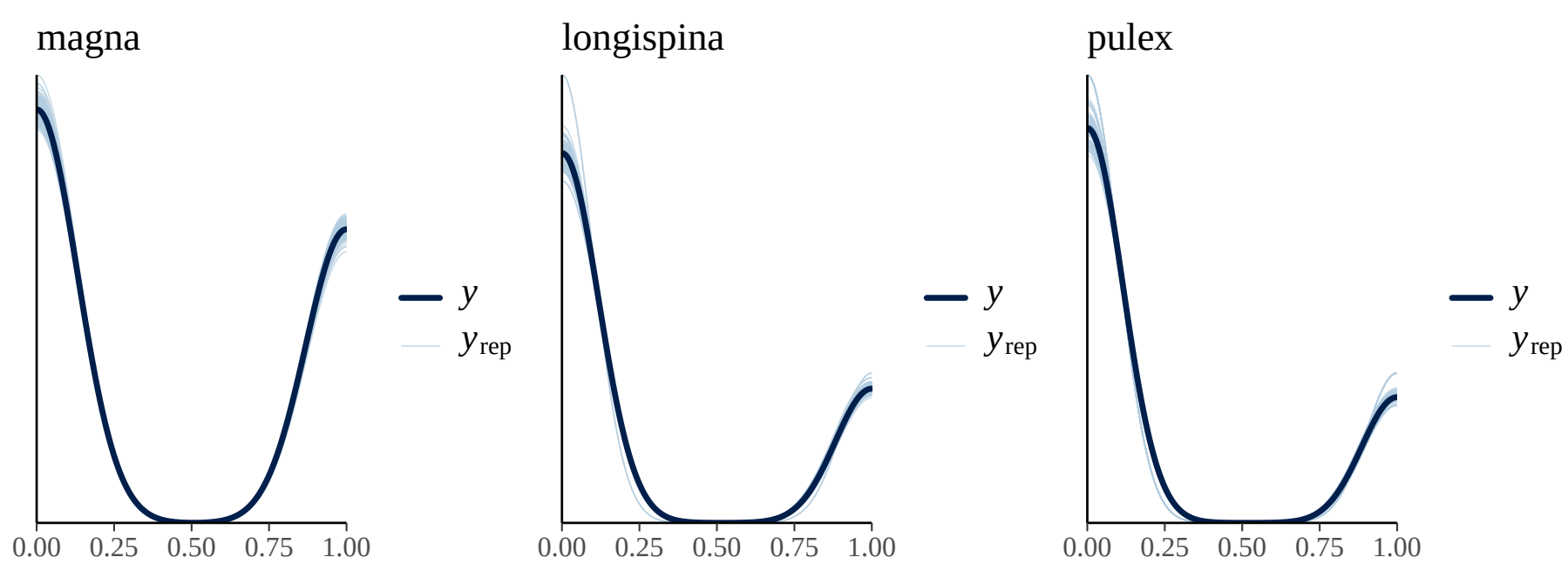

Fig E: **The model adequately reproduces the observed distribution of occurrences across species.** Posterior predictive checks comparing the observed data (y, dark lines) with replicated data from the posterior predictive distribution (y_{rep}, light lines) for each focal species. Close agreement between observed and replicated distributions indicates that the Bernoulli hierarchical model captures the main features of the data, including the prevalence of low probabilities and occasional high-occurrence events.

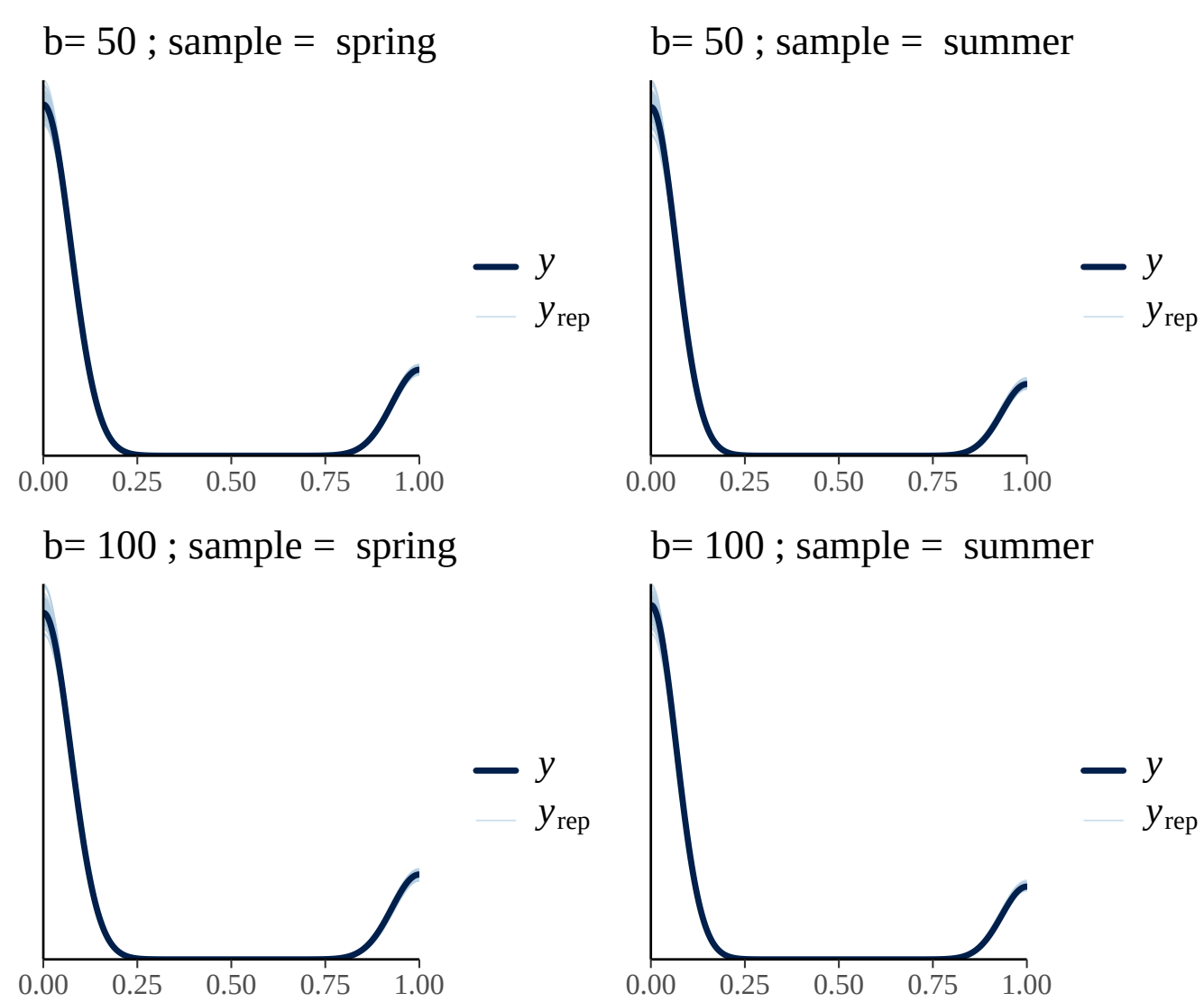

Fig F: **The proportional-occurrence model reproduces the observed data across seasons and spatial scales.** Posterior predictive checks comparing observed outcomes (y, dark lines) with replicated data from the posterior predictive distribution (y_{rep}, light lines) for models fitted at two spatial scales (50 m and 100 m) and in spring and summer. The close correspondence between observed and replicated distributions indicates that the model captures the overall shape and prevalence of low and high occurrence probabilities across conditions.

References

1. Grilli, J. *et al.* Feasibility and coexistence of large ecological communities. en. *Nat. Commun.* **8,** 14389. ISSN: 2041-1723. doi:10.1038/ncomms14389 (2017).
2. Song, C., Rohr, R. P. & Saavedra, S. A guideline to study the feasibility domain of multi-trophic and changing ecological communities. *Journal of Theoretical Biology* **450,** 30–36. ISSN: 0022-5193. doi:https://doi.org/10.1016/j.jtbi.2018.04.030 (2018).
3. Serván, C. A., Capitán, J. A., Grilli, J., Morrison, K. E. & Allesina, S. Coexistence of many species in random ecosystems. *Nature Ecology & Evolution* **2,** 1237–1242. ISSN: 2397-334X. doi:10.1038/s41559-018-0603-6 (2018).
4. Deng, J., Taylor, W. & Saavedra, S. Understanding the impact of third-party species on pairwise coexistence. en. *PLoS Comput. Biol.* **18** (ed Mitri, S.) e1010630. ISSN: 1553-7358. doi:10.1371/journal.pcbi.1010630 (2022).
5. Dubart, M. *et al.* Species coexistence and overlapping distributions in a metacommunity are compatible with niche differences and competition at a local scale. *bioRxiv,* 2020.07.10.196758. doi:10.1101/2020.07.10.196758 (2020).
6. Orsini, L. *et al.* The evolutionary time machine: Using dormant propagules to forecast how populations can adapt to changing environments. *Trends in Ecology & Evolution* **28,** 274–282. doi:10.1016/j.tree.2013.01.009 (2013).
7. De Meester, L., Louette, G., Duvivier, C., Van Damme, K. & Michels, E. Genetic composition of resident populations influences establishment success of immigrant species. *Oecologia* **153,** 431–440. doi:10.1007/s00442-007-0721-3 (2007).
