# Supplementary material for: Weak dispersal and landscape size inevitably promote local biodiversity in heterogeneous metacommunities of competing species
Source: PLoS Comput Biol. 2026 Oct 16;22(10):e1014028. doi: 10.1371/journal.pcbi.1014028 (PMC13705597; doi:10.1371/journal.pcbi.1014028)
Supplement: Supplementary file 2 [file pcbi.1014028.s002.pdf]

**Review for manuscript "Weak dispersal and landscape size inevitably promote local biodiversity in heterogeneous metacommunities of competing species" (PCOMPBIOL-D-26-00366).**

In this study, the authors investigate if a positive effect of (weak) dispersal on biodiversity, more specifically, coexistence of competing species, can be analytically shown. Such a positive (or hump-shaped) dispersal-diversity relationship is a widely studied concept across ecology. Here, the authors present an analytical approximation of the probability for persistence and coexistence of species from a classic Lotka-Volterra competition model, extended by diffusive dispersal of the species among discrete, heterogeneous habitats in metacommunity networks. To analytically approach their question, they take a number of constraining assumptions, and thus can find expressions for species-level persistence and community coexistence probabilities that indeed depend on the dispersal rate of the species, but also on the patch number (landscape size) and the feasibility domains of the species. Furthermore, they test whether their analytical approximation holds for ecologically more realistic scenarios, therefore relaxing their previously made assumptions in numerical simulations of the metacommunity competition model. Lastly, they compare their findings from the model study against the predictors for coexistence in a dataset of competing *Daphnia* species in rockpool metacommunities.

Altogether, the authors present a quite comprehensive study design, combining an impressive range of analytical and computational methods to answer the research question at hand. In my opinion, the major challenge with this work is to structure and present the analytical consideration, which is the main body of the work, but also the numerical simulations and data analysis in a way that the process and findings are accessible to a first-time reader who might be less familiar with some of the ecological concepts or methods used here (detailed in points 1 and 2 below). Content-wise, some of the analytical assumptions and their consequences for the model need better explanations and the comparison between theory and data still requires some attention (points 3 and 4). I apologise for the length of these comments, but hope that the provided details can aid the authors in improving the clarity and accessibility of their manuscript.

1. **Separation of analytical considerations between Methods, Supplementary Methods and Results sections**

The current structure of the analytical considerations gives me the strong impression that the text had originally been developed as one consecutive section that later has been split up between the Methods, Supplementary Methods (S1) and Result sections. However, it was impossible to follow each of these sections without considering the others side by side. It will be necessary to make sure that both the main text and the supplementary information are standalone documents, where all necessary concepts, equations, variables, parameters etc. are introduced regardless of their use in the respective other, and the goals and methods of the analysis are clearly explained in both. Furthermore, the current separation and order of information between analytical Methods and Results does not facilitate the clarity of the manuscript. A further drawback is that the analytical considerations divide the numerical and statistical methods and results, making it harder to follow these parts as well.

If formally possible, the authors thus might consider a separate section after the Introduction for all the parts of their 'Analytical considerations', before presenting methods and results on the complementary numerical simulations and data analysis.

Due to the integral importance of the supplementary information, specifically S1 for the findings of this study, I comment on aspects of it subsequently as well. I reference chapters, equations and figures with "S", to distinguish them from the main text elements.

Below are a few prominent examples of where the structure needs to be reconsidered:

1.1. The analytical considerations in the main text Methods and S1 treat two separate ecological case scenarios: Persistence of a species with dispersal 1) if it would go extinct without dispersal and 2) if it would persist without dispersal. How this sums up to the overall persistence probability of any given species is, however, only properly mathematically defined in Eq. 6 of the main text, which is at the beginning of the Results section (l. 172-l178). In my opinion, this equation is the basis of the analytical considerations, and thus should appear earlier in the Methods, and also again in S1. This becomes obvious when one notes that the terms of Eq. 6, resembling the individual case scenarios, are referenced verbally and mathematically, many times throughout the supplementary Methods S1 (e.g., S1, l. 38-40, l. 110, l. 122).

1.2. S1 starts with setting the left-hand side of 'the main model in the main text' to zero (Eq. S1, l. 18f) – an introduction of this model (like given in the main text, Eq. 1 and l. 33-37), including definition of variables, indices and parameters used, a proper reference to the main text, or the general goal of the analysis is missing here. The other way around, a variable $m$ is introduced for the number of locally coexisting species in a community $C$, and based on that, fractions $f(m)$ are defined (S1, l. 59ff). These variables are also used in the main text results and equations (e.g., line 223, 227, Eqs. 9,10), but not or differently defined here (l. 226: I believe $f(m)$ should denote the fraction of patches where $m$ species coexist, and only the whole set of these fractions denotes the distribution of persisting species?) (see also comment on feasibility domain).

1.3. Approximations of persistence probabilities for species that would be excluded or persist without dispersal (l.99-100, l. 203-217). Why were the results of both of these approximations not presented as equations in the main text? For completeness, since the main text Figure 1 was generated for the exclusion scenario and the other has no result figure in the main text, it would be reasonable to display the result equations in the main text as well and then discuss their implications for the role of landscape size etc. In this context, there is a parameter $x$ introduced in S1 that is important to assess the mean growth rate of the persisting community of $m$ species $\bar{r}_m^{(l)}$ (S1, l. 81-99). Specifically, in S1, l. 110-119, Eq. S27 is used to calculate part of Eq. S12, which corresponds to the calculation of persistence probabilities of a given species. To do this, knowledge of $x$ and $f(m)$, in addition to the basic model parameters, seems necessary. The authors describe the computation of $f(m)$ subsequently, but only in context of Eq. S12's denominator. It remains unclear how $x$ or $\bar{r}_m^{(l)}$ are estimated for the results shown in the main text. It would, however, be very interesting to get some more insight into what the parameter $x$ means for the applicability of the method the authors present here to other ecological scenarios.

## 2. Accessible explanations of important ecological and methodological concepts

The authors have been rather sparse with explanations/introductions of ecological and theoretical concepts. Many of the readers of *PLOS Computational Biology* might, however, not be familiar with the mathematical and ecological handling of a LV competition or metacommunity model. Below, I have summarised the concepts for which I noticed missing explanations or definitions:

2.1: The clarity of the main text and S1 would greatly benefit from concise, verbal and mathematical definitions of **weak ($a_{ij} < a_{ii} = 1$), diffusive (all $a_{ij} = a$) and preemptive ($a_{ij} = a_{ii} = 1$) interspecific competition** at first mention. While the assumption of weak competition is made in the main text (l. 98-99), the assumption of $a_{ii} = 1$ is only stated implicitly, and it is not expressed in the model equations, starting already with Eq. 1 where it is omitted right away. While this might be common practice with LV competition models, this lowers accessibility for readers from different backgrounds. In S1, the information that $a_{ii} = 1$ is used again in the matrix equations, specifically the elements of the interaction matrix (Eq. S15, S1 l. 70 and subsequently). For completeness, it might thus be good to start the model equations including $a_{ii}$.

2.2. A definition of the term **'preemptive' competition** is necessary at first occurrence, alongside of explanations of why it is necessary to address it in the dataset (see comment 4, l. 152 f) and which case scenario it represents in the model (strong competition, $a_{ij} = a_{ii} = 1$) in a clearer way. Right now, this information is often only implicitly given (cf. l. 108-109).

2.3. The authors claim to show how multispecies patch occupancy relates to the framework of **feasibility (domains)** (l. 62-64, 274-76). While they indeed introduce an estimate of the feasibility into their approximation, as a function of $n$ (Eq. 11), they never really explain the concept to the reader (verbally or mathematically), and that and why exactly it can be used here as a replacement for the persistence probability of a species in isolation. In the discussion, they refer to 'feasibility' as 'having a positive pop. density' (l. 341), which seems a bit too general. In S1, the explanations of the feasibility domain are scarce as well, and feasibility is only defined as a function of $m$.

2.4 The terms **biodiversity, local and regional coexistence, multispecies persistence, co-occurrence and community-level/multispecies patch occupancy** are used very interchangeably throughout the manuscript. Clarity of the manuscript would benefit from consistent use of fewer terms and early, concise definitions. More generally, the manuscript mixes patch-dynamics and mass-effect perspectives on metacommunity dynamics and coexistence mechanisms, and this is also reflected in the abovementioned terminology.

### 3. The 'regional equivalence' assumption

The 'regional equivalence' of the species is a central assumption throughout the analytical work in this manuscript. In l. 91-96, it is introduced as equal mean of species' growth rates of species across habitats, albeit stochastic differences of $r_i^{(l)}$ across species and local habitats (as proxy for spatial heterogeneity). It would be helpful if the authors could add a few extra sentences to introduce the concept, give an example of how growth rates could be distributed in a small habitat network with hosting a few species (this might be greatly supported by a conceptual figure!), and explain the consequences of this assumption.

In S1, the terms used for central assumptions like 'regional equivalence' are not defined in words or equations (e.g. l. 53 starts with 'because of regional equivalence' without ever defining what that is). Their use in simplifying the equations is often only described very sparsely, and their implications for growth rates and population densities without dispersal is not explained well (see also other comments).

Consequence 1 of regional equivalence of the growth rates: regional equivalence of population densities? S1, l. 53f reads 'because of regional equivalence, the first of these sums [the total

density of the focal species across all disconnected patches but the focal one] is the same for all species.' This is a nontrivial assumption and not further explained in the following. Why would the stochastic combinations of intrinsic growth rates among patches and species not lead to differing competitive scenarios and thus varying regional densities among species, even under the assumption of diffuse competition?

Consequence 2: The strong competition scenario ($a \rightarrow 1$) implies superiority of any species in only a single habitat patch, while it is outcompeted in all others (S1, l. 148-150): does regional equivalence imply that a species $i$ cannot by chance have the largest intrinsic growth rate $r_i^{(l)}$ in more than one habitat? As long as the regional mean is balanced through other habitats with very low growth rates (drawn from a truncated normal distribution), this scenario should be unlikely, but possible? What about systems with more patches than species? I believe at most in the latter case, this assumption does not hold.

4. **Comparability between model and data analysis**

The way the analysis of model and data were presented here still made a quite disconnected impression. In the Introduction, comparative analysis of the Daphnid metacommunity is only briefly mentioned at the very end. The clarity of the study would greatly benefit from a statement regarding why this exact dataset is suitable for comparison against the model.

Importantly, I noticed a conceptual mismatch between what is assessed from the model and the data. While from the model, the probability for all species to persist in a given local patch is assessed as the final methodological step (community-level patch occupancy); this exact scenario is excluded a-priori from the data analysis of the Daphnid communities since it was so rare ('only 0.33 % of samples, l.148-149), and only the probability and predictors for pair-wise co-occurrence were assessed.

Furthermore, the model analysis only treated a case for $n = 10$ species in $p \geq 10$ habitat patches (l. 100f, 107), while the Daphnid metacommunity consisted of 3 species in varying number of habitats. Why was $n$ never varied (and the patch number never decreased for a smaller $n$) in the analytical considerations or numerical simulations, especially to aid comparison with the data analysis?

Lastly, providing some more context on Daphnids (an organism group that might not be familiar to all of the readers) might increase accessibility. Specifically, an extended explanation in the Methods of why pre-emptive competition was assumed/tested and why and how the desiccation rates of the rock pools can be used as proxies for the dispersal rates (with higher desiccation rate = higher dispersal rate?) would be helpful. Concerning the latter: the desiccation rate in the data seems to be a habitat-based characteristic (L. 143-145), but dispersal rates were before estimated from species traits (characteristic distances) and interpatch spatial distance, i.e., relative link length (l. 120 f); how are these two metrics comparable?

**Miscellaneous, minor comments:**

- l. 37: since immigration from one patch and emigration into another patch are modelled at the same time (instantaneous movement), $d$ does not measure how quickly individuals move among habitats, but rather, how frequently?
- For completeness, it would be good if the first simplifying assumption(s) made for all of the analytical considerations, the 'well-mixed-assumption', and equal 'characteristic distances' among species, was stated alongside the model introduction (Eq. 1 and S1);

After all, these allow the authors to use a single parameter $D$ (or, $d$) to describe movement of all species among all habitats. Only the Methods for the numerical simulation describe these assumptions as they are relaxed (l. 112-113, 119-123)

- l. 99-100, 103-104 consider shifting the information in S1.2.3 to the main text
- l. 106 f: For the relaxed assumptions, simulations had to be run with an extended version of the model described by Eq. 1, for example for the more complex dispersal structure; for completeness, the Methods or Supplementary should state the exact model used for simulations.
- l. 196: Eq. 7 does not calculate probabilities; consider rephrasing this part or generally explaining, how this shift from calculating densities to probabilities is made
- l. 198 – 202, also 210f: the effect of the spatial network size ($p$) is not a factor in Eq. 7, but only in the sum of the numerator, thus only indirectly affecting a species' local density by increasing its regional density in isolation. Whether this is the case should depend on how likely it is that a species can persist in more patches when the network is larger – and this again depends on the interaction strength (see limiting cases considered in S1, l.145-150). The relation thus seems less straightforward than noted here and should be at least explained in more detail.
- Figure 1: The figure should be understandable without the main text or SI. Please add that these are analytically estimated values and note the equations and constraining assumptions this is based on. Consistently define parameters and indices in the legend, e.g., that a refers to interspecies interaction strength, and that $N$ is given for a species $i$ in patch $l$
- L. 234: specify that it still is the community-level patch occupancy that is calculated; From which equations and using which methods is this done? Especially the estimation of feasibility domains?
- Figure 2: why is the community-level patch occupancy so sharply increasing (almost binary from 0 to 1?) around $d = 10^{-4}$? Could we see an additional panel for $a = 1.0$?. Legend reads "values up to 0.005 are weak"; of what parameter?
- Figure 3: Legend: what does 'Dispersal is no longer implicit' mean? Also, missing 'no' in the row description
- L. 248-250: this result could use a figure reference
- L. 258-260: Equation or figure reference for the regression model and its results?
- L. 263: can this value be seen in a figure? In Fig. 5 it looks like this value should be lower
- Figure 4: please revise the axis labels and legends to display complete information: from which equation/model was this assessed, what is the tested response variable, what is 'focal' and 'value', what effects are fixed and random
- L. 262-268 Why are the posterior distributions of predictors from the Daphnid co-occurrence model (Eq. 5) not shown in a figure in the main text?
- L. 277: microcrustacean is used as a descriptor for Daphnid communities for the first time here
- L. 285: should read 'exclusion rate' instead?
- L. 285-86: this information is quite disconnected here and I believe it contradicts the model assumptions of this study. In l. 189 and also the SI it is stated that this denominator is always positive and, most importantly, never zero. Also, in Eq. S23, it is assumed that for non-persisting species, intraspecific growth is always lower than interspecific competition. If needed, this information might fit better in the Methods
- L. 288: if the increase in regional density comes from dispersal alone, where do the individuals come from? Cf. Equation also S30 and comment on S1 below

- L. 291-96: is this effect observed because the model is designed in a way that a larger network size makes local persistence more likely by chance and thus regional density stochastically increases? Intuitively, this indeed reads like stochastically assigning larger realised niches to species. Please clarify.
- L. 302-03. I believe this is only true under the 'well-mixed' assumption, where new patches will be connected to all other existing patches
- L. 303-307: already existing community-level extensions of metapopulation models often clearly distinguish between stochastic extinctions from the environment and exclusion due to interspecific competition. I would be careful to mix these two concepts together here.
- L. 320: 'to the same Daphnia data' - as the dataset analysed here?
- l. 326-333: Desiccation rates did not affect co-occurrence: remind the reader that this is a proxy for the dispersal rate – this result is not contextualised here
- L. 330: the effect of dispersal on what?
- L. 342-344: please define what is meant by 'stability' (temporal, dynamical?)
- L.351: please consider rephrasing, there was no (mathematical) proof involved in this analysis
- L. 366-67: where was this shown in the results (figure reference)? The effect of a higher/lower growth rate was not mentioned before?

**Comments on Supplementary:**

Generally, S1 is written very densely and often short of explanations of 'why' a specific assumption is made or what this implies for an equation. For example, l.125 reads that 'the per capita growth rate at equilibrium is zero'. The consequence of this is that one can drop the first term of Eq. 9; this is not verbally noted, and in the first line of Eq. 28 executed together with the consequences of the regional equivalence and diffusive interaction assumptions. Expressions from previous equations are inserted without clear references (e.g. that Eq. S12 inserts Eq. S11 into Eq. S4, or that Eq. S26 is derived from Eq. S14 and Eq. S22) or assumptions are transferred from the main text. While this all not invalidates the methods presented here, there is a lot of methodological expertise required from the reader and capability to keep track of numerous details mentioned elsewhere to follow this analysis.

- L. 24: the weak d assumption is only needed in the next step (for truncation of the power series beyond linear terms)
- L. 50-56: Please revise this paragraph (1.2.2.), it is hard to follow these assumptions, the conclusions drawn from them and why the next steps are necessary
- Eq. S13: should the left side of this equation not also have a second sum over all species, and then divide it by $n$? cf. l. 54-55. If this expression can immediately be simplified, please verbally explain why
- Eqs. 16-17: please check the notation of the row- and column vectors. The current notation implies $u = v^T$, which then are multiplied in Eq. 16
- Eq. 19-20: the elements of the inverse of the interaction matrix have the same notation as the original interaction coefficients; this is very hard to read; consider using a different letter
- SI L. 61-62: please explain the 'feasibility domain size', and introduce the 'diffuse interaction' assumption, perhaps even with an equation statement such as $a = a\_ij < a\_ii = 1$
- Eq. S15: to be able to understand why the diagonal elements of $\boldsymbol{A}$ are $= 1$, the reader needs to know how an interaction matrix is built, specifically, that this one is of size

$m \times m$, that the intraspecific interaction coefficients $a_{ii}$ are on the diagonal; and lastly, that $a_{ii} = 1$ and therefore it was omitted in Eqs. S1 through S6.

- L. 75f and Eq. 21: consider reminding the reader that Eq. 19 and 20 are inserted into equation 6, and that all this assumes there is no dispersal; why does $r_j$ in the first line have no patch index?
- L. 89-102: inconsistent use of patch-index $(l)$ in $\bar{r}_m$ notation? If it is intentional, where is the difference between the two?
- SI, L. 121-22: 'We then sample $r_i^{(l)}$ from the distribution of growth rates $g^{(l)}$ (truncated normal, given in the main text)' – this relies too much in information presented in the main text
- Figures of supplementary material S1 have no numbering or description. For example, it is unclear whether the first of these figures presents simulated and or/ calculated data points from Eq. 25
- L. 135: I do not see where this is explained in section 1.2.1.
- L. 136-140, Eq. 29: I think this paragraph follows the same steps as l.65-75, please remind the reader of that, and also, what the goal of dealing with this matrix equation is (to solve for $\hat{N}_{1,i}^{(l)}$). As it is, it is very hard to follow.
- L. 142 and Eq. 30: It would be good to remind the reader that this is a linear approximation (building on Eq. 4). If $n = m$, the first term inside the bracket of the numerator can become $= 0$.
- The paragraph (l. 145-159) argues for why the second term of Eq. 30 should be positive or zero for all parameter combinations, when considering two extreme cases of strong and weak interactions. In the strong competition case, the assumptions imply that $N_i^{(l)} \geq N_{0,i}^{(l)}$. If this holds for any species $i$ on any patch $l$, that means that dispersal is creating individuals to add to a population out of nowhere. It would be interesting to see if this also happens for the intermediate interaction parameters a, from the actual approximated populations densities with and without dispersal, instead of just the probability of persistence (Figure below l. 159)
