# Supplementary material for: Weak dispersal and landscape size inevitably promote local biodiversity in heterogeneous metacommunities of competing species
Source: PLoS Comput Biol. 2026 Oct 16;22(10):e1014028. doi: 10.1371/journal.pcbi.1014028 (PMC13705597; doi:10.1371/journal.pcbi.1014028)
Supplement: Supplementary file 3 [file pcbi.1014028.s004.pdf]

Response to reviewers — Manuscript PCOMPBIOL-D-26-00366

"Weak dispersal and landscape size inevitably promote local biodiversity in heterogeneous metacommunities of competing species"

Frederik De Laender, Andrew Gonzalez, Olivia Bleeckx,
Dieter Ebert, György Barabás

June 1, 2026

Dear Dr. Martinez-Garcia,

We thank you and the three reviewers for the thoughtful evaluation of our manuscript. The comments have substantially helped us improve the manuscript and its Supporting Information (SI). Below we address every point raised by each reviewer. The reviewers' comments are reproduced *in italics*, followed by our **Response:** response in blue. We are uploading the following files:

- This rebuttal: Rebuttal.pdf.
- The revised manuscript with track changes in the originally submitted format: Main-r1-track-changes.pdf. *Note: The line numbers in the rebuttal refer to the line numbers in that marked-up version.*
- The revised manuscript in the PLOS format: Main-r1-PLOS.pdf.
- The LaTeX Source File: main-r1-PLOS.tex.
- The revised Supporting Information: Supplements-r1.pdf.

We hope that the revised manuscript now meets the standards of *PLOS Computational Biology* and thank again the editor and reviewers for their time.

Sincerely,
Frederik De Laender, on behalf of all co-authors.

Editor's comments and Journal Requirements

(1) Please ensure that the CRediT author contributions listed for every co-author are completed accurately and in full. At this stage, the following Authors/Authors require contributions: Frederik De Laender, György Barabás, Olivia Bleeckx, Dieter Ebert, and Andrew Gonzalez. Please ensure that the full contributions of each author are acknowledged in the "Add/Edit/Remove Authors" section of our submission form.

Response: We have completed and updated the CRediT author contributions both in the submission portal and in the Author Contributions section of the manuscript.

(2) We ask that a manuscript source file is provided at Revision. Please upload your manuscript file as a .doc, .docx, .rtf or .tex. If you are providing a .tex file, please upload it under the item type 'LaTeX Source File' and leave your .pdf version as the item type 'Manuscript'.

Response: We upload the revised manuscript, as well as a track changes version.

(3) Please provide an Author Summary. This should appear in your manuscript between the Abstract (if applicable) and the Introduction, and should be 150–200 words long. The aim should be to make your findings accessible to a wide audience that includes both scientists and non-scientists. Sample summaries can be found on our website under Submission Guidelines

Response: Added between the Abstract and the Introduction.

(4) Please upload all main figures as separate Figure files in .tif or .eps format. For more information about how to convert and format your figure files please see our guidelines

Response: We will upload high-resolution TIFF versions of all main figures at resubmission.

(5) We have noticed that you have uploaded Supporting Information files, but you have not included a list of legends. Please add a full list of legends for your Supporting Information files after the references list.

Response: A list of SI legends has been added after the reference list in the revised main manuscript.

(6) Please amend your detailed Financial Disclosure statement. This is published with the article. It must therefore be completed in full sentences and contain the exact wording you wish to be published. 1) State the initials, alongside each funding source, of each author to receive each grant. For example: "This work was supported by the National Institutes of Health (####### to AM; ###### to CJ) and the National Science Foundation (###### to AM)." 2) State what role the funders took in the study. If the funders had no role in your study, please state: "The funders had no role in study design, data collection and analysis, decision to publish, or preparation of the manuscript." 3) If any authors received a salary from any of your funders, please state which authors and which funders. If you did not receive any funding for this study, please simply state: "The authors received no specific funding for this work."

Response: We updated the Financial Disclosure on the submission form.

(7) Your current Financial Disclosure states, "Yes ↪ Please add funding details. This research used resources of the 'Plateforme Technologique de Calcul Intensif (PTCI)' located at the University

of Namur, Belgium, which is supported by the FNRS-FRFC, the Walloon Region, and the University of Namur (Conventions No. 2.5020.11, GEQU.G006.15, 1610468, RW/GEQ2016 et U.G011.22). ↪ *Please select the country of your main research funder (please select carefully as in some cases this is used in fee calculation). BELGIUM – BE". However, your funding information on the submission form indicates recieving no funds. Please indicate by return email the full and correct funding information for your study and confirm the order in which funding contributions should appear. Please be sure to indicate whether the funders played any role in the study design, data collection and analysis, decision to publish, or preparation of the manuscript.*

Response: We have provided the corrected funding information by return email. The funders had no role in study design, data collection and analysis, decision to publish, or preparation of the manuscript.

Reviewer #1

The maintenance of biodiversity is a central question in ecology, and understanding how spatial dynamics shape species coexistence is critical for predicting biodiversity responses to global change. Previous work has extensively explored dispersal effects in metacommunities, often through simulation-based approaches. Going beyond, this study attempts to advance our understanding by combining analytical investigations with empirical data. Their analyses showed that weak dispersal and smaller landscape sizes consistently increased local species richness. I appreciate the clarity of the writing and the ambition of the research objective. That said, I have a number of concerns regarding the implications and scope of the analytical results.

Response: We thank the reviewer for the positive overall assessment and for the constructive points below. We assume that the reviewer means to say "weak dispersal and larger landscape sizes".

Major comment 1 — Framing and novelty of the results

First, the paper aims to "explain the well-documented positive effect of weak dispersal on local species persistence in heterogeneous landscapes" (lines 53–54). I was somewhat surprised by this framing, as this pattern seems almost definitional: under weak dispersal, the regional species pool can contribute to local diversity without substantially altering local species interactions or causing competitive exclusion. While the analytical derivations (e.g., Equations 6, 8–10) certainly formalize this intuition, they appear to restate it in probabilistic terms rather than offering new biological insight. As a result, many of the predictions derived from these equations read as expected outcomes (e.g., lines 189–191, 210–212, 215–216, 284–287, 323–324). An exception is Equation 7, which usefully quantifies how local population size depends on dispersal relative to demographic rates and interspecific interactions. However, even here, the qualitative insights remain broadly consistent with existing expectations.

Response: We agree that the positive effect of weak dispersal on local diversity is widely anticipated in verbal ecological theory. Our contribution is not that we predict the direction of this effect, but that we derive an explicit, quantitative expression of local persistence, with an explicit dependence on landscape size p, and that we show how community-level patch occupancy maps onto the feasibility domain $\Xi(n)$ from structural coexistence theory. To our knowledge, neither the explicit scaling with p nor the link to feasibility has been derived before in the way we do and for large competitive metacommunities. We have clarified this on L62 and 91, and transferred methods and results from the supplements to the main text to better highlight the novelty of our contribution (e.g. the decomposition of persistence and coexistence probability).

Major comment 2 — Limiting-case assumption of weak dispersal

Second, the analyses were built on limiting-case assumptions in which dispersal was weak enough to be treated as a minor perturbation. In this regime, total population size remains effectively unchanged, and dispersal primarily influences spatial distribution. While this simplification aids tractability, realistic scenarios usually involve dispersal rates that considerably alter both population sizes and spatial patterns through feedbacks with local dynamics. Capturing such effects would require models that more explicitly integrate local and spatial processes, though I recognize that analytical tractability is not always guaranteed in such cases.

Response: The reviewer is right that our analytical results apply only in the regime where d has a linear effect on local density. We have therefore added an explicit discussion of this limitation at the end of the Discussion (L535).

Major comment 3 — Integration of empirical analyses

In addition, the role of the empirical analyses within the broader study framework could be articulated more clearly. As presented, it is difficult to see how the empirical results align with or validate the analytical findings, which weakens the integration between these two components.

Response: We have revised the last paragraph of the Introduction (L111) and the opening of the empirical Results subsection (L420) to state explicitly (i) why the *Daphnia* rock-pool metacommunity is a suitable model system (long-term census data, metacommunity structure), (ii) which predictions of the theory it can test (effect of a proxy for dispersal rate and landscape size on pair co-occurrence; presence of preemptive competition), and (iii) which it cannot test (three-species coexistence is too rare to permit statistical inference on community-level patch occupancy). We also carried out new simulations with the extended model, parameterized to reflect more specifically the *Daphnia* rock-pool metacommunity, and compared the results to the results based on the statistical analyses (explained on L265).

Minor comments

L251: The ecological relevance of the value 0.01 is unclear. Is a dispersal rate of this magnitude ecologically realistic as a representation of 'sufficiently strong' dispersal?

Response: We have reframed the paragraph that discusses this. Our main point is now that "strong" dispersal in the mathematical sense used in our theory refers to the regime where the weak-dispersal linear approximation fails, not to any particular ecological reality. We added this on L411 and 416. For our new simulations for the Daphnid metacommunity, we also used estimated dispersal rates based on available colonization rates for the same system, to make sure our simulations are relevant for the empirical system (explained on L265).

Figure 1: I was unsure whether the probability shown is evaluated against a threshold or under stochastic environments; otherwise, it is not clear why this value would not always equal 1, as suggested in the main text (lines 189–191).

Response: The probability in Fig. 1C is evaluated against the extinction threshold (10^{-3}), not against zero. We have revised the Fig. 1 and its caption to state this explicitly.

Reviewer #2

In this study, the authors investigate if a positive effect of (weak) dispersal on biodiversity, more specifically, coexistence of competing species, can be analytically shown. Such a positive (or hump-shaped) dispersal-diversity relationship is a widely studied concept across ecology. Here, the authors present an analytical approximation of the probability for persistence and coexistence of species from a classic Lotka-Volterra competition model, extended by diffusive dispersal of the species among discrete, heterogeneous habitats in metacommunity networks. To analytically approach their question, they take a number of constraining assumptions, and thus can find expressions for species-level persistence and community coexistence probabilities that indeed depend on the dispersal rate of the species, but also on the patch number (landscape size) and the feasibility domains of the species. Furthermore, they test whether their analytical approximation holds for ecologically more realistic scenarios, therefore relaxing their previously made assumptions in numerical simulations of the metacommunity competition model. Lastly, they compare their findings from the model study against the predictors for coexistence in a dataset of competing Daphnia species in rockpool metacommunities. Altogether, the authors present a quite comprehensive study design, combining an impressive range of analytical and computational methods to answer the research question at hand. In my opinion, the major challenge with this work is to structure and present the analytical consideration, which is the main body of the work, but also the numerical simulations and data analysis in a way that the process and findings are accessible to a first-time reader who might be less familiar with some of the ecological concepts or methods used here (detailed in points 1 and 2 below). Content-wise, some of the analytical assumptions and their consequences for the model need better explanations and the comparison between theory and data still requires some attention (points 3 and 4). I apologise for the length of these comments, but hope that the provided details can aid the authors in improving the clarity and accessibility of their manuscript.

Response: We thank the reviewer for the careful reading and the extensive, constructive feedback. We address each of the four major points and the many specific comments below.

Major comment 1 — Structure of analytical considerations

The current structure of the analytical considerations gives me the strong impression that the text had originally been developed as one consecutive section that later has been split up between the Methods, Supplementary Methods (S1) and Result sections. However, it was impossible to follow each of these sections without considering the others side by side. It will be necessary to make sure that both the main text and the supplementary information are standalone documents, where all necessary concepts, equations, variables, parameters etc. are introduced regardless of their use in the respective other, and the goals and methods of the analysis are clearly explained in both. Furthermore, the current separation and order of information between analytical Methods and Results does not facilitate the clarity of the manuscript. A further drawback is that the analytical considerations

divide the numerical and statistical methods and results, making it harder to follow these parts as well. If formally possible, the authors thus might consider a separate section after the Introduction for all the parts of their 'Analytical considerations', before presenting methods and results on the complementary numerical simulations and data analysis. Due to the integral importance of the supplementary information, specifically S1 for the findings of this study, I comment on aspects of it subsequently as well. I reference chapters, equations and figures with "S", to distinguish them from the main text elements. Below are a few prominent examples of where the structure needs to be reconsidered:

Response: We agree that the analytical material was hard to follow when split between Methods, SI and Results. We have therefore restructured the Methods and Supplemental Information entirely. Both the main text and the Supplemental Information are now standalone documents.

1.1 — Eq. 6 should appear earlier

The analytical considerations in the main text Methods and S1 treat two separate ecological case scenarios: Persistence of a species with dispersal 1) if it would go extinct without dispersal and 2) if it would persist without dispersal. How this sums up to the overall persistence probability of any given species is, however, only properly mathematically defined in Eq. 6 of the main text, which is at the beginning of the Results section (l. 172-l178). In my opinion, this equation is the basis of the analytical considerations, and thus should appear earlier in the Methods, and also again in S1. This becomes obvious when one notes that the terms of Eq. 6, resembling the individual case scenarios, are referenced verbally and mathematically, many times throughout the supplementary Methods S1 (e.g., S1, l. 38-40, l. 110, l. 122).

Response: We restructured the methods and supplemental information entirely. The former Eq. 6 now does come earlier, and has been complemented by its community-level analogue in the methods.

1.2 — SI starts too abruptly

S1 starts with setting the left-hand side of 'the main model in the main text' to zero (Eq. S1, l. 18f) – an introduction of this model (like given in the main text, Eq. 1 and l. 33-37), including definition of variables, indices and parameters used, a proper reference to the main text, or the general goal of the analysis is missing here. The other way around, a variable m is introduced for the number of locally coexisting species in a community C, and based on that, fractions $f(m)$ are defined (S1, l. 59ff). These variables are also used in the main text results and equations (e.g., line 223, 227, Eqs. 9,10), but not or differently defined here (l. 226: I believe $f(m)$ should denote the fraction of patches where m species coexist, and only the whole set of these fractions denotes the distribution of persisting species?) (see also comment on feasibility domain).

Response: We have rewritten the opening of the SI to (i) reproduce the main model with a full legend of variables and indices, (ii) introduce the most commonly used notation, including m and

$f(m)$. We also synchronized all notation between main text and SI.

1.3 — Show both persistence equations in main text; role of x

Approximations of persistence probabilities for species that would be excluded or persist without dispersal (l.99-100, l. 203-217). Why were the results of both of these approximations not presented as equations in the main text? For completeness, since the main text Figure 1 was generated for the exclusion scenario and the other has no result figure in the main text, it would be reasonable to display the result equations in the main text as well and then discuss their implications for the role of landscape size etc. In this context, there is a parameter x introduced in S1 that is important to assess the mean growth rate of the persisting community of m species $\overline{r}_m^{(l)}$ (S1, l. 81-99). Specifically, in S1, l. 110-119, Eq. S27 is used to calculate part of Eq. S12, which corresponds to the calculation of persistence probabilities of a given species. To do this, knowledge of x and $f(m)$, in addition to the basic model parameters, seems necessary. The authors describe the computation of $f(m)$ subsequently, but only in context of Eq. S12's denominator. It remains unclear how x or $\overline{r}_m^{(l)}$ are estimated for the results shown in the main text. It would, however, be very interesting to get some more insight into what the parameter x means for the applicability of the method the authors present here to other ecological scenarios.

Response: We now present a concise version of both approximations in the main text (an equation summarizing the exclusion case, and a second summarizing the persistence case), with the detailed derivations retained in the SI. The auxiliary parameter x is just a rescaling factor that fixes where, within the truncated distribution of growth rates, the mean of the excluded species lies. In the SI we justify $x = 0.45$ from the skew of the truncated normal, and we verify it against the simulations, which we now refer to in the main text (L347).

Major comment 2 — Accessible definitions

The authors have been rather sparse with explanations/introductions of ecological and theoretical concepts. Many of the readers of PLOS Computational Biology *might, however, not be familiar with the mathematical and ecological handling of a LV competition or metacommunity model. Below, I have summarised the concepts for which I noticed missing explanations or definitions:*

Response: We address each of the four sub-points below.

2.1 — Weak, diffuse, preemptive competition

The clarity of the main text and S1 would greatly benefit from concise, verbal and mathematical definitions of weak ($a_{ij} < a_{ii} = 1$), diffuse (all $a_{ij} = a$) and preemptive ($a_{ij} = a_{ii} = 1$) interspecific competition at first mention. While the assumption of weak competition is made in the main text (l. 98-99), the assumption of $a_{ii} = 1$ is only stated implicitly, and it is not expressed in the model

equations, starting already with Eq. 1 where it is omitted right away. While this might be common practice with LV competition models, this lowers accessibility for readers from different backgrounds. In S1, the information that $a_{ii} = 1$ is used again in the matrix equations, specifically the elements of the interaction matrix (Eq. S15, S1 l. 70 and subsequently). For completeness, it might thus be good to start the model equations including a_{ii}.

Response: Adopted. In Eq. 1 we now explicitly include the intraspecific term $a_{ii}N_i^{(l)}$ (with $a_{ii} = 1$), and we add definitions at first mention of each competition regime. Note, however, that we did not refer to $a_{ij} < a_{ii}$ as *weak* competition, and that *preemptive* competition corresponds to $a_{ij} \geq a_{ii}$, and not only $a_{ij} > a_{ii}$.

2.2 — Definition of preemptive competition

A definition of the term 'preemptive' competition is necessary at first occurrence, alongside of explanations of why it is necessary to address it in the dataset (see comment 4, l. 152 f) and which case scenario it represents in the model (strong competition, $a_{ij} = a_{ii} = 1$) in a clearer way. Right now, this information is often only implicitly given (cf. l. 108-109).

Response: We added a definition upon first mention in the maintext (L190), and explain why it is sensible to test for it with the *Daphnia* dataset (L226).

2.3 — Feasibility domain

The authors claim to show how multispecies patch occupancy relates to the framework of feasibility (domains) (l. 62-64, 274-76). While they indeed introduce an estimate of the feasibility into their approximation, as a function of n (Eq. 11), they never really explain the concept to the reader (verbally or mathematically), and that and why exactly it can be used here as a replacement for the persistence probability of a species in isolation. In the discussion, they refer to 'feasibility' as 'having a positive pop. density' (l. 341), which seems a bit too general. In S1, the explanations of the feasibility domain are scarce as well, and feasibility is only defined as a function of m.

Response: We have added an explicit explanation of the feasibility domain to the Methods (L161).

2.4 — Terminology

The terms biodiversity, local and regional coexistence, multispecies persistence, co-occurrence and community-level/multispecies patch occupancy are used very interchangeably throughout the manuscript. Clarity of the manuscript would benefit from consistent use of fewer terms and early, concise definitions. More generally, the manuscript mixes patch-dynamics and mass-effect perspectives on metacommunity dynamics and coexistence mechanisms, and this is also reflected in the abovementioned terminology.

Response: We have harmonized terminology. We now use: (i) *local persistence* for a single species

having density above the threshold in a patch; (ii) *community-level patch occupancy* for the fraction of patches where all n regionally available species persist; (iii) *co-occurrence* only for the empirical Daphnia pair-level analysis. We removed occurrences of "biodiversity" when it was used as a synonym of "local richness".

Major comment 3 — The 'regional equivalence' assumption

The 'regional equivalence' of the species is a central assumption throughout the analytical work in this manuscript. In l. 91-96, it is introduced as equal mean of species' growth rates of species across habitats, albeit stochastic differences of $r_i^{(l)}$ across species and local habitats (as proxy for spatial heterogeneity). It would be helpful if the authors could add a few extra sentences to introduce the concept, give an example of how growth rates could be distributed in a small habitat network with hosting a few species (this might be greatly supported by a conceptual figure!), and explain the consequences of this assumption. In S1, the terms used for central assumptions like 'regional equivalence' are not defined in words or equations (e.g. l 53 starts with 'because of regional equivalence' without ever defining what that is). Their use in simplifying the equations is often only described very sparsely, and their implications for growth rates and population densities without dispersal is not explained well (see also other comments).

Response: We have expanded the definition of regional equivalence in both the Methods (L154) and the SI. We also added a small conceptual sketch in the SI that shows how growth rates can vary across patches while obeying regional equivalence (Fig. S1).

Consequence 1 of regional equivalence

Consequence 1 of regional equivalence of the growth rates: regional equivalence of population densities? S1, l. 53f reads 'because of regional equivalence, the first of these sums [the total density of the focal species across all disconnected patches but the focal one] is the same for all species.' This is a nontrivial assumption and not further explained in the following. Why would the stochastic combinations of intrinsic growth rates among patches and species not lead to differing competitive scenarios and thus varying regional densities among species, even under the assumption of diffuse competition?

Response: We clarify that, under regional equivalence *and* diffuse interactions, the equilibrium density of species i in patch l (in absence of dispersal) is a linear function of $r_i^{(l)}$ and of the local mean $\overline{r}_m^{(l)}$. Summing over patches and using that the across-patch means of the $r_i^{(l)}$ are identical among species yields the claimed result. We added this intermediate step to the SI.

Consequence 2 of regional equivalence

Consequence 2: The strong competition scenario ($a \to 1$) implies superiority of any species in only a single habitat patch, while it is outcompeted in all others (S1, l. 148-150): does regional equivalence imply that a species i cannot by chance have the largest intrinsic growth rate $r_i^{(l)}$ in more than one habitat? As long as the regional mean is balanced through other habitats with very low growth rates (drawn from a truncated normal distribution), this scenario should be unlikely, but possible? What about systems with more patches than species? I believe at most in the latter case, this assumption does not hold.

Response: The reviewer is right. The step in the SI that reduces to $\rho_i = 0$ in the strong-competition limit ($a \to 1$) implicitly requires that no species wins (has the largest local $r_i^{(l)}$) in more than one patch — i.e., the local winners are all distinct. With growth rates drawn i.i.d. across patches and species, the probability that the focal species also wins another patch scales as $\sim (p-1)/n$ and is therefore small only when $n \gg p$ (many more regionally available species than patches). We have made this restriction explicit in the SI.

Major comment 4 — Comparability between model and data

The way the analysis of model and data were presented here still made a quite disconnected impression. In the Introduction, comparative analysis of the Daphnid metacommunity is only briefly mentioned at the very end. The clarity of the study would greatly benefit from a statement regarding why this exact dataset is suitable for comparison against the model.

Response: Addressed; see response to Reviewer 1's major comment 3 above. We added a dedicated paragraph to the Introduction listing the features that make this dataset suitable.

Importantly, I noticed a conceptual mismatch between what is assessed from the model and the data. While from the model, the probability for all species to persist in a given local patch is assessed as the final methodological step (community-level patch occupancy); this exact scenario is excluded a-priori from the data analysis of the Daphnid communities since it was so rare ('only 0.33 % of samples, l.148-149), and only the probability and predictors for pair-wise co-occurrence were assessed.

Response: We agree. We now present an additional simulation experiment that more closely resembles the field data ($n = 3$ species), and apply the *pair-level* readout of these simulations (probability that 2 of the 3 species co-occur). Within these new simulations, we also allow p to go up to 100, again matching the data.

Furthermore, the model analysis only treated a case for $n = 10$ species in $p \geq 10$ habitat patches (l. 100f, 107), while the Daphnid metacommunity consisted of 3 species in varying number of habitats. Why was n never varied (and the patch number never decreased for a smaller n) in the analytical considerations or numerical simulations, especially to aid comparison with the data analysis?

Response: See our previous reply.

Lastly, providing some more context on Daphnids (an organism group that might not be familiar to all of the readers) might increase accessibility. Specifically, an extended explanation in the Methods of why pre-emptive competition was assumed/tested and why and how the desiccation rates of the rock pools can be used as proxies for the dispersal rates (with higher desiccation rate = higher dispersal rate?) would be helpful. Concerning the latter: the desiccation rate in the data seems to be a habitat-based characteristic (L. 143-145), but dispersal rates were before estimated from species traits (characteristic distances) and interpatch spatial distance, i.e., relative link length (l. 120 f); how are these two metrics comparable?

Response: We added a paragraph at the start of the *Daphnia* analysis (L224) explaining (i) the life cycle of *Daphnia* (short parthenogenetic generations in summer, resting eggs upon desiccation, wind-driven dispersal between pools), (ii) why preemptive competition is a natural hypothesis, and (iii) why desiccation rate is a reasonable proxy for dispersal frequency (dispersal occurs via resting eggs that leave the pool after desiccation). With respect to the correspondence with the model simulations: please refer to our earlier reply.

Miscellaneous minor comments

l. 37: since immigration from one patch and emigration into another patch are modelled at the same time (instantaneous movement), d does not measure how quickly individuals move among habitats, but rather, how frequently?

Response: Corrected: d now reads "the per-capita *rate* at which individuals leave one patch and enter another".

For completeness, it would be good if the first simplifying assumption(s) made for all of the analytical considerations, the 'well-mixed-assumption', and equal 'characteristic distances' among species, was stated alongside the model introduction (Eq. 1 and S1); After all, these allow the authors to use a single parameter D (or, d) to describe movement of all species among all habitats. Only the Methods for the numerical simulation describe these assumptions as they are relaxed (l. 112-113, 119-123).

Response: Agreed. We introduce the assumptions that are inherent for Eq. 1 on L72, and the two assumptions we make to analyse Eq. 1 starting L151.

l. 99-100, 103-104 consider shifting the information in S1.2.3 to the main text.

Response: Including the full algorithmic details in the main text makes the article harder to read and we would therefore like to keep it in the SI.

l. 106 f: For the relaxed assumptions, simulations had to be run with an extended version of the model described by Eq. 1, for example for the more complex dispersal structure; for completeness,

the Methods or Supplementary should state the exact model used for simulations.

Response: We agree and have expanded the existing "Simulation model" section in the SI to specify the equation and parameter setting.

l. 196: Eq. 7 does not calculate probabilities; consider rephrasing this part or generally explaining, how this shift from calculating densities to probabilities is made.

Response: Rephrased.

l. 198–202, also 210f: the effect of the spatial network size (p) is not a factor in Eq. 7, but only in the sum of the numerator, thus only indirectly affecting a species' local density by increasing its regional density in isolation. Whether this is the case should depend on how likely it is that a species can persist in more patches when the network is larger – and this again depends on the interaction strength (see limiting cases considered in S1, l.145-150). The relation thus seems less straightforward than noted here and should be at least explained in more detail.

Response: The limiting cases refer to the *persistence without dispersal scenario*, and is thus disconnected from the *extinction without dispersal scenario*. We hope that the restructured text avoids this misunderstanding.

Figure 1: The figure should be understandable without the main text or SI. Please add that these are analytically estimated values and note the equations and constraining assumptions this is based on. Consistently define parameters and indices in the legend, e.g., that a refers to interspecies interaction strength, and that N is given for a species i in patch l.

Response: Fig. 1 caption rewritten accordingly.

L. 234: specify that it still is the community-level patch occupancy that is calculated; From which equations and using which methods is this done? Especially the estimation of feasibility domains?

Response: Clarified starting on L381 and in the caption of Fig 2. The feasibility domain is computed as described in the supplements (referred to in the methods on L170).

Figure 2: why is the community-level patch occupancy so sharply increasing (almost binary from 0 to 1?) around $d = 10^{-4}$*? Could we see an additional panel for* $a = 1.0$*? Legend reads "values up to 0.005 are weak"; of what parameter?*

Response: The sharp transition occurs because of the wide range of d. Adding a panel for $a = 1.0$ is not possible because our model analysis only holds when $a < 1$. Fig3 contains the simulations for $a = 1$.

Figure 3: Legend: what does 'Dispersal is no longer implicit' mean? Also, missing 'no' in the row description.

Response: Rewrote the legend.

L. 248–250: this result could use a figure reference.

Response: Added reference.

L. 258–260: Equation or figure reference for the regression model and its results?

Response: Added references to the equation and to the main result (figure). The factor 1000

comes from the increase of the log odds-ratio of about 7, which we clarified in the text (L429).

L. 263: can this value be seen in a figure? In Fig. 5 it looks like this value should be lower.

Response: Agreed, this was an error. Corrected

Figure 4: please revise the axis labels and legends to display complete information: from which equation/model was this assessed, what is the tested response variable, what is 'focal' and 'value', what effects are fixed and random.

Response: Done.

L. 262–268 Why are the posterior distributions of predictors from the Daphnid co-occurrence model (Eq. 5) not shown in a figure in the main text?

Response: These are now added to the main text.

L. 277: microcrustacean is used as a descriptor for Daphnid communities for the first time here.

Response: "Microcrustacean" is now introduced at first mention of Daphnia in the Introduction.

L. 285: should read 'exclusion rate' instead?

Response: Yes, corrected. We now consistently call the denominator of Eq. 7 the *exclusion rate*.

L. 285–86: this information is quite disconnected here and I believe it contradicts the model assumptions of this study. In l. 189 and also the SI it is stated that this denominator is always positive and, most importantly, never zero. Also, in Eq. S23, it is assumed that for non-persisting species, intraspecific growth is always lower than interspecific competition. If needed, this information might fit better in the Methods.

Response: On the first part: there is no contradiction here. The denominator is always positive because it is the negative of the invasion growth rate, which is itself negative by construction in the "exclusion-without-dispersal" case. We improved the wording and the intended ecological interpretation. On the second part: nowhere in the analyses of the model do we assume that intraspecific competition is weaker than interspecific competition. Eq. S23 does not say this either. It only says that the intrinsic growth rate is smaller than the total amount of interspecific competition.

L. 288: if the increase in regional density comes from dispersal alone, where do the individuals come from? Cf. Equation also S30 and comment on S1 below.

Response: They come from the $p-1$ other patches, each of which donates a fraction d of its equilibrium density per unit time. We have added an explanatory sentence to make this bookkeeping explicit (L328).

L. 291–96: is this effect observed because the model is designed in a way that a larger network size makes local persistence more likely by chance and thus regional density stochastically increases? Intuitively, this indeed reads like stochastically assigning larger realised niches to species. Please clarify.

Response: We explicitly clarify that in our model the additional patches contribute to the regional-density term of the former Eq. 7, which is what makes local persistence more likely. We updated

the text accordingly (L336). There is no link to stachasticity.

L. 302–03. I believe this is only true under the 'well-mixed' assumption, where new patches will be connected to all other existing patches.

Response: Our simulations show that using more complex dispersal mechanisms does not change this result.

L. 303–307: already existing community-level extensions of metapopulation models often clearly distinguish between stochastic extinctions from the environment and exclusion due to interspecific competition. I would be careful to mix these two concepts together here.

Response: This section does not discuss about stochasticity. We're not sure how to best interpret this question.

L. 320: 'to the same Daphnia data' – as the dataset analysed here?

Response: Yes; clarified.

l. 326–333: Desiccation rates did not affect co-occurrence: remind the reader that this is a proxy for the dispersal rate – this result is not contextualised here.

Response: Added a parenthetical reminder on L436.

L. 330: the effect of dispersal on what?

Response: Clarified.

L. 342–344: please define what is meant by 'stability' (temporal, dynamical?).

Response: Clarified as *local asymptotic stability.*

L.351: please consider rephrasing, there was no (mathematical) proof involved in this analysis.

Response: "Prove" replaced by "show analytically".

L. 366–67: where was this shown in the results (figure reference)? The effect of a higher/lower growth rate was not mentioned before?

Response: This is a speculation – we reworded accordingly (L573).

Comments on the Supplementary Material

Generally, S1 is written very densely and often short of explanations of 'why' a specific assumption is made or what this implies for an equation. For example, l. 125 reads that 'the per capita growth rate at equilibrium is zero'. The consequence of this is that one can drop the first term of Eq. 9; this is not verbally noted, and in the first line of Eq. 28 executed together with the consequences of the regional equivalence and diffuse interaction assumptions. Expressions from previous equations are inserted without clear references (e.g. that Eq. S12 inserts Eq. S11 into Eq. S4, or that Eq. S26 is derived from Eq. S14 and Eq. S22) or assumptions are transferred from the main text. While this all not invalidates the methods presented here, there is a lot of methodological expertise required from the reader and capability to keep track of numerous details mentioned elsewhere to follow this analysis.

Response: We rewrote the SI to be self-contained: each derivation now states which equations are being combined and which assumptions are being invoked, and the consequences of 'growth rate at equilibrium is zero', regional equivalence and diffuse interactions are now spelled out verbally

where they enter. See also our responses to the specific points below.

L. 24: the weak d assumption is only needed in the next step (for truncation of the power series beyond linear terms).

Response: Corrected. We now state that $0 \le d \ll 1$ is required only for the truncation beyond linear terms.

L. 50–56: Please revise this paragraph (1.2.2.), it is hard to follow these assumptions, the conclusions drawn from them and why the next steps are necessary.

Response: Paragraph rewritten for clarity.

Eq. S13: should the left side of this equation not also have a second sum over all species, and then divide it by n? cf. l. 54–55. If this expression can immediately be simplified, please verbally explain why.

Response: As specified in the text preceding this equation, this sum is the same for all species, under the assumptions listed. Rephrased for clarity.

Eqs. 16–17: please check the notation of the row- and column vectors. The current notation implies $\mathbf{u} = \mathbf{v}^T$, which then are multiplied in Eq. 16.

Response: Corrected: $\mathbf{u}$ is now defined as a column vector, and $\mathbf{v} = \mathbf{u}^\top$ as a row vector.

Eq. 19–20: the elements of the inverse of the interaction matrix have the same notation as the original interaction coefficients; this is very hard to read; consider using a different letter.

Response: We now denote the elements of $\mathbf{A}^{-1}$ by α_{ij} instead of $(a_{ij})^{-1}$.

SI L. 61–62: please explain the 'feasibility domain size', and introduce the 'diffuse interaction' assumption, perhaps even with an equation statement such as $a = a_{ij} < a_{ii} = 1$.

Response: Done; see our response to Major comment 2.3.

Eq. S15: to be able to understand why the diagonal elements of $\mathbf{A}$ are $= 1$, the reader needs to know how an interaction matrix is built, specifically, that this one is of size $m \times m$, that the intraspecific interaction coefficients a_{ii} are on the diagonal; and lastly, that $a_{ii} = 1$ and therefore it was omitted in Eqs. S1 through S6.

Response: Added a sentence introducing the structure of $\mathbf{A}$ ($m \times m$, 1's on the diagonal, a's off-diagonal).

L. 75f and Eq. 21: consider reminding the reader that Eq. 19 and 20 are inserted into equation 6, and that all this assumes there is no dispersal; why does r_j in the first line have no patch index?

Response: We added the reminder and corrected the missing index.

L. 89–102: inconsistent use of patch-index (l) in $\overline{r}_m$ notation? If it is intentional, where is the difference between the two?

Response: We made the notation consistent: mean growth rates now always have a superscript denoting the patch.

SI, L. 121–22: 'We then sample $r_i^{(l)}$ from the distribution of growth rates $g^{(l)}$ (truncated normal, given in the main text)' – this relies too much in information presented in the main text.

Response: We now state the distribution explicitly in the SI (standard normal truncated to the

positive numbers, rescaled so that the across-species mean within a patch and the across-patch mean for any species both equal 1).

Figures of supplementary material S1 have no numbering or description. For example, it is unclear whether the first of these figures presents simulated and or/ calculated data points from Eq. 25.

Response: All SI figures now have numbers and are in a separate section (S1, S2, ...) and have self-contained captions.

L. 135: I do not see where this is explained in section 1.2.1.

Response: Section reference corrected.

L. 136–140, Eq. 29: I think this paragraph follows the same steps as l.65-75, please remind the reader of that, and also, what the goal of dealing with this matrix equation is (to solve for $\hat{N}_{1,i}^{(l)}$). As it is, it is very hard to follow.

Response: Added a short introductory sentence.

L. 142 and Eq. 30: It would be good to remind the reader that this is a linear approximation (building on Eq. 4). If $n = m$, the first term inside the bracket of the numerator can become $= 0$.

Response: We consistently checked if this was explicit throughout and changed when it was not.

The paragraph (l. 145–159) argues for why the second term of Eq. 30 should be positive or zero for all parameter combinations, when considering two extreme cases of strong and weak interactions. In the strong competition case, the assumptions imply that $\hat{N}_i^{(l)} \geq \hat{N}_{0,i}^{(l)}$. If this holds for any species i on any patch l, that means that dispersal is creating individuals to add to a population out of nowhere. It would be interesting to see if this also happens for the intermediate interaction parameters a, from the actual approximated populations densities with and without dispersal, instead of just the probability of persistence (Figure below l. 159).

Response: Strong competition will often imply only one species is present per patch *when there is no dispersal*. The term with $N_{1,i}$ is not the amount that is physically added from other patches, but rather a correction term.

Reviewer #3

In this paper the authors provide a theoretical explanation for the classical result in metacommunity ecology that weak dispersal amongst habitat patches enhances local diversity. The authors start from a classical model equation to investigate the mixed effects of species interactions, intrinsic growth rates and dispersal between discrete patches on multispecies communities' population dynamics: the competitive Lotka-Volterra with dispersal terms. They then limit themselves to the cases in which dispersal is weak, interspecific interactions are weak (compared to intraspecific) and diffuse (i.e. no structure, all species compete equally likely), and growth rates are heterogenous among species across the landscapes but at the landscape scale they are the same across all species (i.e. normalised). Another important assumption is that there is no connectivity structure across the landscape: i.e. all patches are equally connected to all others (they later relax this assumption by introducing a dispersal kernel). These assumptions allow them to derive expressions for the probabilities of encountering (1) a given species and (2) all the species at once in a randomly given patch: i.e. patch occupancy. Analytical results for these probabilities and the conditions for persistence that ensue are then complemented with numerical simulations matching the predictions. The authors further analyse empirical data from a metacommunity consisting of 3 species of Daphnia distributed amongst rock pools in a Finnish archipelago. Even though the empirical data is not completely ideal to test all the aspects of the theory (but I guess no dataset ever is!), the data illustrates nicely one of the main focus of the theory: larger landscapes (in the form of larger number of nearby rock pools) increase the probability of coexistence of pairs of species. The paper is nicely written, and I think provide a nice contribution to theoretical ecology. The authors have taken good care of explaining the rationale for the derivations clearly (although see my specific comments below for suggestions on a few places where this could be improved), which makes the paper very clear.

Response: We thank the reviewer for the positive evaluation and for the constructive, specific comments.

Main concern — Availability of code and data

My only main concern is that the source code and data for the analyses was not provided, which first of all prevented me from being able to assess the validity of the results presented, but more importantly goes against the open science and source code as well as replicability that I believe we all as scientists should strive for. I wouldn't recommend this paper for publication unless this is addressed and definitely before any decisions are made the code needs to be verified. The source code presented should contain all the instructions to be able to re-create all results and figures in the paper and should be auto-contained and easily runnable on any platform.

Response: Data and code were already available in the previous submission, as specified in the Open research statement: Data and computer code are available online at `https://doi.org/10.5281/zenodo.15311497` and `https://github.com/fdelaend/occupancy`.

Below I also detail a few parts where I think some clarifications would improve the quality of

the paper.

Response: We address each specific comment in turn below.

Specific comments

Line 54–55. It would be nice to have a quick explanation of why these considerations make the analysis of Eq. 1 possible. Just to guide the reader a bit more and let them know what to expect.

Response: Added an explanation on L94.

Line 80. A linear function of what? N_i is already a linear function of D anyway in Eq. 1.

Response: Of d. Clarified.

Line 83. In expression $N_i = N_{0,i} + dN_{1,i}$ it is not clear why the second term $dN_{1,i}$ means it is dispersal to the same patch itself? And why doesn't depend on the other patches as in Eq. 1. I am guessing it is because you are assuming D to be close to 0 but this could be better explained

Response: We now explain: $d\hat{N}_{1,i}^{(l)}$ is the first-order (in d) correction to the equilibrium density in patch l; it does not refer to dispersal to the same patch, but to the sensitivity of the density to small dispersal. We added this clarification (L133).

Lines 138–139. How many freshwater rock pools? And how many islands?

Response: Added the numbers.

Line 140. It would be nice to have a figure showing the clustering of the seven group of islands and maybe a map of where they are to give the reader a better sense of the data.

Response: A map with the seven clusters is now included in the supplements as Fig. S2.

Line 157–158 is repetitive with lines 152–253.

Response: Redundant sentence deleted.

Line 234. The "we can compute patch occupancy analytically" is a bit of a leap of faith here. This would benefit from a more specific explanation. How can you compute the occupancy analytically?

Response: Addressed. We now state explicitly how patch occupancy was computed on L392.

Line 255. This subsection of the results could start acknowledging the shift of focus to the empirical system and presenting a sentence or two re-introducing the empirical question. To make the flow more readable.

Response: Transitory paragraph added on L420.

Line 285. "the extinction rate can be negative" is confusing. Technically speaking I guess a "negative extinction rate" would imply the spontaneous creation of species, but in any case I don't think this is what you are trying to argue here since this would mean the local conditions are super suitable. So it would be good to re-write this to avoid confusion.

Response: Rewritten. We now speak of "the exclusion rate" and no longer describe it as potentially negative.

Line 330. Unimodal rather than unimodel.

Response: Corrected.

Figure 1 caption. What about the r's? Don't densities also depend on the values of r?

Response: The values of the r's are rescaled to have a mean of 1. Added to the caption of Fig. 1.

Figure 2 caption. "suggests values up until = 0.005 are weak." Values of what? Of d or a? It sounds like it should be d, but this is said just after discussing a, which makes it confusing.

Response: Values of d. Caption updated.

Caption figure 3. The second appearance of "regional equivalence" (referring to the bottom row) should say "regional inequivalence".

Response: Corrected.

Figure 4. The colours are confusing and not easy to differentiate, especially the 2 pinks, but also the blue and the green.

Response: We redesigned the palette to a colourblind-friendly scheme.

Figure 4 caption. The title would benefit from more detail: species of what? Where? In which context? It would also be good to give a sense for what a negative intercept means here.

Response: Caption rewritten accordingly; the intercept is interpreted as the baseline log-odds.

The thick intervals are defined but the think intervals have been left unexplained. Please correct.

Response: Done: thick intervals are 66% credible intervals, thin intervals are 95% credible intervals.
