# Supplementary material for: Weak dispersal and landscape size inevitably promote local biodiversity in heterogeneous metacommunities of competing species
Source: PLoS Comput Biol. 2026 Oct 16;22(10):e1014028. doi: 10.1371/journal.pcbi.1014028 (PMC13705597; doi:10.1371/journal.pcbi.1014028)
Supplement: Supplementary file 4 [file pcbi.1014028.s005.pdf]

Response to reviewers — Manuscript PCOMPBIOL-D-26-00366

"Weak dispersal and landscape size inevitably promote local biodiversity in heterogeneous metacommunities of competing species"

Frederik De Laender, Andrew Gonzalez, Olivia Bleeckx,
Dieter Ebert, György Barabás

September 1, 2026

Dear Dr. Martinez-Garcia,

We thank you and the reviewers for the thoughtful evaluation of our manuscript. The comments have substantially helped us improve the manuscript. Below we address every point raised by each reviewer. The reviewers' comments are reproduced *in italics*, followed by our **Response:** response in blue. We are uploading the following files:

- This rebuttal: Rebuttal.pdf.
- The revised manuscript with track changes in the originally submitted format: Main-r2-track-changes.pdf. *Note: The line numbers in the rebuttal refer to the line numbers in that marked-up version.*
- The revised manuscript in the PLOS format: Main-r2.pdf.
- The LaTeX Source File using the PLOS template: Main-r2-PLOS.tex.
- The revised Supporting Information: Supplements-r2.pdf.

We hope that the revised manuscript now meets the standards of *PLOS Computational Biology* and thank again the editor and reviewers for their time.

Sincerely,
Frederik De Laender, on behalf of all co-authors.

Reviewer #1

The authors have carefully revised the manuscript and addressed all of my previous concerns in a thorough and thoughtful manner. I have no further substantive comments. My only suggestion is that the limitation statement, currently rather exhaustive, could be condensed to enhance clarity and flow, as it tends to distract from the main conclusions. With this minor revision, I would be happy to recommend acceptance.

Response: We thank the reviewer for this positive assessment. In response, we have considerably shortened the sections of the Discussion addressing limitations and future directions.(L 528–601).

Reviewer #2

I appreciate the authors' extensive revision of this manuscript and the supporting information. The current manuscript version poses a substantial structural improvement compared to the original submission and now provides clear definitions for ecological concepts as well as model parameters. Therefore, most of the issues I had previously commented on have been resolved.

Response: We thank the reviewer for this constructive feedback.

To connect the analytical considerations better to the analysis of the dataset of a natural Daphnia metacommunity (responding to my previous major comment 4 and a major comment of Reviewer 1), the authors have added additional model simulations performed and analysed for parameters representative of the Daphnid rock pool metacommunity. This much improved the integration of the empirical system into the overall study design, but nonetheless, the general fit of the dataset to the LV model was addressed only partially. Specifically, the authors argue that the Daphnid dataset is well suited to their modelling framework (starting in l. 63 of the typeset article), i.e., the LV-competition model in a network-like landscape, mainly focusing on the spatial aspects: because it is a naturally patchy and environmentally heterogeneous habitat network linked by passive dispersal and thus forms a metacommunity. While I agree with this argument, to me, the local interactions incorporated into the analysis and simulations of the LV-model and the dataset on the Daphnia metacommunity seem quite contradictory. Illustrative for this issue, the authors write in the last part of the introduction, only a few sentences apart that "conclusions continue to qualitatively hold when relaxing our analyses' underlying assumptions, unless interspecific interactions are strong (l. 58-59) and then in l. (67-68) that "Our analysis of these data suggests strong interspecific interactions". Therefore, I'm still not convinced that the dataset is useful to confirm findings from the analysis of the LV model.

Response: We agree that this wording could appear contradictory, and we thank the reviewer for drawing our attention to this issue. The apparent inconsistency resulted from our original statement that our conclusions hold "unless interspecific interactions are strong," without making clear which conclusion this qualification referred to. We have now clarified that strong interactions specifically complicate the relationship between dispersal and coexistence, which becomes unimodal, whereas the positive effect of landscape size remains robust across interaction strengths. We have added this clarification immediately following the relevant caveat in the Introduction (L 61-64). This distinction also explains why the *Daphnia* dataset, in which we detect strong interspecific interactions, provides a valid and informative test of our theory. Specifically, it allows us to test the prediction concerning landscape size, which our theory shows to hold regardless of interaction strength, rather than the prediction concerning dispersal, which is altered under strong interactions. Accordingly, we have also revised the sentence describing our empirical findings to make explicit that species co-occurrence was unrelated to our proxy for dispersal but increased with the number of neighboring pools surrounding a focal pool, in line with the theoretical prediction for the strong-interaction regime. (L 70-73).

The second aspect is the actual assessment of this strong interaction, i.e., the pre-emptive com-

petition in the dataset: It does not become clear why the authors chose to analyse the data set for prevalence of pre-emptive competition as a first objective. Conceptually, I would guess that it makes sense to confirm the assumption made in previous studies (cf. l. 215-218) before parameterising the LV model for simulations with $a \geq 1$. However, this or any other reasoning behind the initial data analysis is not spelled out in the Introduction or Methods.

Response: We first tested for preemptive competition directly in our dataset because establishing its presence in this particular system, rather than inferring it from previous studies of other *Daphnia* systems, informs the parameterization of our subsequent system-specific simulations (Methods). In these simulations, we vary $\overline{a}$ between 1 and 1.4 to reflect the expectation of preemptive competition in this metacommunity. We have revised the Methods to make this rationale explicit and to clarify that, although preemptive competition has been documented in other *Daphnia* systems, it had not previously been demonstrated for the Tvärminne metacommunity itself (L 197–199 and L 211–214).

Lastly, it would be helpful to contextualise the empirical system within the model framework used for the analytical considerations. Due to the pre-emptive competition, the Daphnia metacommunity should only correspond to one of the two treated cases of the analysis: the case where the probability for coexistence is estimated for communities that do not coexist in the absence of dispersal, right? The Daphnia dataset thus seems to only represent an edge case of the possible interactions considered in the rest of the study. If I didn't catch a wrong train of thought here, this should at least be mentioned in the methods or discussed after the results.

Response: Our analytical results assume diffuse interspecific interactions that are weaker than intraspecific interactions ($a < 1$), which excludes the empirical system. A main conclusion of this analysis is that dispersal and landscape size promote biodiversity. Our simulations, which do hold for $a \geq 1$, and therefore cover the empirical system, confirm the landscape size effect, while showing more variable effects of dispersal. The empirical system is therefore not directly comparable to the model analyses, but does support the landscape size effect coming out of our analysis *and* simulations. We clarify this in a new paragraph in the discussion, on L 501–510.

To summarise, in my opinion, the question of whether the model is able to predict the pairwise coexistence of the Daphnia species in the rockpool metacommunity because or despite of pre-emptive competition still needs to be addressed.

Response: We hope our revised version addresses this question.

Otherwise, I have some minor comments on the text and figures: General comment on Figures: I noticed that all figures currently displayed in the main text show Results, while Methods-related Figures remain in the Supplementary information where most likely, much fewer readers will see them. The original request for conceptual figures was however made to increase accessibility of the methods, i.e. illustrate the Daphnia data and the model assumptions, for the readers. Therefore, I wonder whether it would be possible for the authors to join some of their results figures currently displaying related results (e.g., Figs. 2 and 3 or 4 and 5) to make some space for the Daphnia data map (currently Figure S2) and/or the conceptual figure (currently Figure S1) in the main text

Methods?

Response: We have combined the former Fig. 2 and 3 into a single figure, now Fig. 3. We have moved Fig. S1 to the main text. We renumbered all figures.

L. 45: consider rephrasing, since the weak dispersal assumption does not allow the power series expansion, but rather its truncation after the linear term – which then again makes the expression analytically solvable (cf. also l. 48 in Supporting Information, S1.1)

Response: Agreed and rephrased on L 45-46.

L. 67: consider defining landscape size as the number of habitat patches at first use; especially in combination with 'local', it could be mistaken for 'rock pool'/'patch' size; or the overall extent of the landscape in which the habitat patches are embedded (which is not considered here)

Response: Agreed and defined upon first use in the abstract, introduction, methods, and the results.

L. 78-82: Again, I believe the most vital point of the approximation is only implicitly stated: assuming weak dispersal allows you to cut off any nonlinear correction terms; i.e., the weak dispersal assumption implies that only a linear correction term needs to be considered, and any higher order terms are negligible. Please consider adding this implication here.

Response: Agreed and adapted on L 82-83.

L. 92: The mention of "rescue" in brackets seems a bit out of context here – if you want to use it to illustrate the scenario you are considering in this paragraph, please explain the concept in more detail.

Response: We agree it was out of context, and possibly confusing. Removed from the text.

L. 112: While I appreciate the clear ecological definition of the feasibility domain in l. 110-111, I wonder whether the technical information given subsequently is necessary/relevant for the further considerations in this study?

Response: We have added this explanation in response to a comment on the first version of the manuscript so we would prefer keeping it in the manuscript.

L. 124-153: I have the impression that these paragraphs could be written more concisely – for example by integrating the statement starting in l. 148 where you refer to the strict assumptions case into the first paragraph of the simulations section (starting in l. 124), where you explain how you relax these assumptions.

Response: Agreed. This whole paragraph was rewritten (L 129–190).

L. 129: I wasn't sure how the parameter a introduced here relates to the previously introduced interaction parameters $a_{ij} = a$ or a_{ii}.

Response: Clarified as $\overline{a} = 1/n \sum_{j\neq i}^{n} a_{ij}$ on L 138.

L. 130-131: is this displaying the same information twice? Otherwise, please consider rephrasing to differentiate these cases better

Response: This whole paragraph was rewritten to improve clarity.

L. 138: please specify: intra- and/or interspecific interactions?

Response: We mean interpecific. Specified.

L. 146: please define the parameter c

Response: Was already defined on L164.

Computer Code and data (l. 233): in reference to the other reviews of this paper, you may consider re-iterating the availability of your computer code and data here?

Response: Done.

L. 279: consider reminding the reader how you define landscape size (see comment above), since it becomes important here

Response: Done on L. 24, L. 302–303, and L. 310–311.

L. 301: if I read the equation correctly, 'regional density' is not precise here, since the focal patch l is excluded from the sum in the numerator. Instead, you may stick to the terminology used to describe the numerator of Eq. 8

Response: Agreed and adjusted throughout the manuscript.

L. 304: additional assumption for the conclusion from the limiting case a -> 1 is n»p. This restriction should be mentioned here for completeness.

Response: Done on L 336-337.

L. 305: of the parameter a?

Response: Yes. Completed on L337.

L. 305: 'this conclusion' actually, the conclusion/consequence of the first order correction being positive is not explicitly stated here: if this term is positive, then the equilibrium density with dispersal is always larger than the equilibrium density without dispersal, no?

Response: Yes. Clarified on L 338-339.

L. 318- 319: this is repetitive to the statement in l. 311, consider merging them

Response: We cannot really merge these two sentences because we need this result in the two distinct parts of this section.

L. 343: intra- or interspecific competition?

Response: Interspecific. Specified on L 391.

L. 394: consider using 'analyse' instead of 'present', since this data set has been published elsewhere before?

Response: Agreed. Changed on L 443.

l. 438-439-: Personally, I would define mass effects even more broadly, as dispersal of individuals in such large quantities that they are able to affect local population sizes (regardless of the viability of a local population due to purely local interactions and environment). What is described starting in l. 438 I would call a rescue effect, and thus only a special case of mass effect dynamics, where dispersal allows persistence of an otherwise non-viable population. Mass effects often jointly work with local dynamics, and seldomly overrule them completely. Please consider rephrasing this for clarity!

Response: Agreed. Clarified on L 489-500.

L. 443-444: in l. 443, you say that dispersal needs to be sufficiently high, in the next sentence, you say that dispersal needs not to be high. I think I understand what you mean here but you may

consider rephrasing for clarity.

Response: This paragraph has been rewritten following an earlier comment.

L. 467-469: I believe this statement of the discussion could use a literature reference

Response: This paragraph has been rewritten - the statement is no longer there.

L. 510: add 'is' after 'what'

Response: This paragraph has been rewritten.

Reviewer #3

The authors have addressed all my main comments and I am happy with that. I would however recommend that the authors put a little more effort into developing a more informative readme file for the repository with specific instructions on how to run the code and reproduce exactly the results from the paper. It would be useful to have a set of tutorial-like instructions to do this and an automated pipeline that reproduces a basic version of the results. This will improve reproducibility of the results presented in the paper and would also make life easier for those wanting to use this code, thus improving open code and data practices.

Response: We agree and made an extensive readme file.
