# Supplementary material for: Weak dispersal and landscape size inevitably promote local biodiversity in heterogeneous metacommunities of competing species
Source: PLoS Comput Biol. 2026 Oct 16;22(10):e1014028. doi: 10.1371/journal.pcbi.1014028 (PMC13705597; doi:10.1371/journal.pcbi.1014028)
Supplement: Peer review history

# Peer review: Decision Letter 0

**DOI:** 10.1371/journal.pcbi.1014028.r001

**Authors:** Zhaolei Zhang, Ricardo Martinez-Garcia

16 Apr 2026

Weak dispersal and landscape size inevitably promote local biodiversity in heterogeneous metacommunities of competing species

PLOS Computational Biology

Dear Dr. De Laender,

Thank you for submitting your manuscript to PLOS Computational Biology. After careful consideration, we feel that it has merit but does not fully meet PLOS Computational Biology's publication criteria as it currently stands. Therefore, we invite you to submit a revised version of the manuscript that addresses the points raised during the review process.

Please submit your revised manuscript by Jun 16 2026 11:59PM. If you will need more time than this to complete your revisions, please reply to this message or contact the journal office at ploscompbiol@plos.org. When you're ready to submit your revision, log on to https://www.editorialmanager.com/pcompbiol/ and select the 'Submissions Needing Revision' folder to locate your manuscript file.

Please include the following items when submitting your revised manuscript:

* A letter that responds to each point raised by the editor and reviewer(s). You should upload this letter as a separate file labeled 'Response to Reviewers'. This file does not need to include responses to formatting updates and technical items listed in the 'Journal Requirements' section below.

* A marked-up copy of your manuscript that highlights changes made to the original version. You should upload this as a separate file labeled 'Revised Manuscript with Track Changes'.

* An unmarked version of your revised paper without tracked changes. You should upload this as a separate file labeled 'Manuscript'.

If you would like to make changes to your financial disclosure, competing interests statement, or data availability statement, please make these updates within the submission form at the time of resubmission. Guidelines for resubmitting your figure files are available below the reviewer comments at the end of this letter

We look forward to receiving your revised manuscript.

Kind regards,

Ricardo Martinez-Garcia

Academic Editor

PLOS Computational Biology

Zhaolei Zhang

Section Editor

PLOS Computational Biology

**Journal Requirements:**

If the reviewer comments include a recommendation to cite specific previously published works, please review and evaluate these publications to determine whether they are relevant and should be cited. There is no requirement to cite these works unless the editor has indicated otherwise.

1) Please ensure that the CRediT author contributions listed for every co-author are completed accurately and in full.

At this stage, the following Authors/Authors require contributions: Frederik De Laender, György Barabás, Olivia Bleeckx, Dieter Ebert, and Andrew Gonzalez. Please ensure that the full contributions of each author are acknowledged in the "Add/Edit/Remove Authors" section of our submission form.

The list of CRediT author contributions may be found here: https://journals.plos.org/ploscompbiol/s/authorship#loc-author-contributions

2) We ask that a manuscript source file is provided at Revision. Please upload your manuscript file as a .doc, .docx, .rtf or .tex. If you are providing a .tex file, please upload it under the item type u2018LaTeX Source Fileu2019 and leave your .pdf version as the item type u2018Manuscriptu2019.

3) Please provide an Author Summary. This should appear in your manuscript between the Abstract (if applicable) and the Introduction, and should be 150-200 words long. The aim should be to make your findings accessible to a wide audience that includes both scientists and non-scientists. Sample summaries can be found on our website under Submission Guidelines:

https://journals.plos.org/ploscompbiol/s/submission-guidelines#loc-parts-of-a-submission

4) Please upload all main figures as separate Figure files in .tif or .eps format. For more information about how to convert and format your figure files please see our guidelines:

https://journals.plos.org/ploscompbiol/s/figures

5) We have noticed that you have uploaded Supporting Information files, but you have not included a list of legends. Please add a full list of legends for your Supporting Information files after the references list.

6) Please amend your detailed Financial Disclosure statement. This is published with the article. It must therefore be completed in full sentences and contain the exact wording you wish to be published.

1) State the initials, alongside each funding source, of each author to receive each grant. For example: "This work was supported by the National Institutes of Health (####### to AM; ###### to CJ) and the National Science Foundation (###### to AM)."

2) State what role the funders took in the study. If the funders had no role in your study, please state: "The funders had no role in study design, data collection and analysis, decision to publish, or preparation of the manuscript."

3) If any authors received a salary from any of your funders, please state which authors and which funders..

If you did not receive any funding for this study, please simply state: u201cThe authors received no specific funding for this work.u201d

7) Your current Financial Disclosure states, "Yes ↳ Please add funding details. This researchused resources of the "Plateforme Technologique de Calcul Intensif (PTCI)" (http://www.ptci.unamur.be) located at the University of Namur, Belgium, which is supported by the FNRS-FRFC, the Walloon Region, and the University of Namur (Conventions No. 2.5020.11, GEQU.G006.15, 1610468, RW/GEQ2016 et U.G011.22). ↳ Please select the country of your main research funder (please select carefully as in some cases this is used in fee calculation). BELGIUM - BE".

However, your funding information on the submission form indicates recieving no funds.

Please indicate by return email the full and correct funding information for your study and confirm the order in which funding contributions should appear. Please be sure to indicate whether the funders played any role in the study design, data collection and analysis, decision to publish, or preparation of the manuscript.

**Reviewers' comments:**

Reviewer's Responses to Questions

**Comments to the Authors:**

**Please note here if the review is uploaded as an attachment.**

Reviewer #1: The maintenance of biodiversity is a central question in ecology, and understanding how spatial dynamics shape species coexistence is critical for predicting biodiversity responses to global change. Previous work has extensively explored dispersal effects in metacommunities, often through simulation-based approaches. Going beyond, this study attempts to advance our understanding by combining analytical investigations with empirical data. Their analyses showed that weak dispersal and smaller landscape sizes consistently increased local species richness. I appreciate the clarity of the writing and the ambition of the research objective. That said, I have a number of concerns regarding the implications and scope of the analytical results.

First, the paper aims to “explain the well-documented positive effect of weak dispersal on local species persistence in heterogeneous landscapes” (lines 53–54). I was somewhat surprised by this framing, as this pattern seems almost definitional: under weak dispersal, the regional species pool can contribute to local diversity without substantially altering local species interactions or causing competitive exclusion. While the analytical derivations (e.g., Equations 6, 8–10) certainly formalize this intuition, they appear to restate it in probabilistic terms rather than offering new biological insight. As a result, many of the predictions derived from these equations read as expected outcomes (e.g., lines 189–191, 210–212, 215–216, 284–287, 323–324). An exception is Equation 7, which usefully quantifies how local population size depends on dispersal relative to demographic rates and interspecific interactions. However, even here, the qualitative insights remain broadly consistent with existing expectations.

Second, the analyses were built on limiting-case assumptions in which dispersal was weak enough to be treated as a minor perturbation. In this regime, total population size remains effectively unchanged, and dispersal primarily influences spatial distribution. While this simplification aids tractability, realistic scenarios usually involve dispersal rates that considerably alter both population sizes and spatial patterns through feedbacks with local dynamics. Capturing such effects would require models that more explicitly integrate local and spatial processes, though I recognize that analytical tractability is not always guaranteed in such cases.

In addition, the role of the empirical analyses within the broader study framework could be articulated more clearly. As presented, it is difficult to see how the empirical results align with or validate the analytical findings, which weakens the integration between these two components.

Minor comment:

L251: The ecological relevance of the value 0.01 is unclear. Is a dispersal rate of this magnitude ecologically realistic as a representation of 'sufficiently strong' dispersal?

Figure 1: I was unsure whether the probability shown is evaluated against a threshold or under stochastic environments; otherwise, it is not clear why this value would not always equal 1, as suggested in the main text (lines 189–191).

Reviewer #2: see attached file.

Reviewer #3: Review of the paper: “Weak dispersal and landscape size inevitably promote local biodiversity in heterogeneous metacommunities of competing species”

In this paper the authors provide a theoretical explanation for the classical result in metacommunity ecology that weak dispersal amongst habitat patches enhances local diversity. The authors start from a classical model equation to investigate the mixed effects of species interactions, intrinsic growth rates and dispersal between discrete patches on multispecies communities’ population dynamics: the competitive Lotka-Volterra with dispersal terms. They then limit themselves to the cases in which dispersal is week, interspecific interactions are weak (compared to intraspecific) and diffuse (i.e. no structure, all species compete equally likely), and growth rates are heterogenous among species across the landscapes but at the landscape scale they are the same across all species (i.e. normalised). Another important assumption is that there is no connectivity structure across the landscape: i.e. all patches are equally connected to all others (they later relax this assumption by introducing a dispersal kernel). These assumptions allow them to derive expressions for the probabilities of encountering (1) a given species and (2) all the species at once in a randomly given patch: i.e. patch occupancy.

Analytical results for these probabilities and the conditions for persistence that ensue are then complemented with numerical simulations matching the predictions. The authors further analyse empirical data from a metacommunity consisting of 3 species of Daphnia distributed amongst rock pools in a Finnish archipelago. Even though the empirical data is not completely ideal to test all the aspects of the theory (but I guess no dataset ever is!), the data illustrates nicely one of the main focus of the theory: larger landscapes (in the form of larger number of nearby rock pools) increase the probability of coexistence of pairs of species.

The paper is nicely written, and I think provide a nice contribution to theoretical ecology. The authors have taken good care of explaining the rationale for the derivations clearly (although see my specific comments below for suggestions on a few places where this could be improved), which makes the paper very clear.

My only main concern is that the source code and data for the analyses was not provided, which first of all prevented me from being able to assess the validity of the results presented, but more importantly goes against the open science and source code as well as replicability that I believe we all as scientists should strive for. I wouldn’t recommend this paper for publication unless this is addressed and definitely before any decisions are made the code needs to be verified. The source code presented should contain all the instructions to be able to re-create all results and figures in the paper and should be auto-contained and easily runnable on any platform.

Below I also detail a few parts where I think some clarifications would improve the quality of the paper.

Line 54-55. It would be nice to have a quick explanation of why these considerations make the analysis of Eq. 1 possible. Just to guide the reader a bit more and let them know what to expect.

Line 80. A linear function of what? Ni is already a linear function of D anyway in Eq. 1.

Line 83. In expression Ni = N0,i + dN1,i it is not clear why the second term dN1,i means, it is dispersal to the same patch itself? And why doesn’t depend on the other patches as in Eq. 1. I am guessing it is because you are assuming D to be close to 0 but this could be better explained.

Lines 138-139. How many freshwater rock pools? And How many islands?

Line 140. It would be nice to have a figure showing the clustering of the seven group of islands and maybe a map of where they are to give the reader a better sense of the data.

Line 157-158 is repetitive with lines 152-253.

Line 234. The “we can compute patch occupancy analytically” is a bit of a leap of faith here. This would benefit from a more specific explanation. How can you compute the occupancy analytically?

Line 255. This subsection of the results could start acknowledging the shift of focus to the empirical system and presenting a sentence or two re-introducing the empirical question. To make the flow more readable.

Line 285. “the extinction rate can be negative” is confusing. Technically speaking I guess a “negative extinction rate” would imply the spontaneous creation of species, but in any case I don’t think this is what you are trying to argue here since this would mean the local conditions are super suitable. So it would be good to re-write this to avoid confusion.

Line 330. Unimodal rather than unimodel.

Figure 1 caption. What about the r’s? Don’t densities also depend on the values of r?

Figure 2 caption. “suggests values up until = 0.005 are weak.” Values of what? Of d or a? It sounds like it should be d, but this is said just after discussing a, which makes it confusing.

Caption figure 3. The second appearance of “regional equivalence” (referring to the bottom row) should say “regional inequivalence”.

Figure 4. The colours are confusing and not easy to differentiate, especially the 2 pinks, but also the blue and the green.

Figure 4 caption. The title would benefit from more detail: species of what? Where? In which context?

It would also be good to give a sense for what a negative intercept means here.

The thick intervals are defined but the think intervals have been left unexplained. Please correct.

**********

**Have the authors made all data and (if applicable) computational code underlying the findings in their manuscript fully available?**

The PLOS Data policy requires authors to make all data and code underlying the findings described in their manuscript fully available without restriction, with rare exception (please refer to the Data Availability Statement in the manuscript PDF file). The data and code should be provided as part of the manuscript or its supporting information, or deposited to a public repository. For example, in addition to summary statistics, the data points behind means, medians and variance measures should be available. If there are restrictions on publicly sharing data or code —e.g. participant privacy or use of data from a third party—those must be specified.

Reviewer #1: None

Reviewer #2: Yes

Reviewer #3: **No:**  The authors have not made either the data or the code available. This should be fixed before any consideration for publication.

**********

PLOS authors have the option to publish the peer review history of their article (what does this mean?). If published, this will include your full peer review and any attached files.

If you choose “no”, your identity will remain anonymous but your review may still be made public.

**Do you want your identity to be public for this peer review?**  For information about this choice, including consent withdrawal, please see our Privacy Policy.

Reviewer #1: No

Reviewer #2: No

Reviewer #3: No

[NOTE: If reviewer comments were submitted as an attachment file, they will be attached to this email and accessible via the submission site. Please log into your account, locate the manuscript record, and check for the action link "View Attachments". If this link does not appear, there are no attachment files.]

**Figure resubmission:**

After uploading your figures to PLOS’s NAAS tool - https://ngplosjournals.pagemajik.ai/artanalysis, NAAS will process the files provided and display the results in the "Uploaded Files" section of the page as the processing is complete. If the uploaded figures meet our requirements (or NAAS is able to fix the files to meet our requirements), the figure will be marked as "fixed" above. If NAAS is unable to fix the files, a red "failed" label will appear above. When NAAS has confirmed that the figure files meet our requirements, please download the file via the download option, and include these NAAS processed figure files when submitting your revised manuscript.

**Reproducibility:**

To enhance the reproducibility of your results, we recommend that authors of applicable studies deposit laboratory protocols in protocols.io, where a protocol can be assigned its own identifier (DOI) such that it can be cited independently in the future. Additionally, PLOS ONE offers an option to publish peer-reviewed clinical study protocols. Read more information on sharing protocols at https://plos.org/protocols?utm_medium=editorial-email&utm_source=authorletters&utm_campaign=protocols

Attachment Submitted filename: PCOMPBIOL-D-26-00366_comments_1.pdf

# Author comment: Author response to Decision Letter 1

**DOI:** 10.1371/journal.pcbi.1014028.r002

4 Jun 2026

Attachment Submitted filename: Rebuttal.pdf

# Peer review: Decision Letter 1

**DOI:** 10.1371/journal.pcbi.1014028.r003

**Authors:** Zhaolei Zhang, Ricardo Martinez-Garcia

6 Aug 2026

Weak dispersal and landscape size inevitably promote local biodiversity in heterogeneous metacommunities of competing species

PLOS Computational Biology

Dear Dr. De Laender,

Thank you for submitting your manuscript to PLOS Computational Biology. After careful consideration, we feel that it has merit but does not fully meet PLOS Computational Biology's publication criteria as it currently stands. Therefore, we invite you to submit a revised version of the manuscript that addresses the points raised during the review process.

Please submit your revised manuscript by Oct 06 2026 11:59PM. If you will need more time than this to complete your revisions, please reply to this message or contact the journal office at ploscompbiol@plos.org. When you're ready to submit your revision, log on to https://www.editorialmanager.com/pcompbiol/ and select the 'Submissions Needing Revision' folder to locate your manuscript file.

Please include the following items when submitting your revised manuscript:

* A letter that responds to each point raised by the editor and reviewer(s). You should upload this letter as a separate file labeled 'Response to Reviewers'. This file does not need to include responses to formatting updates and technical items listed in the 'Journal Requirements' section below.

* A marked-up copy of your manuscript that highlights changes made to the original version. You should upload this as a separate file labeled 'Revised Manuscript with Track Changes'.

* An unmarked version of your revised paper without tracked changes. You should upload this as a separate file labeled 'Manuscript'.

Kind regards,

Ricardo Martinez-Garcia

Academic Editor

PLOS Computational Biology

Zhaolei Zhang

Section Editor

PLOS Computational Biology

**Additional Editor Comments (if provided):**

The three original reviewers have now assessed the revised manuscript. All three acknowledge the work you have done to address their initial comments and find the manuscript much improved. One important point raised by Reviewer 2, however, remains unresolved. Please fully address whether the model is suitable for predicting pairwise coexistence of the Daphnia species in the metacommunity, either because of or despite pre-emptive competition.

**Reviewers' comments:**

Reviewer's Responses to Questions

**Comments to the Authors:**

**Please note here if the review is uploaded as an attachment.**

Reviewer #1: The authors have carefully revised the manuscript and addressed all of my previous concerns in a thorough and thoughtful manner. I have no further substantive comments. My only suggestion is that the limitation statement, currently rather exhaustive, could be condensed to enhance clarity and flow, as it tends to distract from the main conclusions. With this minor revision, I would be happy to recommend acceptance.

Reviewer #2: Review for manuscript "Weak dispersal and landscape size inevitably promote local biodiversity in heterogeneous metacommunities of competing species" (PCOMPBIOL-D-26-00366R1).

I appreciate the authors’ extensive revision of this manuscript and the supporting information. The current manuscript version poses a substantial structural improvement compared to the original submission and now provides clear definitions for ecological concepts as well as model parameters. Therefore, most of the issues I had previously commented on have been resolved.

To connect the analytical considerations better to the analysis of the dataset of a natural Daphnia metacommunity (responding to my previous major comment 4 and a major comment of Reviewer 1), the authors have added additional model simulations performed and analysed for parameters representative of the Daphnid rock pool metacommunity. This much improved the integration of the empirical system into the overall study design, but nonetheless, the general fit of the dataset to the LV model was addressed only partially.

Specifically, the authors argue that the Daphnid dataset is well suited to their modelling framework (starting in l. 63 of the typeset article), i.e., the LV-competition model in a network-like landscape, mainly focusing on the spatial aspects: because it is a naturally patchy and environmentally heterogeneous habitat network linked by passive dispersal and thus forms a metacommunity. While I agree with this argument, to me, the local interactions incorporated into the analysis and simulations of the LV-model and the dataset on the Daphnia metacommunity seem quite contradictory. Illustrative for this issue, the authors write in the last part of the introduction, only a few sentences apart that “conclusions continue to qualitatively hold when relaxing our analyses’ underlying assumptions, unless interspecific interactions are strong […] (l. 58-59) and then in l. (67-68) that “Our analysis of these data suggests strong interspecific interactions“. Therefore, I’m still not convinced that the dataset is useful to confirm findings from the analysis of the LV model.

The second aspect is the actual assessment of this strong interaction, i.e., the pre-emptive competition in the dataset: It does not become clear why the authors chose to analyse the data set for prevalence of pre-emptive competition as a first objective. Conceptually, I would guess that it makes sense to confirm the assumption made in previous studies (cf. l. 215-218) before parameterising the LV model for simulations with a≥1. However, this or any other reasoning behind the initial data analysis is not spelled out in the Introduction or Methods.

Lastly, it would be helpful to contextualise the empirical system within the model framework used for the analytical considerations. Due to the pre-emptive competition, the Daphnia metacommunity should only correspond to one of the two treated cases of the analysis: the case where the probability for coexistence is estimated for communities that do not coexist in the absence of dispersal, right? The Daphnia dataset thus seems to only represent an edge case of the possible interactions considered in the rest of the study. If I didn’t catch a wrong train of thought here, this should at least be mentioned in the methods or discussed after the results.

To summarise, in my opinion, the question of whether the model is able to predict the pairwise coexistence of the Daphnia species in the rockpool metacommunity because or despite of pre-emptive competition still needs to be addressed.

Otherwise, I have some minor comments on the text and figures:

General comment on Figures: I noticed that all figures currently displayed in the main text show Results, while Methods-related Figures remain in the Supplementary information where most likely, much fewer readers will see them. The original request for conceptual figures was however made to increase accessibility of the methods, i.e. illustrate the Daphnia data and the model assumptions, for the readers. Therefore, I wonder whether it would be possible for the authors to join some of their results figures currently displaying related results (e.g., Figs. 2 and 3 or 4 and 5) to make some space for the Daphnia data map (currently Figure S2) and/or the conceptual figure (currently Figure S1) in the main text Methods?

L. 45: consider rephrasing, since the weak dispersal assumption does not allow the power series expansion, but rather its truncation after the linear term – which then again makes the expression analytically solvable (cf. also l. 48 in Supporting Information, S1.1)

L. 67: consider defining landscape size as the number of habitat patches at first use; especially in combination with ‘local’, it could be mistaken for ‘rock pool’/’patch’ size; or the overall extent of the landscape in which the habitat patches are embedded (which is not considered here)

L. 78-82: Again, I believe the most vital point of the approximation is only implicitly stated: assuming weak dispersal allows you to cut off any nonlinear correction terms; i.e., the weak dispersal assumption implies that only a linear correction term needs to be considered, and any higher order terms are negligible. Please consider adding this implication here.

L. 92: Th mention of “rescue” in brackets seems a bit out of context here – if you want to use it to illustrate the scenario you are considering in this paragraph, please explain the concept in more detail.

L. 112: While I appreciate the clear ecological definition of the feasibility domain in l. 110-111, I wonder whether the technical information given subsequently is necessary/relevant for the further considerations in this study?

L. 124-153: I have the impression that these paragraphs could be written more concisely – for example by integrating the statement starting in l. 148 where you refer to the strict assumptions case into the first paragraph of the simulations section (starting in l. 124), where you explain how you relax these assumptions.

L. 129: I wasn’t sure how the parameter a̅ introduced here relates to the previously introduced interaction parameters a_ij=a or a_ii.

L. 130-131: is this displaying the same information twice? Otherwise, please consider rephrasing to differentiate these cases better

L. 138: please specify: intra- and/or interspecific interactions?

L. 146: please define the parameter c

Computer Code and data (l. 233): in reference to the other reviews of this paper, you may consider re-iterating the availability of your computer code and data here?

L. 279: consider reminding the reader how you define landscape size (see comment above), since it becomes important here

L. 301: if I read the equation correctly, ‘regional density’ is not precise here, since the focal patch l is excluded from the sum in the numerator. Instead, you may stick to the terminology used to describe the numerator of Eq. 8

L. 304: additional assumption for the conclusion from the limiting case a→1 is n≫p. This restriction should be mentioned here for completeness.

L. 305: of the parameter a?

L. 305: ‘this conclusion’ actually, the conclusion/consequence of the first order correction being positive is not explicitly stated here: if this term is positive, then the equilibrium density with dispersal is always larger than the equilibrium density without dispersal, no?

L. 318- 319: this is repetitive to the statement in l. 311, consider merging them

L. 343: intra- or interspecific competition?

L. 394: consider using ‘analyse’ instead of ‘present’, since this data set has been published elsewhere before?

l. 438-439-: Personally, I would define mass effects even more broadly, as dispersal of individuals in such large quantities that they are able to affect local population sizes (regardless of the viability of a local population due to purely local interactions and environment). What is described starting in l. 438 I would call a rescue effect, and thus only a special case of mass effect dynamics, where dispersal allows persistence of an otherwise non-viable population. Mass effects often jointly work with local dynamics, and seldomly overrule them completely. Please consider rephrasing this for clarity!

L. 443-444: in l. 443, you say that dispersal needs to be sufficiently high, in the next sentence, you say that dispersal needs not to be high. I think I understand what you mean here but you may consider rephrasing for clarity.

L. 467-469: I believe this statement of the discussion could use a literature reference

L. 510: add ‘is’ after ‘what’

Reviewer #3: The authors have addressed all my main comments and I am happy with that. I would however recommend that the authors put a little more effort into developing a more informative readme file for the repository with specific instructions on how to run the code and reproduce exactly the results from the paper. It would be useful to have a set of tutorial-like instructions to do this and an automated pipeline that reproduces a basic version of the results.

This will improve reproducibility of the results presented in the paper and would also make life easier for those wanting to use this code, thus improving open code and data practices.

**********

**Have the authors made all data and (if applicable) computational code underlying the findings in their manuscript fully available?**

The PLOS Data policy requires authors to make all data and code underlying the findings described in their manuscript fully available without restriction, with rare exception (please refer to the Data Availability Statement in the manuscript PDF file). The data and code should be provided as part of the manuscript or its supporting information, or deposited to a public repository. For example, in addition to summary statistics, the data points behind means, medians and variance measures should be available. If there are restrictions on publicly sharing data or code —e.g. participant privacy or use of data from a third party—those must be specified.

Reviewer #1: None

Reviewer #2: Yes

Reviewer #3: Yes

**********

PLOS authors have the option to publish the peer review history of their article (what does this mean?). If published, this will include your full peer review and any attached files.

If you choose “no”, your identity will remain anonymous but your review may still be made public.

**Do you want your identity to be public for this peer review?**  For information about this choice, including consent withdrawal, please see our Privacy Policy.

Reviewer #1: No

Reviewer #2: No

Reviewer #3: No

[NOTE: If reviewer comments were submitted as an attachment file, they will be attached to this email and accessible via the submission site. Please log into your account, locate the manuscript record, and check for the action link "View Attachments". If this link does not appear, there are no attachment files.]

**Figure resubmission:**

**Reproducibility:**

?>

# Author comment: Author response to Decision Letter 2

**DOI:** 10.1371/journal.pcbi.1014028.r004

3 Sep 2026

Attachment Submitted filename: Rebuttal_auresp_2.pdf

# Peer review: Decision Letter 2

**DOI:** 10.1371/journal.pcbi.1014028.r005

**Authors:** Zhaolei Zhang, Ricardo Martinez-Garcia

4 Oct 2026

Dear Prof. De Laender,

We are pleased to inform you that your manuscript 'Weak dispersal and landscape size inevitably promote local biodiversity in heterogeneous metacommunities of competing species' has been provisionally accepted for publication in PLOS Computational Biology.

Before your manuscript can be formally accepted you will need to complete some formatting changes, which you will receive in a follow up email. A member of our team will be in touch with a set of requests.

Please note that your manuscript will not be scheduled for publication until you have made the required changes, so a swift response is appreciated.

IMPORTANT: The editorial review process is now complete. PLOS will only permit corrections to spelling, formatting or significant scientific errors from this point onwards. Requests for major changes, or any which affect the scientific understanding of your work, will cause delays to the publication date of your manuscript.

Should you, your institution's press office or the journal office choose to press release your paper, you will automatically be opted out of early publication. We ask that you notify us now if you or your institution is planning to press release the article. All press must be co-ordinated with PLOS.

Thank you again for supporting Open Access publishing; we are looking forward to publishing your work in PLOS Computational Biology.

Best regards,

Ricardo Martinez-Garcia

Academic Editor

PLOS Computational Biology

Zhaolei Zhang

Section Editor

PLOS Computational Biology

***********************************************************

Reviewer's Responses to Questions

**Comments to the Authors:**

**Please note here if the review is uploaded as an attachment.**

Reviewer #2: I appreciate the author’s response to my comments and further revision of the manuscript, which satisfyingly addressed the remaining concerns. Thus, I can recommend this manuscript for publication without any further comments.

**********

**Have the authors made all data and (if applicable) computational code underlying the findings in their manuscript fully available?**

The PLOS Data policy requires authors to make all data and code underlying the findings described in their manuscript fully available without restriction, with rare exception (please refer to the Data Availability Statement in the manuscript PDF file). The data and code should be provided as part of the manuscript or its supporting information, or deposited to a public repository. For example, in addition to summary statistics, the data points behind means, medians and variance measures should be available. If there are restrictions on publicly sharing data or code —e.g. participant privacy or use of data from a third party—those must be specified.

Reviewer #2: Yes

**********

PLOS authors have the option to publish the peer review history of their article (what does this mean?). If published, this will include your full peer review and any attached files.

If you choose “no”, your identity will remain anonymous but your review may still be made public.

**Do you want your identity to be public for this peer review?**  For information about this choice, including consent withdrawal, please see our Privacy Policy.

Reviewer #2: No

# Editor report: Acceptance letter

**DOI:** 10.1371/journal.pcbi.1014028.r006

**Authors:** Zhaolei Zhang, Ricardo Martinez-Garcia

PCOMPBIOL-D-26-00366R2

Weak dispersal and landscape size inevitably promote local biodiversity in heterogeneous metacommunities of competing species

Dear Dr De Laender,

I am pleased to inform you that your manuscript has been formally accepted for publication in PLOS Computational Biology. Your manuscript is now with our production department and you will be notified of the publication date in due course.

The corresponding author will soon be receiving a typeset proof for review, to ensure errors have not been introduced during production. Please review the PDF proof of your manuscript carefully, as this is the last chance to correct any errors. Please note that major changes, or those which affect the scientific understanding of the work, will likely cause delays to the publication date of your manuscript.

Soon after your final files are uploaded, unless you have opted out, the early version of your manuscript will be published online. The date of the early version will be your article's publication date. The final article will be published to the same URL, and all versions of the paper will be accessible to readers.

For Research, Software, and Methods articles, you will receive an invoice from PLOS for your publication fee after your manuscript has reached the completed accept phase. If you receive an email requesting payment before acceptance or for any other service, this may be a phishing scheme. Learn how to identify phishing emails and protect your accounts at https://explore.plos.org/phishing.

Thank you again for supporting PLOS Computational Biology and open-access publishing. We are looking forward to publishing your work!

With kind regards,

Sharmila Kamatchi

PLOS Computational Biology | Carlyle House, Carlyle Road, Cambridge CB4 3DN | United Kingdom ploscompbiol@plos.org | Phone +44 (0) 1223-442824 | ploscompbiol.org | @PLOSCompBiol
